# TAL1 is indispensable in ETV2-primed human endothelial cell fate determination

**DOI:** 10.1126/sciadv.aef9769

**Published:** 2026-08-12

**Authors:** Yun Zhao, Mengze Sun, Zixuan Hong, Jingkun Yi, Donglin Yu, Xiaojing Ma, Liangliang Tian, Yi Hong, Lijun Sun, Xiaoxia Li, Yuan Zhou, Kailong Li, Xi Wang, Kai Wang

**Affiliations:** ^1^ Department of Physiology and Pathophysiology, School of Basic Medical Sciences, State Key Laboratory of Vascular Homeostasis and Remodeling, Clinical Stem Cell Research Center, Peking University Third Hospital, Beijing Advanced Center of Cellular Homeostasis and Aging-Related Diseases, Peking University, Beijing 100191, China.; ^2^ Department of Biomedical Informatics, School of Basic Medical Sciences, State Key Laboratory of Vascular Homeostasis and Remodeling, Peking University, Beijing 100191, China.; ^3^ Department of Biochemistry and Molecular Biology, Beijing Key Laboratory of Protein Posttranslational Modifications and Cell Function, School of Basic Medical Sciences, Peking University Health Science Center, Beijing 100191, China.; ^4^ State Key Laboratory of Female Fertility Promotion, Department of Obstetrics and Gynecology, Peking University, Beijing 100191, China.; ^5^ Beijing Key Laboratory of Cardiovascular Precision Repair and Functional Reconstruction, Beijing Anzhen Hospital, Beijing 101118, China.

## Abstract

ETS variant transcription factor 2 (ETV2) serves as a foundational transcription factor for endothelial lineage specification. However, the lineage-specific cofactors that orchestrate with ETV2 during endothelial fate commitment remain elusive. Here, we demonstrate that ETV2 drives the rapid forward programming of human pluripotent stem cells (hPSCs) into endothelial cells (ECs) by direct remodeling of endothelial-specific enhancers. Crucially, we identify T cell acute lymphocytic leukemia protein 1 (TAL1), which is traditionally characterized as a hematopoietic regulator, as an indispensable cofactor for ETV2-mediated endothelial commitment. Distinct from its role in murine development, TAL1 deficiency in hPSCs not only aborts the endothelial program by impairing H3K27ac deposition at key enhancers but also triggers a profound lineage redirection toward a mesenchymal fate. Mechanistically, TAL1 physically interacts with ETV2 to recruit the p300, thereby facilitating a permissive chromatin environment for endothelial identity. By leveraging an hPSC-based differentiation model, our findings establish TAL1 as a master gatekeeper of human EC specification and provide a molecular blueprint for how ETV2-centric complexes synergistically govern human cell fate.

## INTRODUCTION

Blood vessels formed by endothelial cells (ECs) and perivascular mural cells (MCs) are essential for the maintenance and function of organs, tissues, and cells. The proper formation, function, and maintenance of blood vessels are crucial for sustaining homeostasis within the body. The failure to maintain homeostatic status of blood vessels causes lethal diseases throughout the body, such as ischemic heart disease and limb ischemia (*1*, *2*). Thus, understanding of the mechanisms that govern vascular and endothelial lineage determination and developing treatment strategies, including cell replacement therapy, are important to tackle the considerable morbidity and mortality caused by the vascular disease.

Human pluripotent stem cells (hPSCs) hold great promise for investigating development and cell replacement therapy (*3*). Compared with traditional differentiation strategy via orchestration of multiple signaling pathways, forward programming has been proved to be more efficient to induce cell fate determination via overexpression of certain transcription factors (TFs). This approach has successfully directed the differentiation of hPSCs into various cell types, including neurons, astrocytes, and cardiomyocytes (*4*–*6*). Furthermore, forward programming strategy enables simultaneous generation of ECs and MCs via activation of ETS variant transcription factor 2 (ETV2) and NKX3.1 for the construction of functional vascular organoids, offering a previously unexplored path for the rapid generation of complex organoids (*7*). However, the inherent differences between hPSCs and their target cell types often render individual TFs insufficient to drive cell fate commitment. For instance, although myogenic differentiation 1 (MYOD1) is a master regulator of skeletal muscle cells, successful forward programming of skeletal muscle cells requires the coexpression of BAF60C or JMJD3 along with MYOD1 (*8*–*10*). Similarly, the astrocyte master regulator nuclear factor I B (NFIB) needs to collaborate with SRY-box transcription factor 9 (SOX9) to efficiently drive astrocyte differentiation (*11*). These examples underscore the necessity of cooperation between master TFs and cofactors to activate cell type–specific gene regulatory networks and establish a stable cell fate, highlighting the importance of deciphering the molecular mechanisms of forward programming to facilitate in vitro differentiation for disease modeling and cell replacement therapy.

ETV2 has been identified as a critical TF in the development of endothelial lineages (*12*). In the developing mouse embryo, Etv2 is transiently activated in progenitor cells during embryonic day 7.75 (E7.75) to E9.5. Constitutive knockout (KO) of Etv2 results in complete loss of vessels and blood cells, leading to embryonic lethality by E9.5 (*13*). In addition, Etv2 has been recognized as a downstream target of vascular endothelial growth factor (VEGF) signaling and regulates the expression of several key TFs (*14*). Recent studies in mouse embryonic stem cells (mESCs) have further elucidated that Etv2 functions as a bona fide pioneer factor recruiting Brg1 and Baf155 to remodel chromatins around endothelial genes and maintaining an open chromatin configuration (*15*, *16*), and Etv2 can target *Mesp1* to repress the cardiac lineage (*17*). By deciphering the accessibility and H3K27ac deposition around Etv2 binding sites in Pdgfrα^+^Flk1^+^ progenitor cells, a previous study identified that ETV2-bound enhancer activation is a key event driving hematoendothelial fate commitment (*18*). Besides its role in driving endothelial differentiation, the activation of ETV2 in ECs could also remodel the chromatin to reset the ECs to adaptable and vasculogenic cells (*19*). Because of its critical role in EC differentiation, ETV2 has been used to facilitate the generation of ECs. Overexpression of ETV2 directly converted human fibroblasts into functional ECs (*20*–*24*). Our previous study demonstrated that transient activation of ETV2 in hPSCs or mesoderm progenitor cells (MPCs) via chemically modified mRNA effectively generates ECs, highlighting the potential of ETV2 for inducing endothelial differentiation (*25*). Furthermore, ETV2 is more powerful than MYOD1 and NFIB, which can individually drive EC differentiation without coexpression of other TFs, raising the interest to explore how the downstream cofactors are activated and recruited during this process.

In this study, we used a piggyBac-based ETV2 induction system in hPSCs to systematically dissect the transcriptional dependencies of human endothelial lineage specification. By capturing the high-resolution transcriptomic and epigenetic dynamics during this transition, we defined a repertoire of ETV2-regulated endothelial-specific enhancers, including those associated with SOX17 and NOTCH4, which drive an arterial-biased program. Notably, we identify the canonical hematopoietic regulator T cell acute lymphocytic leukemia protein 1 (TAL1) as a requisite cofactor that functions in concert with ETV2 to ensure successful lineage commitment. TAL1 deficiency not only aborts endothelial differentiation but also destabilizes the lineage-specific chromatin landscape. Integrated genomic analysis of TAL1 and p300 binding sites further reveals that TAL1 acts as a molecular bridge by recruiting p300 to prime and activate enhancers. Collectively, our work identifies a ETV2-TAL1 regulatory axis that governs human endothelial fate, providing a mechanistic framework for the precision engineering of vascular lineages via hPSC-based models.

## RESULTS

### ETV2 induced rapid and sequential programming of hPSC into ECs

**Fig. 1.**
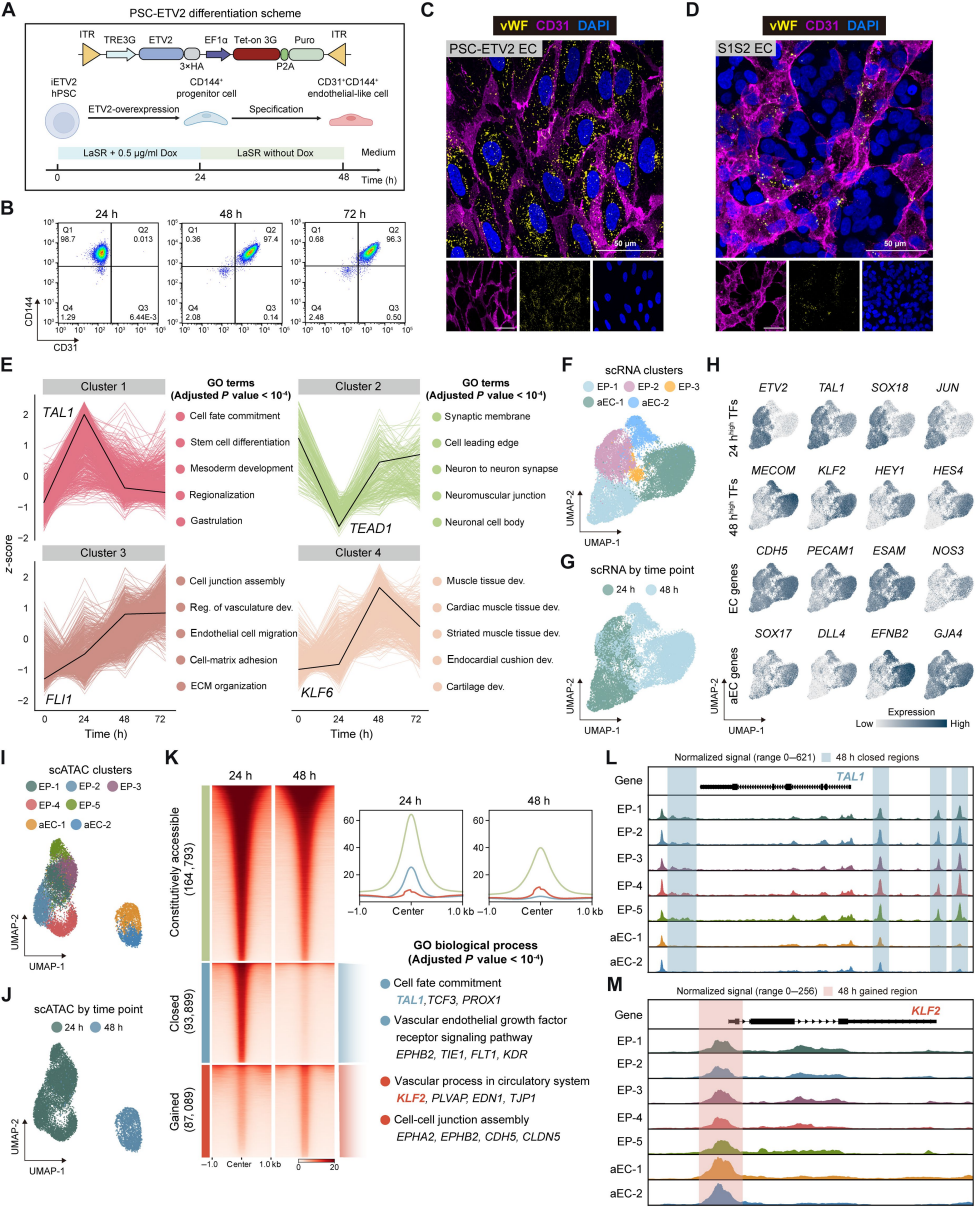
ETV2 orchestrates rapid and sequential endothelial specification in human pluripotent stem cell. (**A**) Schematic representation of the PSC-ETV2 differentiation method. (**B**) Flow cytometric analysis of the expression level of CD31 and CD144 at three time points. (**C**) Representative immunofluorescence images of CD31 and vWF in PSC-ETV2 endothelial cells (ECs). (**D**) Representative immunofluorescence images of CD31 and vWF in S1S2 ECs. (**E**) The dynamic changes of gene expression levels across clusters and the associated GO functional terms. All the displayed terms have adjusted *P* value <10^−4^. (**F**) Uniform manifold approximation and projection (UMAP) visualization of PSC-ETV2-24h and PSC-ETV2-48h single-cell RNA sequencing (scRNA-seq) data, colored by unsupervised clustering. (**G**) UMAP of cells colored according to time points. (**H**) UMAP visualization of the expression of key transcription factors and EC marker genes. (**I**) UMAP visualization of single-cell sequencing assay for transposase-accessible chromatin (scATAC-seq) data, colored by unsupervised clustering. (**J**) UMAP of scATAC-seq data colored according to time points. (**K**) Heatmap showing the differentially accessible peaks and the related GO terms. All the displayed terms have adjusted *P* value <10^−4^. (**L**) Genome browser views showing 48-hour closed regions surrounding *TAL1*. (**M**) Genome browser views showing 48-hour gained regions surrounding *KLF2*. Scale bar, 50 μm in (C) and (D).

To achieve stable and controllable expression of ETV2 in hPSCs, we used the piggyBac all-in-one inducible expression system and generated a stable *iETV2*-*3×HA* cell line in H1 hPSCs (ETV2-H1 cells) (Fig. 1A). The clonal ETV2-H1 cells could efficiently overexpress ETV2 after administration of doxycycline and maintain the expression of pluripotency markers OCT4 and NANOG (fig. S1, A and B). Leveraging this platform, we developed a streamlined PSC-ETV2 programming protocol, where a 24-hour pulse of ETV2 was sufficient to trigger the endothelial program. This transient ETV2 activation induced a rapid fate transition, with cells transitioning into CD144^+^ progenitors within the first 24 hours and maturing into a homogeneous CD31^+^CD144^+^ EC population within 48 hours (Fig. 1B). This endothelial identity remained stable and was further validated by the robust expression of endothelial function–related genes vWF compared with EC generated using the traditional EC differentiation protocol (S1S2) (Fig. 1, C and D). These data establish the iETV2-H1 system and PSC-ETV2 protocol as a highly efficient model for dissecting the molecular kinetics of human endothelial specification.

To further investigate the molecular dynamics of ETV2-induced reprogramming in hPSCs, we performed RNA sequencing (RNA-seq) and characterized the transcriptome profile of the cells post-ETV2 induction during this differentiation process at 24, 48, and 72 hours, named PSC-ETV2-24h, PSC-ETV2-48h, and PSC-ETV2-72h cells, respectively. Principal components analysis (PCA) revealed the clustering of hPSCs and PSC-ETV2 cells at different time points. The PSC-ETV2-48h and PSC-ETV2-72h cell clusters were closely aligned but distinct from the hPSC and PSC-ETV2-24h cell clusters, suggesting that lineage specification was achieved by 48 hours (fig. S1C). Hierarchical clustering of differentially expressed genes (DEGs) formed four groups. The DEGs of these cells during differentiation further confirmed the similarity between the PSC-ETV2-48h and PSC-ETV2-72h cells and their differences from both the hPSCs and PSC-ETV2-24h cells (fig. S1D). Cluster 1 showed genes (e.g., *TAL1*) that were up-regulated in the first 24 hours but down-regulated thereafter (Fig. 1E). Gene ontology (GO) enrichment analysis of cluster 1 genes identified as important for cell fate commitment (e.g., *TAL1*, *GATA2*, and *GATA3*), stem cell differentiation (e.g., *EOMES*, *SHH*, and *TBX1*), and mesoderm development (e.g., *BMP4*, *EOMES*, and *ETV2*) was carried out, indicating the potential role of these genes as pioneer factors in EC fate specification (Fig. 1E). In contrast, cluster 3 included genes (e.g., *FLI1*) that were consistently up-regulated at 24, 48, and 72 hours after the induction of ETV2. These genes are associated with EC functions, including cell junction assembly (e.g., *CLDN5*, *CDH5*, and *ESAM*) and EC migration (e.g., *KLF4*, *MET*, and *EFNB2*). GO enrichment analysis of DEGs between hPSC and PSC-ETV2-24h cells, as well as between PSC-ETV2-48h and PSC-ETV2-24h cells, also revealed pathways related to EC differentiation (e.g., *ETV2*, *SOX17*, *SOX18*, *ZEB1*, and *NOTCH4*) and EC function (e.g., *KDR*, *NRP1*, *VCAM1*, and *EPHA3*) (fig. S1, E and F).

To explore the dynamics of TF expression during EC fate commitment, we analyzed the expression levels of typical EC-related TFs and validated the expression via reverse transcription quantitative polymerase chain reaction (RT-qPCR) (fig. S1, G and H). Canonical EC-identity TFs, including *ERG*, *FLI1*, *SOX7*, and *ELK3*, exhibited progressive up-regulation peaking at 48 hours. Notably, the recently identified vascular regulator MECOM showed a marked induction at 48 hours after being nearly undetectable at 24 hours (*26*). While the lymphatic-associated factor *SOX18* was transiently expressed at 24 hours, its subsequent decline coincided with the robust emergence of arterial-specific markers *HEY1* and *SOX17*, suggesting that the PSC-ETV2 method predominantly directs hPSCs toward an arterial-like identity. The rapid down-regulation of *ETV2* following Dox withdrawal at 24 hours effectively mimics the endogenous transient pulse of ETV2 observed during embryonic vasculogenesis.

To resolve the cellular heterogeneity and transition dynamics during ETV2-mediated programming, we performed single-cell RNA sequencing (scRNA-seq) at 24 and 48 hours postinduction. Unsupervised clustering and visualization revealed a clear developmental trajectory consisting of five major clusters (Fig. 1, F and G, and fig. S2A). The 24-hour population was predominantly composed of three endothelial progenitor (EP) clusters, characterized by the hallmark expression of *ETV2* and *TAL1*. By 48 hours, these cells mainly transitioned into two distinct arterial endothelial cell (aEC) clusters, as evidenced by the robust activation of *MECOM*, *HEY1*, *HES4*, *SOX17*, *DLL4*, and *EFNB2* (Fig. 1H). Notably, integrated analysis demonstrated that the intrapopulation heterogeneity within both EP and aEC compartments was primarily driven by cell cycle phase variance rather than divergent lineage fates (fig. S2, B to D). To further dissect the epigenetic transition during ETV2-mediated EC differentiation, we performed single-cell sequencing assay for transposase-accessible chromatin (scATAC-seq) at 24 and 48 hours postinduction. Clustering of these cells revealed five EP clusters and two aEC clusters (Fig. 1, I and J, and fig. S2E). However, the high correlation observed among EP clusters and between aEC clusters (*r* > 0.85) suggests an absence of intrinsic differences within these groups (fig. S2F). Furthermore, the ATAC gene scores of several marker genes of EP cells and aECs are consistent between the two time points, indicating that there is no significant difference on the chromatin accessibility of these genes (fig. S2G). These data further supported the highly homogeneous cell identity during the PSC-ETV2 differentiation process.

Upon analyzing differentially accessible peaks, we found that nearly half of the sites remain accessible at both 24 and 48 hours. Notably, 48-hour cells exhibited a marked reduction in accessibility at 93,899 sites whereas 87,089 sites showed a slight increase (Fig. 1K). Peak-to-gene annotation and GO enrichment analysis revealed that these closing peaks are associated with cell fate commitment (*TAL1*, *TCF3*, and *PROX1*) as well as VEGF signaling. Conversely, peaks with increased accessibility are linked to vascular functions (*KLF2*, *PLVAP*, *EDN1*, and *TJP1*) (Fig. 1K). Genome browser tracks around *TAL1* displayed a substantial decrease in accessibility at three putative enhancers upstream of its promoter, aligning with the down-regulation of *TAL1* transcripts at 48 hours (Fig. 1L). Similarly, the ATAC signal at the *KLF2* promoter increased over time, consistent with the specific up-regulation of KLF2 at 48 hours (Fig. 1M). Collectively, these findings underscore that the PSC-ETV2 method facilitates a highly synchronized and nearly homogeneous fate commitment, providing a robust platform for dissecting the regulatory mechanisms of human EC specification.

### ETV2 established endothelial enhancer repertoire within 24 hours

**Fig. 2.**
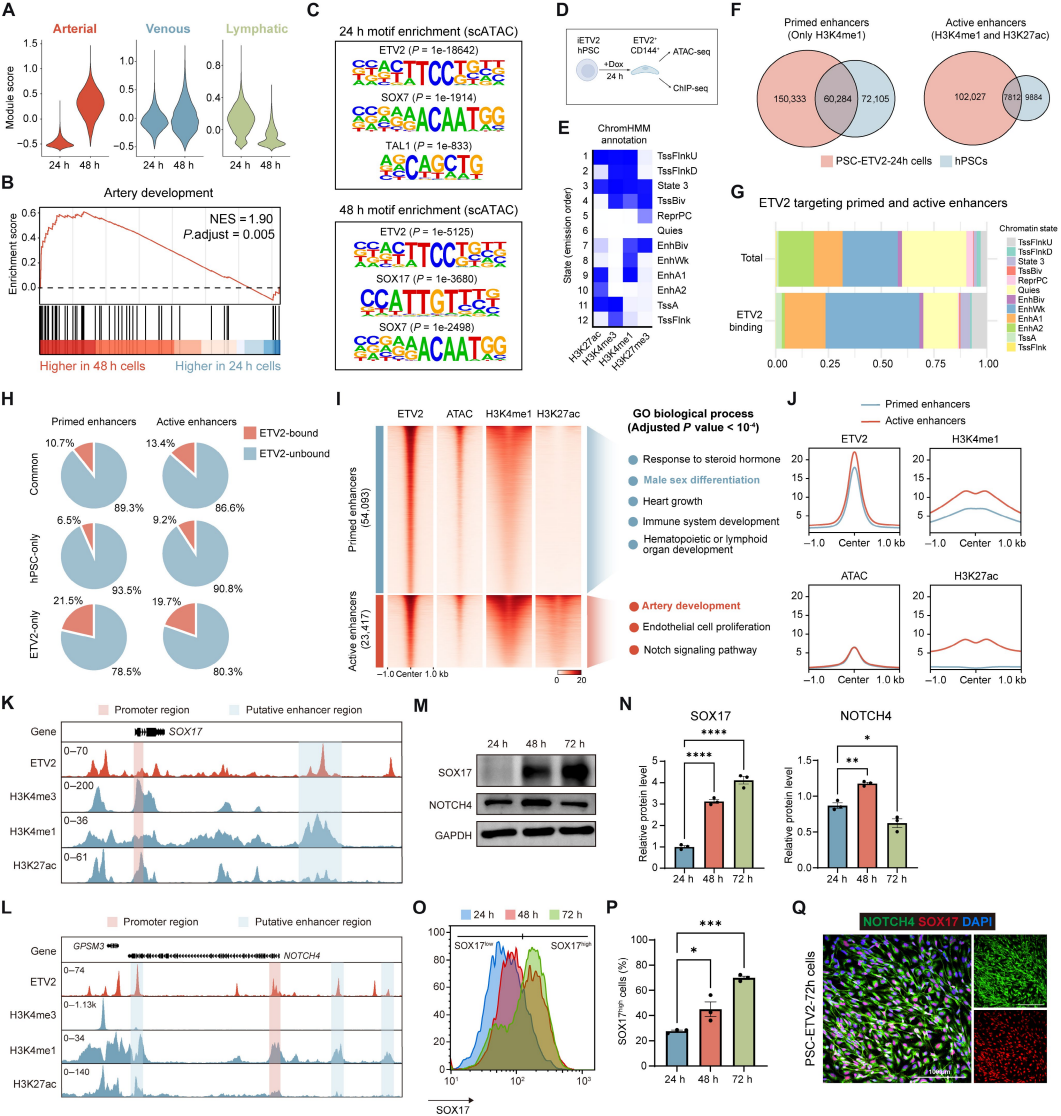
ETV2 primes arterial endothelial cell fate via enhancer targeting. (**A**) Violin plots showing module scores for endothelial cell subtypes in PSC-ETV2 cells at 24 and 48 hours. (**B**) Gene set enrichment analysis for artery development comparing 48-hour versus 24-hour PSC-ETV2 cells. (**C**) Motif enrichment analysis of scATAC-seq data at 24 and 48 hours. (**D**) Schematic representation of the epigenetic sequencing strategy. (**E**) ChromHMM annotation of the chromatin states in PSC-ETV2-24h cells. (**F**) Venn diagrams showing the overlap between PSC-ETV2-24h cells and hPSCs on poised enhancers (left) or active enhancers (right). (**G**) Preference of ETV2 binding sites toward different chromatin states. (**H**) Percentage of ETV2-bound and unbound enhancers. (**I**) Heatmaps of ATAC, H3K4me1, and H3K27ac signals centered at ETV2-binding sites with enriched GO terms (adjusted *P* value <10^−4^). (**J**) Average signal of ETV2, ATAC, H3K4me1, and H3K27ac at ETV2-bound poised enhancers or active enhancers. (**K**) Genome browser views showing ETV2 binding sites surrounding *SOX17*. (**L**) Genome browser views showing ETV2 binding sites surrounding *NOTCH4*. (**M**) Representative Western blot imaging showing the expression level of SOX17 and NOTCH4 at three time points. (**N**) Quantification of the protein level of SOX17 and NOTCH4 (*n* = 3). (**O**) Flow cytometric analysis of the expression level of SOX17 at three time points. (**P**) Quantification of the ratio of SOX17^high^ cells (*n* = 3). (**Q**) Representative immunofluorescence images of SOX17 and NOTCH4 in PSC-ETV2-72h cells. Scale bar, 100 μm in (Q). All data are presented as mean ± SEM. *n* indicates biological replicates [(N) and (P)]. Statistical significance was assessed by one-way ANOVA with Tukey’s post hoc test [(N) and (P)]. The level of significance was labeled as ns, ^*^, ^**^, ^***^, and ^****^, denoting nonsignificant and *P* value of <0.05, <0.01, <0.001, and <0.0001, respectively.

To rigorously define the EC identity achieved via ETV2 programming, we applied lineage-specific gene modules derived from established benchmarks (*27*, *28*). The arterial module score showed a robust and significant elevation at 48 hours, whereas venous and lymphatic scores remained at baseline (Fig. 2A and fig. S3, A to D). This arterial bias was further corroborated by gene set enrichment analysis (GSEA) and motif enrichment analysis of scATAC-seq datasets, the latter of which identified SOX17 as a core active TF in 48 hours cells (Fig. 2, B and C). The SOXF family TFs, including SOX7, SOX17, and SOX18, are known to be closely involved in EC differentiation and subsequent specification toward arterial, venous, and lymphatic lineages (*27*). In particular, SOX17 has been identified as an indispensable TF for both the acquisition and maintenance of aEC identity (*29*, *30*). In our system, we observed that SOX17 expression remains up-regulated along the pseudotime trajectory. Conversely, SOX18, which is associated with lymphatic ECs, stays down-regulated. Meanwhile, SOX7 expression remains consistently high throughout the entire process, aligning with its role as a TF expressed across all EC lineages (fig. S3E). To decipher the epigenetic blueprint governing this rapid specification toward aEC, we integrated chromatin immunoprecipitation sequencing (ChIP-seq; for ETV2 and histone modifications) with ATAC-seq to map the chromatin landscape within the initial 24-hour window (Fig. 2D). ChromHMM-based state modeling identified a massive expansion of the regulatory repertoire, characterized by the de novo establishment of approximately 150,000 primed (H3K4me1 only) and 100,000 active (H3K4me1/H3K27ac) enhancers in PSC-ETV2-24h cells (Fig. 2, D to F) (*31*). Crucially, ETV2-binding events were highly enriched at these nascent enhancers, with a preference for regions transitioning from a quiescent state in hPSCs to an active or primed state in progenitors (Fig. 2, G and H, and fig. S3F). Furthermore, we compared our results with published CUT&RUN datasets to validate the fidelity. This comparison revealed consistent ETV2 binding sites and histone modification patterns at two key EC genes, *ESAM* and *NECTIN2* (fig. S3, G and H). Such alignment between our data and established resources further demonstrates the high reliability of our epigenetic profiles of PSC-ETV2-24h cells.

Beyond its extensive distal regulatory role, we observed that approximately 13% of ETV2-binding events occurred at proximal promoter regions, where ETV2 exhibited a notably higher binding intensity compared to its enhancer-bound counterparts (fig. S3, I and J). These ETV2-occupied promoters were highly enriched for H3K4me3, indicating a transition to an active transcriptional state following ETV2 recruitment (fig. S3J). However, the epigenetic signatures and downstream expression patterns across these targets were not uniform. For instance, the promoters of key endothelial TFs *ELK3* and *MECOM* both displayed ETV2 occupancy and H3K27ac deposition, yet *ELK3* exhibited significantly higher H3K4me3 levels (fig. S3, K and L). This disparity correlated with divergent expression kinetics along the pseudotime: while *ELK3* was robustly maintained from 24 to 48 hours, *MECOM* expression was specifically restricted to 48 hours (fig. S3, M and N). Collectively, these data suggest that ETV2 targets both enhancers and promoters to initiate the endothelial program.

### ETV2 primed arterial EC fate via enhancer targeting

To elucidate the functional output of ETV2-mediated enhancer remodeling, we annotated the de novo ETV2-bound enhancers based on their target gene proximity. GO enrichment analysis revealed a divergence between chromatin states: primed enhancers were predominantly associated with alternative lineage potentials, such as male sex differentiation, heart growth, and immune system development, whereas active enhancers were highly enriched for artery development and Notch signaling (Fig. 2I). Notably, while these two enhancer populations exhibited comparable chromatin accessibility, active enhancers displayed a significantly higher ETV2 binding affinity (Fig. 2J).

We next examined the transcriptional consequences of this epigenetic priming. Arterial-related genes showed robustly higher expression levels compared to alternative lineage genes (fig. S4A). Motif analysis reveals enrichment of androgen receptor (AR) motif on male sex differentiation-related active enhancers and TAL1 and AP-1 motif enrichment on artery development-related active enhancers, indicating the potential regulator of the competence of arterial EC and germ cell fate (fig. S4B). However, key TFs associated with male sex differentiation, such as AR and SOX9, exhibit only basal level expression, while NR5A1 and WT1 show no significant expression (fig. S4C). These observations suggest that although the AR motif is enriched within active enhancers related to male sex differentiation, the failure to activate the corresponding TF network prevents the cells from adopting alternative cell fates. To explore how ETV2 efficiently activates aEC genes and directs hPSC toward aEC fates, we visualized genomic tracks of PSC-ETV2-24h cells, focusing on typical arterial EC marker genes such as *DLL4*, *GJA4*, *SOX17*, and *NOTCH4*. For *DLL4* and *GJA4*, we found a significant promoter targeting preference of ETV2 accompanied by H3K4me3 deposition, which indicates the direct activation of these genes (fig. S4, D and E). In contrast, H3K4me3 was only slightly deposited on the *SOX17* and *NOTCH4* promoters at 24 hours. This observation is consistent with the high transcription levels of *SOX17* and *NOTCH4* observed at 48 hours rather than 24 hours (Fig. 2, K and L, and fig. S4, F and G). Notably, upon ETV2 overexpression, several putative active enhancers were established surrounding the ETV2 binding sites, suggesting that ETV2 exerts priming effects on the arterial EC fate (Fig. 2, K and L). Conversely, venous EC-related genes, such as *NT5E* and *APLNR*, also have ETV2 binding sites but exhibit relatively low enrichment of H3K27ac (fig. S4, H and I). This early epigenetic priming preceded the peak of SOX17 and NOTCH4 protein expression, which was validated by Western blot to reach maximal levels at 48 or 72 hours (Fig. 2, M and N). In addition, the proportion of SOX17^high^ cells continued to increase from 24 to 72 hours, which is consistent with the protein level (Fig. 2, O and P). Immunostaining on SOX17 and NOTCH4 confirmed the homogeneous expression in PSC-ETV2-72h cells (Fig. 2Q). Collectively, these data demonstrate that ETV2 initiates human arterial fate by establishing a de novo enhancer landscape that prioritizes vascular programs over alternative developmental trajectories.

### Predifferentiation of hPSCs to MPCs restricts ETV2 binding sites

Previous studies demonstrated that the timing of ETV2 activation affects the transcriptional profile of ECs, thereby affecting the function (*4*, *25*). Activation of ETV2 in MPCs (hereafter MPC-ETV2 method) generated functionally competent ECs capable of forming a perfused vascular network with smooth muscle cell upon transplantation into the subcutaneous region in mice, whereas the ECs generated using the PSC-ETV2 method failed to show similar functions (*25*). To investigate how the initial cell state affects the functional properties of ECs, we performed RNA-seq in both PSC-ETV2 and MPC-ETV2 methods (fig. S5A). Differential gene expression analysis revealed that genes related to mesoderm development (e.g., *LEF1* and *ZEB2*) were up-regulated in MPC-ETV2-24h cells, while pluripotent-related genes, such as *POU5F1*, were down-regulated (fig. S5B). In addition, GSEA on angiogenesis gene sets showed significant enrichment of EC function-related genes (e.g., *NOS3*, *APLNR*, *ANGPT1*, and *ANGPT2*) and higher expression levels in MPC-ETV2-24h cells compared to PSC-ETV2-24h cells, correlating with the superior vascularization potential of these ECs generated by the MPC-ETV2 method (fig. S5, C and D). Notably, activation of ETV2 in hPSCs led to the expression of neuron-related genes, which were less pronounced in endothelial colony-forming cells (ECFCs), suggesting that these genes are likely non-EC specific (fig. S5, E and F).

Next, we profiled the ETV2 binding sites in MPC-ETV2-24h cells to investigate whether ETV2 regulates these non-EC genes. In total, we identified 44,824 PSC-ETV2-specific peaks (fig. S5G). Peak-to-gene annotation and GO enrichment analysis revealed that these potential ETV2-regulated genes were associated with pathways such as synapse organization and axonogenesis (fig. S5H). Moreover, motif enrichment analysis revealed that the PSC-ETV2–specific peaks were enriched for the POU family motif (fig. S5I). POU3F3, a TF associated with neuronal development (*32*), was identified. Another TF related to astrocyte differentiation, PITX1 (*33*), showed 71.02% coverage of these PSC-ETV2–specific sites. This further supports the possibility that ETV2 could activate neuron-related genes in hPSCs. In addition, MPC-ETV2-24h cells displayed stronger H3K27ac signals at ETV2 binding sites compared to PSC-ETV2-24h cells (fig. S5J). A further comparison of angiogenesis-related genes revealed that MPC-ETV2-24h cells also exhibited higher H3K27ac signals at ETV2 binding sites compared to PSC-ETV2-24h cells, though no differences in ETV2 binding strength were observed within these two groups (fig. S5K). Genome browser views of two up-regulated genes in MPC-ETV2-24h cells, *NOS3* and *HOXB3*, showed increased H3K27ac deposition around ETV2 binding sites in the MPC-ETV2 cell group (fig. S5, L and M). These findings suggest that predifferentiation of hPSCs into MPCs could restrict ETV2 binding to certain sites, preventing the activation of ectoderm-related genes, while promoting histone acetylation of ETV2-regulated EC function-related genes to safeguard the generation of functionally competent ECs.

### Integrative analysis reveals TAL1 as a potential master regulator

**Fig. 3.**
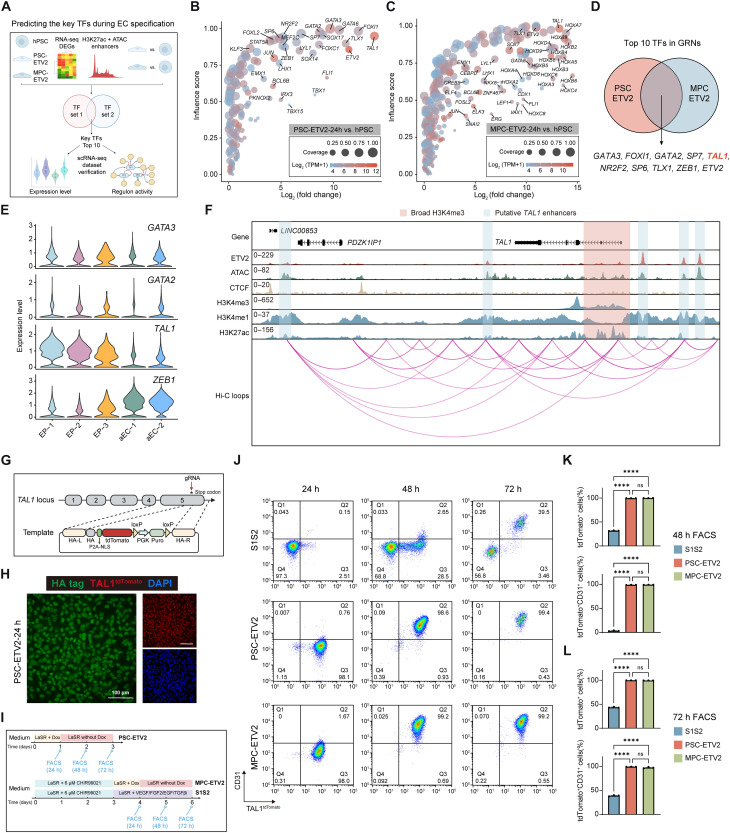
Integrative analysis reveals TAL1 as a potential master regulator of endothelial cell fate determination. (**A**) Schematic representation of integrating multi-omics data to reconstruct Gene Regulatory Networks (GRNs) and identify key transcription factors (TFs). (**B**) Bubble plots displaying the influence score, genomic coverage, and transcript levels of candidate TFs within the GRNs inferred by ANANSE in PSC-ETV2-24h cells. (**C**) The influence score, coverage, and expression level of TFs in MPC-ETV2-24h cells. (**D**) Venn diagram showing the intersection of the top 10 influential TFs between PSC-ETV2-24h and MPC-ETV2-24h GRNs. (**E**) Violin plots showing the expression patterns of *GATA3*, *GATA2*, *TAL1*, and *ZEB1* in different clusters. (**F**) Genome browser views showing the broad H3K4me3 marked *TAL1* promoter, putative *TAL1* enhancers, and the chromatin interaction between putative *TAL1* enhancers and *TAL1* promoter. (**G**) Schematic representation of the knock-in strategy for *TAL1-HA-tdTomato* cell line construction. (**H**) Representative immunofluorescence images of HA tag showing colocalization with tdTomato signal. (**I**) Schematic illustration of three differentiation method and flow cytometric analysis time points. (**J**) Flow cytometric analysis of CD31 expression and tdTomato signaling using the PSC-ETV2 method, the MPC-ETV2 method, or the S1S2 method at three time points. (**K**) Quantification of the ratio of tdTomato^+^ cells and tdTomato^+^CD31^+^ cells at 48 hours (*n* = 3). (**L**) Quantification of the ratio of tdTomato^+^ cells and tdTomato^+^CD31^+^ cells at 72 hours (*n* = 3). Scale bar, 100 μm in (H). All data are presented as mean ± SEM. *n* indicates biological replicates [(K) and (L)]. Statistical significance was assessed by unpaired two-tailed Student’s *t* test [(K) and (L)]. The level of significance was labeled as ns, ^*^, ^**^, ^***^, and ^****^, denoting nonsignificant and *P* value of <0.05, <0.01, <0.001, and <0.0001, respectively.

We next sought to identify the indispensable cofactors that cooperate with ETV2 to execute the human endothelial program. Previous studies have established Brg1 as a critical cofactor for ETV2 during the reprogramming of mouse embryonic fibroblasts (MEFs) into ECs (*16*). Recently, another study identified GABPA and REST as the activator and repressor during the specification of MPCs to ECs (*34*). However, their broad expression patterns across diverse cell types suggest a lack of endothelial lineage specificity (*35*–*37*). To efficiently identify lineage-specific regulators of EC fate, we integrated epigenomic profiles and DEGs to pinpoint master regulators whose expression levels were up-regulated during ETV2 overexpression (Fig. 3A). We used ANANSE (ANalysis Algorithm for Networks Specified by Enhancers) to assess the impact of various TFs in ETV2-primed EC fate commitment using ChIP-seq data for H3K27ac, ATAC-seq, and DEGs from RNA-seq as input (*38*). This unbiased approach identified a cohort of candidate TFs, with TAL1 emerging as a top-ranked regulator in both PSC-derived and MPC-derived endothelial programs (Fig. 3, B and C, and fig. S6, A and B). Because both PSC-ETV2 and MPC-ETV2 methods could drive EC differentiation, we intersected the genes in the GRNs of these two groups for further screening (Fig. 3D). Among the 10 candidates, GATA2, GATA3, and TAL1 were recognized as key regulators of hematopoiesis, hematopoietic stem/progenitor cell (HSPC) differentiation, and HSPC maintenance (*39*–*41*). NR2F2 is a master TF for venous and lymphatic ECs, playing a pivotal role in maintaining venous EC identity by suppressing the effects of ERG (*42*, *43*). Unlike other candidates, TAL1 exhibited exceptionally high expression and restricted regulon activity within EP clusters (Fig. 3E and fig. S6C). In addition, we noticed that the promoter of TAL1 was marked by broad H3K4me3, which was recognized as the feature of cell identity genes (Fig. 3F) (*44*, *45*). Genome browser views of *TAL1* revealed the establishment of putative *TAL1* enhancers around ETV2 binding sites, which showed chromatin accessibility, H3K4me1 and H3K27ac deposition, and enhancer-promoter interactions identified via Hi-C (Fig. 3F). In addition, the two downstream and the nearest upstream putative enhancers were identified as the cell type–specific enhancers of human umbilical vein endothelial cells (HUVECs) and K562 cells (*46*).

To monitor the spatiotemporal dynamics of TAL1 during human EC differentiation, we engineered a *TAL1-HA tdTomato/iETV2* hPSC line (Fig. 3G and fig. S7, A to D). Immunostaining revealed the colocalization of tdTomato with HA tag signaling in PSC-ETV2-24h cells (Fig. 3H). Notably, when comparing the PSC-ETV2 method and MPC-ETV2 method with conventional S1S2-based differentiation, we observed a profound disparity in TAL1 activation kinetics. While the S1S2 method delayed TAL1 onset until 48 hours and achieved only ∼40% tdTomato^+^CD31^+^ efficiency by 72 hours, ETV2 overexpression triggered a swift and synchronized activation of TAL1 within the first 24 hours, resulting in a nearly homogeneous population of tdTomato^+^CD31^+^ ECs (Fig. 3, I to K). This revealed that ETV2 overexpression efficiently activated TAL1 and synergistically generated ECs. To functionally validate the necessity of this rapid activation, we performed CRISPR-Cas9–mediated KO of *TAL1* alongside known EC regulators *FLI1* and *ERG*. Intriguingly, while *FLI1* or *ERG* deficiency had negligible effects on initial EC specification, TAL1-KO almost completely abolished the generation of CD31^+^CD144^+^ cells and disrupted the expression of other core TFs (fig. S6, D to F). As a complementary experiment, we overexpressed TAL1, FLI1, or ERG in hPSCs to assess their respective capacities to promote EC differentiation (fig. S6G). Consistent with a previous study (*47*), FLI1 or ERG overexpression successfully generated CD31^+^CD144^+^ ECs, while TAL1 overexpression alone was insufficient to drive EC differentiation (fig. S6, H to J). These results distinguish TAL1 from other established factors and position it as a potential master cofactor that functions in concert with ETV2 to initiate human endothelial fate.

### TAL1 is indispensable for ETV2-primed EC fate commitment in human cells

The functional requirement for TAL1 during EC fate commitment appears to be species-dependent. In mice, constitutive KO of Tal1 resulted in embryonic lethality at E9.5 due to the absence of yolk sac primitive erythropoiesis and myelopoiesis, highlighting its critical role in hematopoiesis (*48*, *49*). Furthermore, Tal1-KO mESCs were unable to contribute to blood and spleen formation in mouse chimeras (*48*, *50*). Another study using mouse embryonic body differentiation showed that high level of Etv2 turned FLK1^+^ mesoderm cells into Etv2^high^Tal1^+^ cells, indicating that Etv2 could initiate the expression of Tal1 for hematopoiesis (*51*). However, during mouse vasculature development, Tal1-KO embryos still exhibited endothelial-lined blood vessels (*49*). In chimeras, Tal1-KO/Tie2-lacZ tagged ECs did not contribute to large vessels but could be observed in small capillaries (*52*). Our re-analysis of mouse gastrulation scRNA-seq datasets confirmed that while Etv2 and Tal1 are transiently coexpressed in hematoendothelial progenitors, Tal1 deficiency only marginally affects the expression of pan-endothelial markers like *Pecam1* and *Cdh5*, despite a slight reduction in functional genes such as *Nos3*, *Flt1*, *Col4a1*, and *Col4a2* (fig. S8, A to F) (*53*). While murine Tal1 appears to influence arterial identity by modulating Sox17 and Nr2f2 levels, it remains dispensable for the fundamental commitment to the endothelial lineage (fig. S8, G and H).

**Fig. 4.**
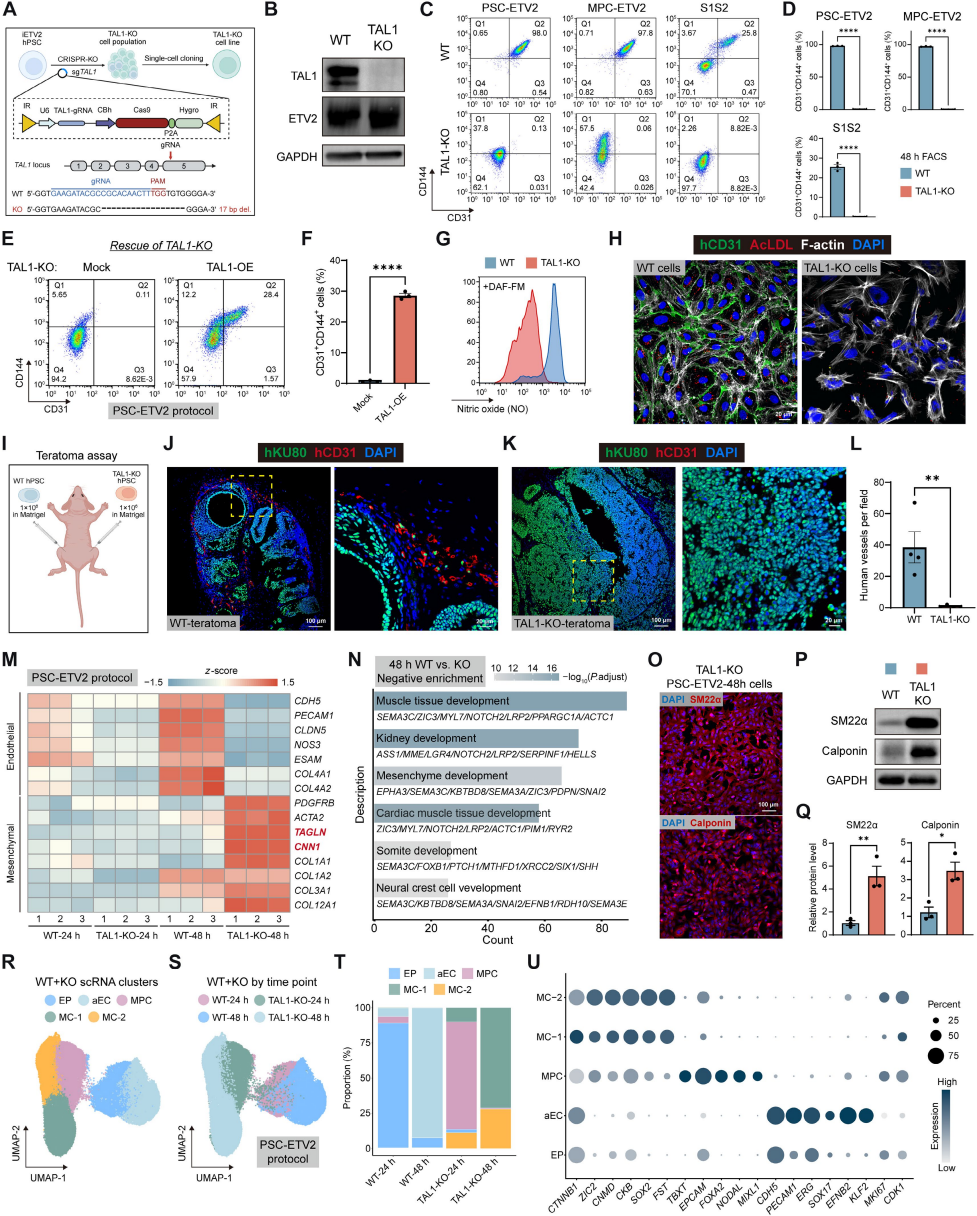
TAL1 is essential for endothelial fate commitment and prevents ectopic mesenchymal fate transition. (**A**) *TAL1* knockout (KO) strategy and Sanger sequencing verification. (**B**) Representative Western blot imaging showing the expression level of TAL1 and ETV2. (**C**) Flow cytometric analysis of cells derived using the PSC-ETV2, MPC-ETV2, or S1S2 method. (**D**) Quantification of the ratio of CD31^+^CD144^+^ cells (*n* = 3). (**E**) Flow cytometric analysis of cells in the rescue experiment. (**F**) Quantification of the ratio of CD31^+^CD144^+^ cells in the rescue experiment. (**G**) Flow cytometric analysis of the nitric oxide production ability. (**H**) Representative immunofluorescence images of AcLDL uptake assay. (**I**) Schematic representation of the teratoma assay. (**J**) Representative immunofluorescence images of WT hPSC-derived teratoma. (**K**) Representative immunofluorescence images of TAL1-KO hPSC-derived teratoma. (**L**) Quantification of the human vessels in teratoma (*n* = 4). (**M**) Heatmap showing the expression of endothelial or mesenchymal marker genes. (**N**) Gene ontology (GO) enrichment analysis of the up-regulated genes in KO-48h cells. (**O**) Representative immunofluorescence images of SM22α and Calponin in TAL1-KO cells. (**P**) Representative Western blot imaging showing the expression level of SM22α and Calponin. (**Q**) Quantification of the protein level of SM22α and Calponin (*n* = 3). (**R**) UMAP visualization of WT and TAL1-KO scRNA-seq data, colored by unsupervised clustering. (**S**) UMAP of cells colored according to samples. (**T**) The proportion of each cell cluster. (**U**) Dot plot showing the expression patterns of marker genes. Scale bar, 20 μm in (H) and in the right panels of (J) and (K) and 100 μm in the left panels of (J) and (K). All data are presented as mean ± SEM. *n* indicates biological replicates [(D), (F), and (Q)] and the number of mice (L). Statistical significance was assessed by unpaired two-tailed Student’s *t* test [(D), (F), (L), and (Q)]. The level of significance was labeled as ns, ^*^, ^**^, ^***^, and ^****^, denoting nonsignificant and *P* value of <0.05, <0.01, <0.001, and <0.0001, respectively.

To assess the requirement of TAL1 in human endothelial commitment, we established an *iETV2*-*3×HA/TAL1-KO* hPSC cell line by integrating a U6-gRNA-CBh-Cas9-Hygro cassette using the Sleeping Beauty transposon system. This cell line allows for the overexpression of ETV2 within a TAL1-KO background. Single clones were isolated and underwent Sanger sequencing. A clone was identified with a 17-bp deletion, causing the frame shift of *TAL1* open reading frame (Fig. 4A). Western blot analysis confirmed the KO of TAL1, while maintaining normal expression level of ETV2 after administration of Dox (Fig. 4B). Across all tested differentiation paradigms including PSC-ETV2, MPC-ETV2, and conventional S1S2 methods, TAL1 deficiency failed to generate CD31^+^CD144^+^ ECs (Fig. 4, C and D). Furthermore, prolonged culture of these cells up to 7 days failed to rescue the phenotype, confirming that TAL1 deficiency indeed perturbs the initiation of EC differentiation rather than simply delaying the process (fig. S9, A to C). Similarly, extending the ETV2 overexpression window to 3 days (3Dox protocol) using the PSC-ETV2 method was also unable to recover the loss of EC generation (fig. S9, D to F). Although using the 3Dox protocol in the MPC-ETV2 method could generate CD31^+^CD144^+^ cells, these cells failed to maintain the expression of CD31 and CD144 once ETV2 overexpression was withdrawn, indicating that the expression of EC genes is ETV2 dependent (fig. S9, D to F). Crucially, this defect was rescued by the exogenous reintroduction of TAL1, which restored the generation of EC population (Fig. 4, E and F). Functional assays further revealed that TAL1-KO cells were devoid of prototypical endothelial characteristics, including nitric oxide production and acetylated LDL (AcLDL) uptake (Fig. 4, G and H). To evaluate this requirement in a complex physiological environment, we performed an in vivo teratoma assay by intramuscular injection of wild type (WT) and TAL1-KO hPSCs (Fig. 4I). While WT-derived teratomas exhibited hCD31^+^hKU80^+^ human ECs, TAL1-KO hPSCs were entirely incapable of generating human endothelial lineages in vivo (Fig. 4, J to L). In addition, staining of NESTIN to mark ectoderm-derived neuroepithelium or progenitor cells, alongside KRT20 to mark endoderm-derived mature epithelium, showed no significant differences between WT-teratomas and TAL1-KO teratomas, indicating that TAL1-KO did not affect the specification toward ectoderm and endoderm lineage (fig. S10, A to E). These results establish TAL1 as a master regulator that is indispensable for the initial specification of human ECs.

To define the molecular basis of the differentiation failure in TAL1-KO cells, we performed temporal RNA-seq profiling at 24 and 48 hours postinduction. PCA revealed that TAL1-KO caused significant differences in the transcriptomes at 24 and 48 hours (fig. S11A). WT groups and KO groups also showed low correlations (fig. S11B). The transcriptome of TAL1-KO-48h cells was even closer to hPSC instead of WT-48h cells and the two groups showed completely different trajectories. Differential gene expression analysis indicated that after TAL1-KO, the expression of EC-related genes was significantly attenuated (e.g., *CDH5*, *PECAM1*, *CLDN5*, *NOS3*, and *ESAM*), and TAL1-KO-48h cells showed expression of MC markers (*TAGLN* and *CNN1*), revealing that TAL1-KO triggered the expression of alternative-lineage specific genes (Fig. 4M). However, a comparison between TAL1-KO cells and hPSCs revealed that ETV2 alone is capable of initiating some degree of EC gene expression, albeit at a substantially lower level than that observed in WT cells (fig. S11C). Functional enrichment analysis further corroborated this fate transition, with up-regulated genes in TAL1-deficient cells being significantly enriched for muscle tissue, renal, and cardiac development programs (Fig. 4N and fig. S11D). To confirm the ectopic expression of alternative lineage-specific genes, we performed immunostaining and Western Blotting on SM22α and Calponin (Fig. 4, O to Q).

We further resolved this lineage redirection at single-cell resolution. Integrated scRNA-seq analysis demonstrated that TAL1-KO cells bypassed the EP stage, instead bifurcating into MPC and MC populations (Fig. 4, R to T, and fig. S11E). The two MC clusters did not express *CDH5* and *PECAM1*, while marked by expression of *CTNNB1*, *ZIC2*, *CNMD*, *CKB*, and *SOX2* (Fig. 4U). Intriguingly, the TAL1-deficient MPCs expressed primitive streak and mesendoderm markers (*TBXT*, *MIXL1*, and *FOXA2*) but remained negative for definitive endoderm (*SOX17*), neuroectoderm (*PAX6*), and neural crest (*TFAP2A*) identities (fig. S11F). Regulon activity analysis also revealed that MPCs and MCs lost the activity of ETV2, TAL1, and ERG, while the MPC enriched for MIXL1, CDX1, and FOXA2, and MCs enriched for ZIC3, SOX2, and SOX11 (fig. S11G). RNA velocity and latent time trajectories confirmed that in the absence of TAL1, the developmental potential of hPSCs is restricted solely to the MC lineage, precluding any transition to the arterial EC fate (fig. S11, H and I). These results establish TAL1 as an essential lineage gatekeeper that is required to stabilize the endothelial program while actively suppressing alternative mesodermal fates.

### TAL1 recruits p300 to activate ETV2-primed endothelial-specific enhancers

**Fig. 5.**
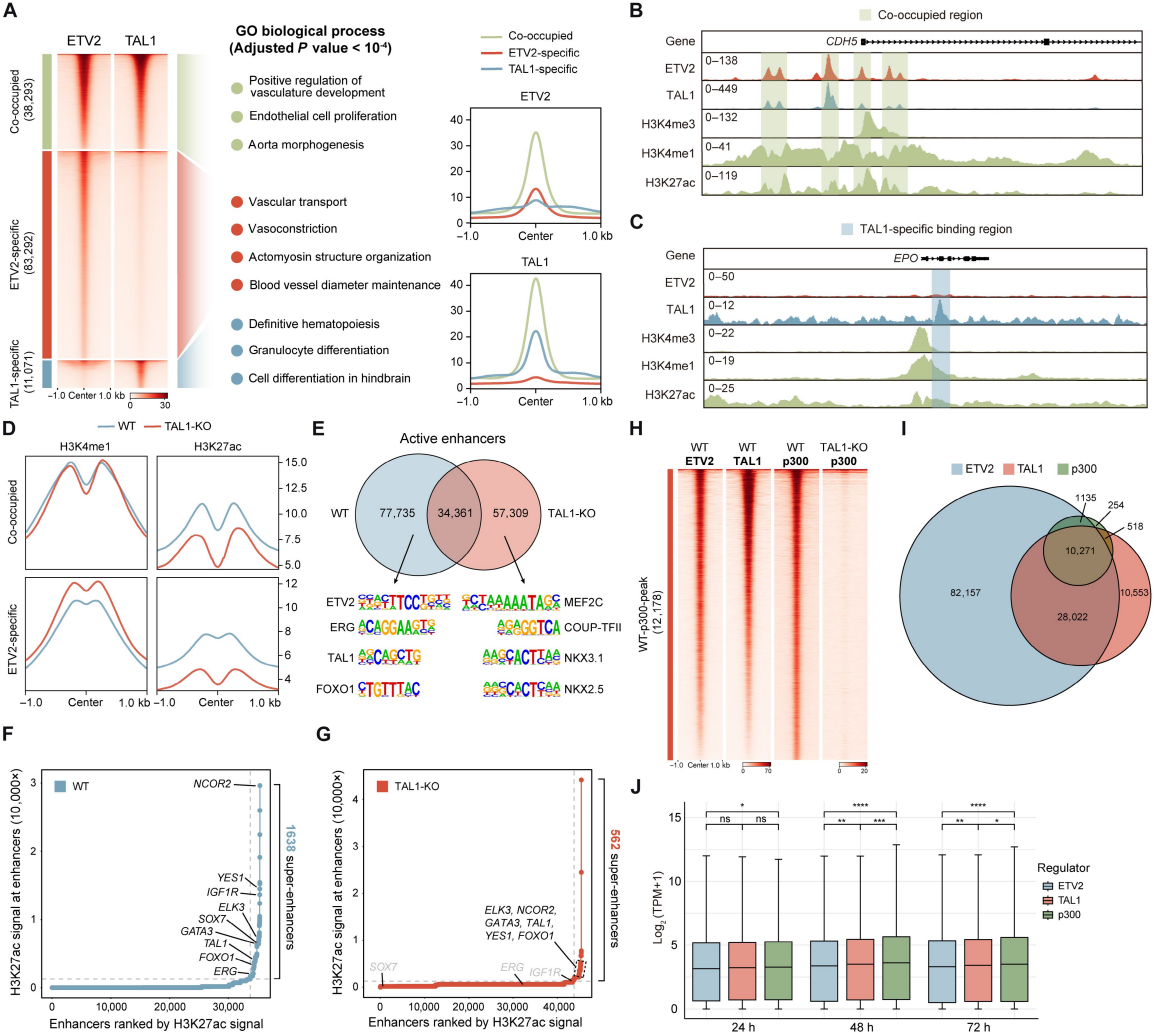
TAL1 recruits p300 to activate ETV2-primed endothelial-specific enhancers. (**A**) Heatmap showing the signal of ETV2 and TAL1 at co-occupied, ETV2-specific, or TAL1-specific regions and the related GO terms. All the displayed terms have adjusted *P* value <10^−4^. Average signal of ETV2 and TAL1 at co-occupied, ETV2-specific, or TAL1-specific regions. (**B**) Genome browser views showing the ETV2 and TAL1 co-occupied region upstream of *CDH5*. (**C**) Genome browser views showing the TAL1-specific binding region at the intron of *EPO*. (**D**) Average signal of H3K4me1 and H3K27ac at ETV2 and TAL1 co-occupied or ETV2-specific bound active enhancers in wild type (WT) and TAL1 knockout (KO) PSC-ETV2-24h cells. (**E**) Venn diagram showing the intersection of active enhancers in WT and TAL1-KO PSC-ETV2-24h cells. Motif enrichment is performed on WT-specific or KO-specific active enhancer regions. (**F**) Super-enhancers identified in WT PSC-ETV2-24h cells according to the intensity of H3K27ac using Rank Ordering of Super Enhancer (ROSE). (**G**) Super-enhancers identified in TAL1-KO PSC-ETV2-24h cells. (**H**) Heatmaps of ETV2, TAL1, and p300 signals in WT and TAL1-KO cells at p300-binding regions identified in WT cells. (**I**) Venn diagram showing the intersection of ETV2, TAL1, and p300 binding regions. (**J**) The expression level of genes regulated by ETV2, TAL1, and p300 in PSC-ETV2-24h, PSC-ETV2-48h, and PSC-ETV2-72h cells. All data are presented as mean ± SEM. Statistical significance was assessed by two-way ANOVA with Tukey’s post hoc test (J). The level of significance was labeled as ns, ^*^, ^**^, ^***^, and ^****^, denoting nonsignificant and *P* value of <0.05, <0.01, <0.001, and <0.0001, respectively.

To decipher the cooperative mechanisms between TAL1 and ETV2, we performed ChIP-seq for HA-tagged TAL1 in PSC-ETV2-24h cells. Then, we clustered different peaks between ETV2 and TAL1. Most of the TAL1-binding sites also had ETV2 occupancy, suggesting potential cooperation between these two TFs (Fig. 5A). After performing peak annotation and GO pathway enrichment, we found that the co-occupied peaks were closely associated with EC differentiation and proliferation. In contrast, ETV2-specific peaks were linked to EC function, while TAL1-specific peaks were associated with alternative cell fates, such as leukocyte and hindbrain neuron differentiation. Notably, ETV2 and TAL1 exhibited stronger binding strength at the co-occupied regions. For instance, ETV2 and TAL1 co-occupied *CDH5* promoter and enhancers, with significant deposition of active promoter and enhancer-related histone markers (Fig. 5B). TAL1 also bound to the intragenic region of *EPO*, but with much weaker strength and with no deposition of histone markers or ETV2 occupancy (Fig. 5C).

To further characterize the specific binding sites of TAL1 in PSC-ETV2-24h cells, we integrated published TAL1 ChIP-seq data from T-ALL cells, ECFCs, and HSPCs (*54*–*56*). We counted the sequencing reads in a 10,000-bp bin and performed PCA. The binding profile of PSC-ETV2-TAL1 closely resembled that of ECFC-TAL1 (fig. S12A). Moreover, PSC-ETV2-TAL1 and ECFC-TAL1 showed high correlations in their binding locations across chromosomes based on the 10,000-bp bins (fig. S12B). Clustering and GO enrichment analysis of the distinct peaks revealed that PSC-ETV2-TAL1–specific peaks were associated with EC fate specification and mesoderm formation, while ECFC-TAL1–specific peaks regulated EC functions, such as response to damage-associated molecular patterns (DAMPs) and secretion of prostaglandins (fig. S12C). One of the differentially bound peaks was located within the gene body of *GATA3*, accompanied by deposition of H3K4me1 and H3K27ac. In addition, the transcript level of *GATA3* was significantly higher in PSC-ETV2-24h cells (fig. S12, D and E), suggesting a divergent transcriptome between PSC-ETV2-24h cells and ECFCs driven by distinct TAL1 binding patterns. Motif enrichment on these different peaks revealed divergence in the motifs of the AP-1 complex and its subunits, suggesting that AP-1 may act as a potential cofactor of ETV2 and TAL1 (fig. S12F). We also observed that PSC-ETV2-24h cells had comparable *JUN* transcript levels compared to ECFC, but the level was drastically decreased at 48 h, indicating that the functional incompetence of PSC-ETV2 ECs may be attributed to the lack of sustained expression of AP-1 components (fig. S12G). Collectively, these findings suggested that TAL1 cooperated with ETV2 on endothelial-specific enhancers coregulating the expression of EC-related genes, and highlighted its critical role in EC fate determination at the epigenetic level.

We next investigated whether TAL1 is required for the deposition of active epigenetic marks. Notably, while H3K4me1 levels remained largely intact, TAL1 deficiency led to a significant reduction in H3K27ac signals across ETV2-bound enhancers (Fig. 5D). TAL1-KO also affected the enhancer landscape, resulting in the loss of 77,735 active enhancers and the gain of 57,309 active enhancers (Fig. 5E). Motif enrichment analysis of these two groups of enhancers revealed that the lost active enhancers were predominantly associated with EC-related TFs (ETV2, ERG, TAL1, and FOXO1), whereas the gained enhancers were more closely linked to venous EC-related TFs and non-EC TFs (MEF2C, COUP-TFII, NKX3.1, and NKX2.5). Super-enhancers (SEs) were large clusters of active enhancers that often drive the expression of cell identity genes (*57*). The establishment of specific SE patterns plays a crucial role in cell fate determination. We next aimed to investigate the impact of TAL1-KO on the formation of EC-specific SEs. Before TAL1-KO, we identified 1638 SEs based on H3K27ac intensity using Rank Ordering of Super Enhancer (ROSE) (Fig. 5F) (*58*, *59*). These SEs regulated the expression of several well-known EC-related genes (e.g., *YES1*, *ELK3*, *SOX7*, *GATA3*, *TAL1*, *FOXO1*, and *ERG*). After TAL1-KO, the number of SEs decreased to 562, and several of these SEs showed reduced H3K27ac intensity (Fig. 5G). Considering the potential cooperation between TAL1 and histone acetyltransferase, we hypothesized that the alteration in H3K27ac following TAL1-KO was due to the failure of H3K27ac writer recruitment to the TAL1-bound enhancers. To investigate this, we profiled the p300 binding sites using ChIP-seq and found that among all the p300 peaks in WT-24h cells, no p300 binding was detected in the KO-24h cells (Fig. 5H). Venn diagram of ETV2, TAL1, and p300 binding sites revealed that most of the p300 binding sites coincided with ETV2 and TAL1 binding, while only 254 peaks did not involve ETV2 or TAL1 (Fig. 5I). We further examined the expression levels of genes regulated by ETV2 (∼110,000 peaks), TAL1 (∼50,000 peaks), and p300 (∼10,000 peaks). We found that genes regulated by TAL1 or p300 showed significantly higher expression at 48 and 72 hours, indicating that these peaks were substantially functional (Fig. 5J). Collectively, these data establish a hierarchical model where TAL1 serves as an indispensable lineage-specific bridge, tethering p300 to ETV2-primed sites to facilitate the formation of the enhancer landscape required for human endothelial fate.

### TAL1 physically interacts with ETV2 to regulate EC fate

**Fig. 6.**
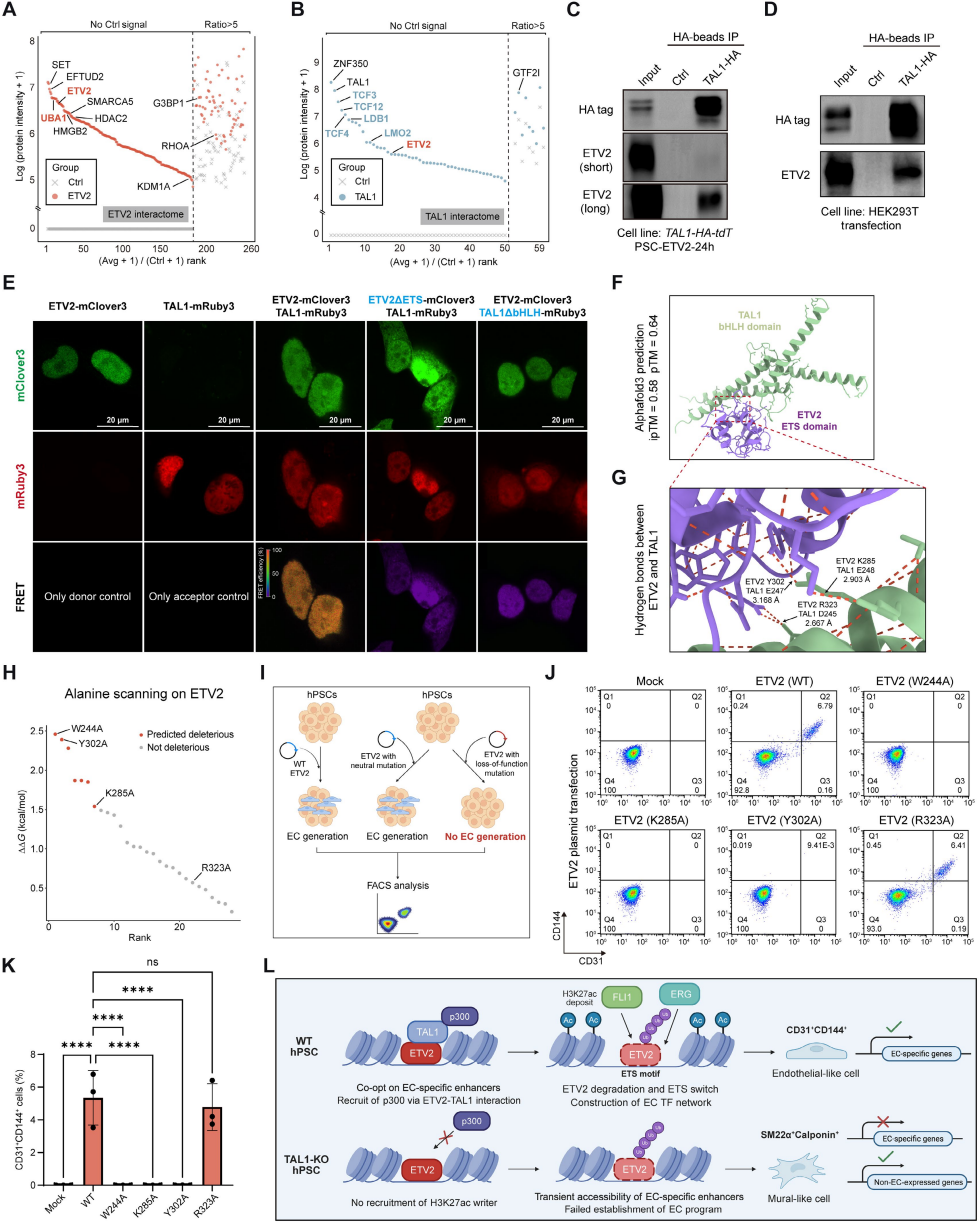
TAL1 physically interacts with ETV2 to regulate endothelial cell fate. (**A**) Dot plot demonstrating the interactome of ETV2 in PSC-ETV2-24h cells identified via immunoprecipitation–mass spectrometry (IP-MS). (**B**) The interactome of TAL1 in PSC-ETV2-24h cells. (**C**) Endogenous co-immunoprecipitation (co-IP) in PSC-ETV2-24h cells, demonstrating the physical association between ETV2 and TAL1. Short and long indicate the exposure times used to optimize the visualization of ETV2 bands in the input and IP lanes, respectively. (**D**) Co-IP analysis of ETV2 and TAL1 in transfected HEK293T cells, confirming the protein-protein interaction in an orthogonal system. (**E**) Forster resonance energy transfer (FRET) analysis using ETV2, TAL1, and ETS or bHLH domain deleted mutants fused with mClover3 and mRuby3, respectively. (**F**) AlphaFold3-based structural prediction of the complex between the ETV2 ETS domain (purple) and the TAL1 bHLH domain (green). (**G**) Detailed structural view highlighting the hydrogen-bonding network at the ETV2-TAL1 interface. (**H**) In silico alanine scanning mutagenesis identifying W244, Y302, and K285 as critical residues on the ETV2 ETS domain required for TAL1 recruitment. (**I**) Schematic of the functional validation experiment using ETV2 wild type (WT) and point-mutant constructs (W244A, Y302A, K285A, and R323A). (**J**) Flow cytometric analysis of cells electroporated with either WT or mutant ETV2 plasmids. (**K**) Quantification of the ratio of CD31^+^CD144^+^ cells (*n* = 3). (**L**) Proposed working model: ETV2 primes the chromatin landscape, while its physical interaction with TAL1 facilitates p300 recruitment to activate endothelial-specific enhancers, collectively driving the endothelial cell fate program. Scale bar, 20 μm in (E). All data are presented as mean ± SEM. Statistical significance was assessed by one-way ANOVA with Tukey’s post hoc test (K). The level of significance was labeled as ns, ^*^, ^**^, ^***^, and ^****^, denoting nonsignificant and *P* value of <0.05, <0.01, <0.001, and <0.0001, respectively.

Given the extensive co-occupancy of TAL1 and ETV2 at endothelial-specific enhancers, we reasoned that these two TFs might function through a direct physical association. To systematically identify their interacting partners, we performed immunoprecipitation followed by mass spectrometry (IP-MS) in PSC-ETV2 cells at 24 hours postinduction using the *iETV2*-*3×HA* and *TAL1-HA-tdTomato/iETV2* cell line with anti-HA tag magnetic beads. The *iETV2* cell line without HA-tagged protein expression served as the IP control. In total, 168 ETV2-specific and 28 TAL1-specific proteins were identified (fig. S13A). These proteins were clustered into two groups: those with signals exclusively in the experimental groups and those with at least a fivefold higher enrichment in the experimental groups. ETV2 interacted with chromatin remodeling proteins (SMARCA5 and HMGB2), histone deacetylase HDAC2, and the ubiquitin-activating enzyme UBA1 (Fig. 6A). TAL1 showed interaction with LDB1 and LMO2, which were components of a previously found tetra-complex (TAL1, LDB1, LMO2, and GATA2), as well as E-box TFs TCF3 and TCF4, which are cofactors of bHLH family TFs (Fig. 6B) (*41*). GO enrichment analysis revealed that ETV2-interacting proteins were involved in chromatin remodeling, consistent with the pioneering role of ETV2 (fig. S13B). TAL1-interacting proteins were associated with E-box binding and bHLH TFs, suggesting cooperation between E-box TFs during EC fate commitment (fig. S13C). Beyond their core roles in transcriptional regulation, the intersection of ETV2 and TAL1 interactomes revealed a robust enrichment of proteins involved in RNA splicing (e.g., SRRM2 and SRSF6). Notably, the corecruitment of β-catenin suggests that the ETV2-TAL1 complex may participate in the regulation of Wnt signaling to drive endothelial fate specification. In addition, the interaction between ETV2 and UBA1 prompted us to examine the stability of ETV2 protein. Consistent with its transient expression pattern, proteasome inhibition with MG132 markedly prolonged ETV2 protein abundance beyond 24 hours (fig. S13D). Moreover, mutagenesis of lysine residues within the ETS domain revealed that five of six substitutions substantially increased ETV2 protein levels, indicating ubiquitin-mediated degradation as a major determinant of its narrow temporal window of action (fig. S13E).

Notably, ETV2 was also detected within the TAL1 interactome, suggesting a direct partnership between the two factors. Co-immunoprecipitation (co-IP) in both PSC-ETV2-24h cells and human embryonic kidney (HEK) 293T cells confirmed a robust and direct physical interaction between the two TFs (Fig. 6, C and D). We next performed Forster resonance energy transfer assay (FRET) assay to further explore the potential direct physical interaction between ETV2 and TAL1. Notably, ETV2 and TAL1 exhibited evident FRET signals when fused with mClover3 and mRuby3, respectively (Fig. 6E). These signals significantly diminished upon the deletion of either the ETS domain of ETV2 or the bHLH domain of TAL1, indicating that the direct physical interaction relies specifically on these two domains. Leveraging AlphaFold3-based multimer prediction (*60*), we identified a high-confidence interface between the ETV2 ETS domain and the TAL1 bHLH domain (ipTM = 0.58) (Fig. 6F and fig. S13, F and G). Detailed structural modeling suggested that this interaction is stabilized by a core hydrogen-bond network specifically involving residues lysine 285 (K285), tyrosine 302 (Y302), and arginine 323 (R323) of ETV2 and a hydrophobic binding pocket formed by TAL1 proline 215 (P215)/tyrosine 235 (Y235)/phenylalanine 238 (F238) that accommodates ETV2 tryptophan 244 (W244) (Fig. 6G and fig. S13H).

To functionally validate this structural interface, we performed in silico alanine scanning followed by in vitro mutagenesis (Fig. 6H). While the arginine 323 to alanine (R323A) mutation (a predicted neutral variation) had negligible effects on endothelial induction, mutations at the core interface [tryptophan 244 to alanine (W244A), lysine 285 to alanine (K285A), tyrosine 302 to alanine (Y302A)] completely abolished the generation of CD31^+^CD144^+^ ECs (Fig. 6, I to K, and fig. S13I). These findings demonstrate that the precise physical docking of ETV2 and TAL1 is indispensable for the initiation of the endothelial program.

Collectively, our study supports a hierarchical model for human endothelial specification (Fig. 6L). In WT hPSCs, ETV2 cooperates with TAL1 to bind endothelial-specific enhancers, where the ETV2-TAL1 complex serves as a scaffold to recruit p300. This recruitment triggers H3K27ac deposition and catalyzes the assembly of a robust endothelial TF network. In the absence of TAL1, ETV2 fails to recruit the H3K27ac writer, leading to a transient and unproductive chromatin opening that results in a fate switch toward mural-like cells.

## DISCUSSION

A central finding of our study is the indispensable role of TAL1 in human endothelial specification, a requirement that notably diverges from murine development. In mice, while Etv2 and Tal1 exhibit transient coexpression in hematoendothelial progenitors, Tal1 remains largely dispensable for the fundamental commitment to the endothelial lineage, with Tal1-deficient embryos still capable of forming endothelial-lined vessels (*53*). Our integrated scRNA-seq and loss-of-function analysis in hPSCs demonstrate that this regulatory logic is evolutionarily rewired in humans. The abolition of endothelial potential upon TAL1-KO positions TAL1 as a mandatory gatekeeper of the human endothelial program. This underscores the necessity of using human-based models to decipher regulatory paradigms that are not fully evolutionarily conserved in murine, particularly when modeling vascular diseases or developing cell-based therapies.

The rapid degradation of ETV2 via the ubiquitin-proteasome pathway aligns with its role as a pioneer factor. Without MG132 treatment, ETV2 protein becomes undetectable within a narrow 6-hour window after Dox withdrawal. This transient pulse necessitates a swift handover of regulatory control to lineage-specific cofactors to stabilize the nascent endothelial identity. Our data support a hierarchical model where ETV2 initiates chromatin opening at distal enhancers, while TAL1 acts as a molecular bridge to recruit the p300 acetyltransferase. Notably, we observed divergent expression kinetics among ETV2 targets; while some genes like *ELK3* respond rapidly, others like *MECOM*, *SOX17*, and *NOTCH4* exhibit delayed activation. This biochemical disparity suggests that while ETV2 primes the epigenetic landscape, the precise temporal execution of the arterial program requires the assembly of a more complex transcriptional apparatus.

Our study reveals that the ETV2-TAL1 axis is required not only for activating the endothelial program but also for actively suppressing alternative mesodermal fates. In the absence of TAL1, hPSCs undergo a fate switch characterized by the collapse of the endothelial regulon and the ectopic activation of MC and mesendoderm programs. Mechanistically, we demonstrate that TAL1 is the primary determinant for SE assembly. The marked contraction of the SE repertoire in TAL1-KO cells reveals that ETV2 alone is insufficient to sustain the high-level H3K27ac deposition required to stabilize cell identity. The failure to recruit p300 to ETV2-bound sites leads to an unsupportive chromatin state, rendering the cells susceptible to fate hijacking by alternative TFs. This gatekeeping function of TAL1 provides a molecular explanation for the high purity and synchrony of ETV2-mediated programming compared to conventional directed differentiation methods.

Our study further demonstrated that TAL1 could physically associate with ETV2. By integrating AlphaFold3-based structure prediction with alanine scanning, we identified the ETS-bHLH docking interface as the structural nexus of the ETV2-TAL1 interaction. The complete loss of vasculogenic potential in ETV2 mutants (W244A, K285A, and Y302A) confirms that physical occupancy of ETV2 is not sufficient to induce endothelial program. While our modeling provides a high-confidence prediction of the direct physical interface between ETV2 and TAL1, it is important to acknowledge that the binding of genomic DNA or the assembly of the larger complex involving GATA2, LMO2, and LDB1 could further modulate the local conformation of these domains.

Collectively, our findings redefine the regulatory architecture of human endothelial specification by establishing TAL1 as an obligate partner of the pioneer factor ETV2 and a molecular gatekeeper of endothelial identity. This TAL1-centered rewiring of the endothelial gene regulatory network highlights a fundamental evolutionary divergence between human and murine vascular development. Our work provides a mechanistic framework in which transient chromatin priming by ETV2 must be rapidly consolidated through TAL1-dependent enhancer activation to achieve stable lineage commitment. This principle where pioneer activity alone is insufficient without timely engagement of lineage-restricted cofactors may represent a general paradigm for human cell fate control. From a translational perspective, delineating the structural and epigenetic basis of the ETV2-TAL1 axis offers a rational blueprint for improving endothelial programming strategies and for engineering vascular cells with higher fidelity and robustness, thereby advancing the development of human-relevant disease models and regenerative therapies.

## MATERIALS AND METHODS

### Animals

Immunodeficient male BALB/c nude mice aged 8 weeks were purchased from the Department of Laboratory Animal Science, Peking University Health Science Center. Mice were housed at 22° ± 2°C, 40 to 60% humidity under standard laboratory conditions (12-hour light:12-hour dark cycle) with free access to rodent feed and water. All animal protocols were approved by the Animal Care and Use Review Committee of Peking University Health Science Center (BCJI0257) in accordance with the *Guide for the Care and Use of Laboratory Animals* published by the US National Institutes of Health.

### Cell culture

The H1 hPSC cell line (WiCell, WA01) was maintained in eTeSR medium (STEMCELL Technologies, 100-1215) on Matrigel (Corning, 354277) at 37°C with 5% CO_2_. Cells were passaged at a ratio of 1:6 using EDTA (Procell, PB180320) when the confluency reached 70 to 90% and seeded with extra 5 μM Y27632 (Topscience, T1870) for the first 24 hours. Cells were cryopreserved using eTeSR with 5 μM Y27632 and 10% dimethyl sulfoxide (DMSO). Cells were routinely checked to avoid potential mycoplasma contamination.

### Generation of the *iETV2*-*3×HA* cell line

The ETV2 coding sequence was PCR amplified and inserted into an all-in-one piggyBac dox-inducible backbone via Gibson Cloning. Three micrograms of piggyBac donor plasmid (Addgene, 251542) and 1 μg of plasmid expressing piggyBac transposase were electroporated into H1 hPSCs using Lonza Nucleofector 2b (Program: B-016). Drug selection was performed with puromycin (5 μg/ml; InvivoGen, ant-pr-1) for 24 hours. Then, puromycin (10 μg/ml) was added for 24 hours to further enrich edited cells. About 3000 cells were plated in a 10-cm dish for clone picking, and the isolated clones were tested for differentiation.

### Generation of the *TAL1-HA-tdTomato* cell line

To construct the knock-in donor plasmid, genomic DNA from H1 hPSC was extracted and used as the template for PCR amplification of the homology arm. The left and right homology arms were first amplified as a single fragment using the primer pair TAL1-F/R and then separately reamplified using the primer pairs TAL1-HAL-F/R and TAL1-HAR-F/R. These fragments were cloned into a plasmid backbone containing a floxed PGK-Puro cassette and an HA-P2A-tdTomato sequence. To construct the Cas9-sgRNA expression plasmid, the PX459 plasmid backbone (Addgene: no. 62988) was digested with Bbs I, and the annealed sgRNA duplex targeting the *TAL1* stop codon was cloned into the digested vector using T4 DNA ligase. Two micrograms of donor plasmid and 1 μg of plasmid expressing Cas9-sgRNA were electroporated into H1 hPSCs as described above. Puromycin (1 μg/ml) was added to enrich edited cells. Clones were isolated as described above and genotyped via junction PCR using primer pair TAL1-G-F/R. All primer sequences are included in table S1.

### Generation of the *iETV2*-*3×HA/TAL1-KO* cell line

To construct the SB-Cas9 KO plasmid expressing Cas9 and sgRNA targeting *TAL1* coding sequence, the coding sequence of Cas9 from *Streptococcus pyogenes* (SpCas9) and P2A-Hygro were amplified and cloned into a Sleeping Beauty transposon backbone containing CBh promoter and U6-gRNA construct. Then, the sgRNA duplex was further cloned into this plasmid. Sleeping Beauty donor plasmid (3 μg) and 1 μg of plasmid expressing Sleeping Beauty transposase (SB100X) were electroporated into the *iETV2*-*3×HA* cell line as described above. Hygromycin (200 μg/ml; InvivoGen, ant-hg-1) was added to enrich the integrated cells. Clones were isolated and genotyped via PCR and Sanger sequencing.

### Differentiation of *iETV2*-*3×HA* cell line to ECs

Cells were passaged as 1:6 for differentiation. For the PSC-ETV2 method, on day 1, medium was changed to Advanced DMEM/F12 (Thermo Fisher Scientific, 12634010) supplemented with Vitamin C (60 μg/ml; Sigma-Aldrich, A8960) and 1× GlutaMAX (Thermo Fisher Scientific, 35050061) (LaSR basal medium) with doxycycline (0.5 μg/ml; Topscience, T1687). Then, medium was changed to LaSR without doxycycline on day 2. For the MPC-ETV2 method, on days 1 to 3, medium was daily changed to LaSR with 6 μM CHIR99021 (Topscience, T2310). At the end of day 3, the differentiated MPCs were dissociated using TrypLE Express (Thermo Fisher Scientific, 12604021) and passaged as 1:6 in LaSR with doxycycline (0.5 μg/ml). Then, medium was changed to LaSR without doxycycline at the end of day 4.

### Differentiation of hPSCs to ECs using the S1S2 method

Cells were passaged as 1:6 for differentiation and MPCs were generated as described above. These MPCs were then replated at 50,000 cells/cm^2^ and exposed to LaSR supplemented with FGF2 (50 ng/ml; SinoBiological, 10014-HNAE), VEGFA (50 ng/ml; SinoBiological, 11066-HNAH), EGF (10 ng/ml; SinoBiological, 10605-HNAE), and 10 μM SB431542 (Topscience, T1726) for 72 hours. Medium was changed daily.

### Flow cytometry

Cells were dissociated into single cells using TrypLE Express and washed once with 1 ml of phosphate-buffered saline (PBS). Then, the cells were resuspended in 100 μl of FACS buffer [PBS with 0.5% bovine serum albumin (BSA) and 0.2 mM EDTA] for antibodies staining at 4°C for 10 min. Cells were further washed three times with 1 ml of FACS buffer and analyzed using Guava easyCyte flow cytometer (Millipore Corporation, Billerica, MA) and FlowJo software (Tree Star Inc., Ashland, OR). The antibodies used for FACS include CD31-FITC (1:100, BioLegend, 303104), CD144-APC (1:100, eBioscience, 17-1449-42), and CD31-APC (1:100, BioLegend, 303116).

### RNA isolation and qPCR analysis

Total RNA was isolated using FastPure Cell/Tissue Total RNA Isolation Kit V2 (Vazyme, RC112-01) following the manufacturer’s protocol. Purified total RNA was reverse transcribed using the HiScript II 1st Strand cDNA Synthesis Kit (Vazyme, R211-01) following the manufacturer’s protocol. qPCR was performed using Taq Pro Universal SYBR qPCR Master Mix (Vazyme, Q712-02), and detection was achieved using Archimed X4 (RocGene). Expression of target genes was normalized to glyceraldehyde-3-phosphate dehydrogenase (GAPDH). All primer sequences for qPCR are included in table S1.

### Immunofluorescence

Cells were washed with PBS, fixed with 4% paraformaldehyde (PFA) for 15 min at room temperature (RT), and permeabilized with 0.1% Triton-X 100 for 10 min. Then, cells were blocked in blocking buffer (PBS with 2% BSA) for 30 min and incubated with primary antibodies diluted in blocking buffer for 30 min at RT. Cells were then washed with PBS for three times and incubated with secondary antibodies diluted in blocking buffer as 1:500 for 15 min at RT. After washing three times with PBS, cells were counterstained with 4′,6-diamidino-2-phenylindole (DAPI, 0.5 μg/ml) for 10 min. Images were captured using Olympus CKX53 or Nikon AXR. The primary antibodies used for immunofluorescence include ETV2 (Rabbit, 1:500, Abcam, ab181847), HA tag (Mouse, 1:200, Abclonal, AE008), OCT4 (Mouse, 1:200, Santa Cruz, sc-5279), NANOG (Rabbit, 1:800, Cell Signaling Technology, 3580), CD31 (Sheep, 1:200, R&D Systems, AF806), vWF (Mouse, 1:200, Santa Cruz, sc-53466), SOX17 (Mouse, 1:200, BD Bioscience, 561590), NOTCH4 (Rabbit, 1:200, Abcam, ab166605), TAL1 (Rabbit, 1:200, Abcam, ab155195), SM22α (Rabbit, 1:800, Abcam, ab14106), and Calponin (Mouse, 1:200, Santa Cruz, sc-58707). For staining of F-actin, the blocked cells were incubated with Actin-TrackerDeepRed-633 (Beyotime, C2241) for 30 min at RT and counterstained with DAPI.

### Western blot

Total proteins were extracted from cells with radioimmunoprecipitation assay (RIPA) lysis buffer (Beyotime, P0013B) supplemented with protease inhibitor and phosphatase inhibitor (Beyotime, P1045). The lysates were centrifuged for 10 min at 12,000*g* at 4°C, and the supernatants were transferred to a new tube. Protein concentration was quantified by bicinchoninic acid (BCA) assay (Beyotime, P0012), then boiled with SDS loading buffer (Beyotime, P0015) and separated on 10% SDS–polyacrylamide electrophoresis gels. Then, proteins were transferred onto a polyvinylidene difluoride membrane. Membrane was blocked in blocking buffer (tris-buffered saline with 0.1% Tween 20 and 5% BSA) at RT for 1 hour and subsequently incubated with primary antibodies diluted in blocking buffer at 4°C overnight. Membrane was washed in tris-buffered saline with 0.1% Tween 20 (0.1% TBST) three times and incubated with horseradish peroxidase (HRP)–conjugated secondary antibodies diluted in blocking buffer (goat anti-mouse, Thermo Fisher Scientific, C31430100, 1:10,000; goat anti-rabbit, Thermo Fisher Scientific, C31460100, 1:5000) at RT for 1 hour. After washing three times in 0.1% TBST, membrane was subjected to BeyoECL Plus reagents (Beyotime, P0018S) to generate chemiluminescent signal. Quantification was performed using ImageJ. The primary antibodies used for Western blot include ETV2 (Rabbit, 1:1000, Abcam, ab181847), TAL1 (Rabbit, 1:1000, Abcam, ab155195), FLI1 (Rabbit, 1:1000, Abcam, ab133485), ERG (Mouse, 1:1000, Santa Cruz, sc-376293), CD144 (Mouse, 1:1000, Santa Cruz, sc-9989), HA tag (Mouse, 1:1000, Abclonal, AE008), GAPDH (Rabbit, 1:20000, Abclonal, A19056), SM22α (Rabbit, 1:1000, Abcam, ab14106), and Calponin (Mouse, 1:1000, Santa Cruz, sc-58707).

### Teratoma formation assay

To evaluate the developmental potential of TAL1-KO hPSCs in vivo, the teratoma formation assay was performed. Briefly, hPSCs were harvested at 80% confluence using EDTA to obtain a single-cell or small-cluster suspension. Approximately 1 × 10^6^ cells were resuspended in 100 μl of hydrogel prepared by mixing type I collagen (Sigma-Aldrich, 5074) and growth factor–reduced Matrigel (Corning, 354230) at a 3:1 (v/v) ratio. The suspension was then injected intramuscularly into the femoral quadriceps of the hindlimb of BALB/c nude mice. For each mouse, WT hPSCs were injected into the left femoral muscle as an internal control, while TAL1-KO hPSCs were injected into the right side. After 4 weeks, the animals were euthanized. The harvested teratomas were fixed in 4% PFA.

### Immunofluorescence on tissue sections

The fixed samples were embedded in paraffin and cut into 4-μm-thick sections. The sections were deparaffinized by incubating in xylene and antigen retrieval was conducted using tris-EDTA buffer (pH 9.0). Then, the sections were blocked using 5% horse serum and incubated with primary antibodies diluted in horse serum overnight at 4°C. The sections were washed three times with PBS and incubated with secondary antibodies diluted in horse serum as 1:500 for 1 hour at RT. After washing three times with PBS, the sections were counterstained with DAPI. Images were captured using Nikon AXR. The primary antibodies used here include CD31 (Sheep, 1:200, R&D Systems, AF806), KU80 (Rabbit, 1:200, Cell Signaling Technology, 2180), MyHC (Mouse, 1:50. DSHB, MF20), and NESTIN (Mouse, 1:200, Santa Cruz, sc-23927). For costaining of hKU80 and KRT20 (Rabbit, 1:200, Cell Signaling Technology, 13063), deparaffinized sections underwent heat-induced antigen retrieval followed by endogenous peroxidase blocking with 3% H_2_O_2_. After blocking with 5% horse serum, sections were incubated with primary antibodies diluted in horse serum overnight at 4°C. Following three PBS washes, slides were incubated with HRP-conjugated secondary antibodies (1:500) for 1 hour at RT. The fluorescent signal was developed using iF488-tyramide (ServiceBio, G1231) in the presence of 0.3% H_2_O_2_ for 10 min. To perform multiplex staining, a second round of antigen retrieval was conducted to strip the antibodies, followed by the same procedure using iF594-tyramide (ServiceBio, G1242) for the subsequent target.

### KO of *TAL1*, *FLI1*, and *ERG*

The sgRNA sequence targeting *TAL1*, *FLI1*, or *ERG* was cloned into the SB-Cas9 KO plasmid. Sleeping Beauty donor plasmid (3 μg) and 1 μg of plasmid expressing SB100X were electroporated into the *iETV2*-*3×HA* cell line as described above. Hygromycin (200 μg/ml; InvivoGen, ant-hg-1) was added to enrich the integrated cells. The sgRNA sequences are included in table S2.

### Overexpression of *TAL1*, *FLI1*, and *ERG*

The coding sequences of *TAL1*, *FLI1*, and *ERG* were PCR amplified from cDNA of PSC-ETV2-24h cells and inserted into an all-in-one piggyBac dox-inducible backbone containing an IRES-mCherry cassette via Gibson Cloning. Three micrograms of piggyBac donor plasmid and 1 μg of plasmid expressing piggyBac transposase were electroporated into H1 hPSCs as described above. Drug selection was performed with puromycin (5 μg/ml) for 24 hours. Then, puromycin (10 μg/ml) was added for 24 hours to further enrich edited cells. To induce transgene expression, doxycycline (0.5 μg/ml) was added to the LaSR basal medium for three consecutive days once cell confluency reached approximately 20%. The efficiency of endothelial differentiation was subsequently evaluated by flow cytometric analysis of CD31 and CD144.

### Rescue of TAL1-KO

Three micrograms of CAG-pA plasmid backbone or CAG-TAL1 plasmid was electroporated into the *iETV2*-*3×HA/TAL1-KO* cell line for transient expression of TAL1. Following electroporation, cells were seeded in eTeSR medium supplemented with 5 μM Y27632 for the first 24 hours. The medium was replaced with LaSR with doxycycline (0.5 μg/ml) for the next 24 hours followed by an additional 24 hours in LaSR without doxycycline. Cells were dissociated into single cells for FACS analysis.

### AcLDL uptake assay

Cells were washed twice with DMEM/F12 and incubated at 37°C for 4 hours with Human DiI-Acetylated Low Density Lipoprotein (5 μg/ml; Yeasen, 20606ES) at 37°C for 4 hours. Following incubation, the supernatant was removed, and the cells were washed three times with DMEM/F12 to remove the excess stain. Then, the cells were fixed with 4% PFA for further staining.

### Nitric oxide production assay

To measure the ability to produce nitric oxide, intracellular nitric oxide levels were quantified using the fluorescent probe DAF-FM diacetate (Beyotime, S0020S). Briefly, the culture medium was replaced with fresh medium supplemented with 1 μM DAF-FM diacetate, and the cells were incubated at 37°C for 30 min. After incubation, the cells were washed to remove extracellular probes. Cells were then harvested for FACS analysis.

### Verification on the degradation of ETV2

Cells were passaged as 1:6 for differentiation. For the first 24 hours, medium was changed to LaSR with doxycycline (0.5 μg/ml). Then, medium was changed to LaSR without doxycycline. MG132 (10 μM; Topscience, T2154) or an equal volume of DMSO was added to the medium. Cells were collected after an extra 6 hours for Western blot analysis. To investigate whether lysine mutations within the ETS domain of ETV2 affect its degradation rate, we generated six ETV2 mutant construct by substituting six lysine residues (K275, K285, K287, K294, K310, and K315) with arginine via site-directed PCR mutagenesis on the iETV2-3×HA plasmid. These mutant or WT plasmids were then electroporated into hPSCs and selected with puromycin (10 μg/ml). Subsequently, the engineered hPSCs were subjected to the PSC-ETV2 differentiation protocol. To assess the protein stability and degradation kinetics of ETV2, total protein was harvested at 24 and 30 hours.

### FRET assay

To investigate the physical interaction between TAL1 and ETV2, the coding sequences were cloned into fluorescent reporter vectors to generate C-terminal fusions with mClover3 (donor) or mRuby3 (acceptor), respectively. Domain-truncated mutants, including ETS domain deletion and bHLH domain deletion, were generated using PCR and Gibson Assembly. For transfection of HEK293T cells, 9 μg of polyethylenimine (PEI) (Yeason, 40815ES) was diluted in 125 μl of Opti-MEM (Thermo Fisher Scientific, 11058021). Three micrograms of donor and acceptor plasmids was diluted in 125 μl of Opti-MEM. The PEI solution was added to the plasmid solution, mixed by vortexing, and incubated for 30 min at RT to allow complex formation. The resulting DNA/PEI complexes were added dropwise to one well of a 12-well plate and incubated for 24 hours. At 48 hours posttransfection, cells were imaged using a Nikon AXR confocal microscope. The sensitized emission method was used to measure FRET efficiency. To calibrate the system and correct for spectral bleed-through and cross-excitation, cells expressing only the donor or only the acceptor were used as controls. FRET efficiency was calculated and processed using NIS-Elements Advanced Research software (Nikon).

### Structure prediction using AlphaFold3

To further investigate the molecular interaction between ETV2 and TAL1, in silico structural prediction was performed using the AlphaFold3. Given that the DNA-binding domains are the most likely interfaces for coregulatory TFs, the amino acid sequences of the ETV2 ETS domain and the TAL1 bHLH domain were used as input for the complex prediction. The modeling was conducted through the AlphaFold Server, using the default parameters for multimer prediction. The resulting structural models were evaluated based on the Predicted Alignment Error matrices to ensure the reliability of the predicted binding interface. The highest-scoring model was visualized and analyzed using ChimeraX to identify specific residue-residue contacts and potential interface.

### Alanine scanning

To identify the key residues contributing to the stability of the ETV2-TAL1 complex, computational alanine scanning was performed using the MutaBind2 server (*61*). The high-resolution structural model of the ETV2 ETS domain in complex with the TAL1 bHLH domain served as the input. Briefly, each interfacial residue identified at the ETV2-TAL1 boundary was systematically mutated to alanine. The binding affinity change ΔΔ*G* upon mutation was calculated to evaluate the energetic contribution of individual side chains. A mutation was considered to significantly disrupt the interaction if the predicted ΔΔ*G* was greater than 1.5 kcal/mol.

### Validation of predicted residues

To experimentally validate the key residues identified by AlphaFold3 and MutaBind2, site-directed mutagenesis was performed using PCR-based methods to introduce specific mutations (W244A, K285A, Y302A, and R323A) into the ETV2 coding sequence. These mutant constructs along with the WT control were cloned into the CAG-pA expression vector. To further evaluate whether these mutations compromised the function of ETV2 during endothelial commitment, 3 μg of each plasmid was electroporated into hPSCs. Cells were seeded in eTeSR medium supplemented with 5 μM Y27632 for the initial 24 hours. The medium was subsequently replaced with LaSR medium for another 48 hours. Last, cells were dissociated into single-cell suspensions and subjected to FACS analysis.

### RNA sequencing

Total RNA was extracted as described above. Libraries were prepared and sequenced by Annoroad Gene Technology (Beijing, China). Briefly, 1 μg of total RNA was used for library preparation. The poly(A) mRNA was isolated using Oligo(dT) beads. The mRNA fragmentation was performed using divalent cations and high temperature. Priming was performed using random primers. First-strand cDNA and second-strand cDNA were synthesized. The purified double-stranded cDNA was then treated to repair both ends and add a dA-tailing in one reaction, followed by a T-A ligation to add adaptors to both ends. Size selection of adaptor-ligated DNA was then performed using DNA Clean Beads. Each sample was then amplified by PCR using P5 and P7 primers, and the PCR products were validated. Then, libraries with different indices were multiplexed and loaded on an Illumina HiSeq instrument for sequencing using a 2 × 150 paired-end configuration. For each group, three biological replicates were conducted.

### Chromatin immunoprecipitation sequencing

ChIP-seq was performed as described previously (*62*). ChIP assays for ETV2, TAL1, p300, and CTCF were performed with 5 × 10^6^ to 10 × 10^6^ cells using antibody against HA tag (1:100, Cell Signaling Technology, 3724), p300 (1:100, Abcam, ab14984), and CTCF (1:250, Sigma-Aldrich, 07-729). ChIP assays for histone markers were performed with 1 × 10^6^ cells using antibodies against H3K4me1 (1:250, Abcam, ab8895), H3K4me3 (1:250, Sigma-Aldrich, 04-745), H3K27ac (1:250, Abcam, ab4729), and H3K27me3 (1:250, Sigma-Aldrich, 07-449). Briefly, cells were cross-linked with 1% formaldehyde at RT for 5 min and quenched with 125 mM glycine at RT for 5 min. Then, the cross-linked chromatin was sonicated in RIPA 0 buffer (10 mM tris-HCl, 1 mM EDTA, 0.1% sodium deoxycholate, 0.1% SDS, and 1% Triton X-100, pH 8.0) supplemented with 0.25% Sarkosyl, 1 mM dithiothreitol, and 1× protease inhibitor to 200 to 500 bp. NaCl (final concentration, 300 mM) was added to the chromatin and antibody mixture before incubation overnight at 4°C. Protein G Dynabeads (30 μl; Thermo Fisher Scientific, 10004D) were added to the mixture and rotated at 4°C for 3 to 6 hours for pulldown. Dynabeads were washed twice with 1 ml of RIPA 0 buffer, twice with 1 ml of RIPA 0 buffer with 0.3 M NaCl, twice with 1 ml of LiCl buffer (250 mM LiCl, 0.5% NP-40, 0.5% sodium deoxycholate, 1 mM EDTA, and 10 mM tris-HCl, pH 8.0), and twice with 1 ml of TE buffer (10 mM tris-HCl and 1 mM EDTA, pH 8.0). Chromatin was eluted in 100 μl of SDS elution buffer (1% SDS, 10 mM EDTA, and 50 mM tris-HCl, pH 8.0) followed by reverse cross-linking at 65°C overnight. The ChIP DNA was treated with RNase A and protease K at 37°C for 30 min and 2 hours, and purified via phenol-chloroform extraction for library preparation. Libraries were prepared and sequenced by Annoroad Gene Technology (Beijing, China). ChIP product was treated with End Prep Enzyme Mix for end repairing, 5′ phosphorylation, and dA-tailing in one reaction, followed by ligation to adaptors with a “T” base overhang. Adaptor-ligated DNA was then recovered using DNA clean beads. Each sample was then amplified by PCR using P5 and P7 primers. The PCR products were cleaned up using DNA clean beads, validated using an Agilent 2100 Bioanalyzer (Agilent Technologies, Palo Alto, CA, USA), and quantified by a Qubit 3.0 Fluorometer (Invitrogen, Carlsbad, CA, USA). Then, libraries with different indices were multiplexed and loaded on an Illumina HiSeq instrument for sequencing using a 2 × 150 paired-end configuration. For H3K4me1, H3K4me3, H3K27ac, and ETV2, three biological replicates were conducted. For H3K27me3, TAL1, p300, and CTCF, two biological replicates were conducted.

### ATAC-seq

Cells were cross-linked with 1% formaldehyde at RT for 5 min and quenched with 125 mM glycine at RT for 5 min. Cell nuclei were extracted with lysis buffer (10 mM tris-HCl, 10 mM NaCl, 3 mM MgCl_2_, and 0.1% IGEPAL CA-630). Tagmentation was performed with Tn5 transposase at 37°C for 30 min followed by reverse cross-linking at 65°C overnight. The library was prepared and sequenced as described above. For each group, three biological replicates were conducted.

### IP-MS

Total proteins were extracted from cells with IP lysis buffer (50 mM tris-HCl, 150 mM NaCl, 0.3% NP-40, and 2 mM EDTA) supplemented with protease inhibitor and phosphatase inhibitor. The lysates were centrifuged for 10 min at 12,000*g* at 4°C, and the supernatants were transferred to a new tube. Protein concentration was quantified by BCA assay. One milligram of total protein of *iETV2*, *iETV2*-*3×HA*, or *TAL1-HA-tdTomato/iETV2* cell line–derived PSC-ETV2-24h cells was used for IP. The total volume of the protein was adjusted to 1 ml using IP lysis buffer and mixed with Pierce Anti-HA Magnetic Beads (Thermo Fisher Scientific, 88836) prewashed with 0.05% TBST. The mixture was incubated at RT for 30 min with mixing. The beads were collected and washed three times with 0.05% TBST and once with ultrapure water. The protein was eluted in 100 μl of 0.1 M glycine, pH 2.0, and neutralized with 15 μl of 1 M tris, pH 8.5. Samples were boiled with SDS loading buffer, separated on 10% gels and stained with Coomassie brilliant blue (Epizyme, PS111). The three IP lanes were excised individually and submitted for MS analysis at Novogene (Beijing, China). For each group, two biological replicates were conducted.

### Hi-C

Briefly, about 5 × 10^6^ cells were cross-linked, neutralized, and lysed. The chromatin was digested with 200 U Dpn II restriction enzyme (Qiagen, 59971) at 37°C. DNA ends were labeled with biotin and incubated at 37°C for 45 min, and the enzyme was inactivated at high temperature. DNA ligation was performed by adding T4 DNA ligase and incubating for 1 to 2 hours. After ligation, proteinase K was added to reverse cross-linking during incubation at 65°C for 1 to 2 hours. DNA fragments were purified and dissolved in water. Unligated ends were then removed. Purified DNA was fragmented to a size of 350 to 500 bp, and DNA ends were then repaired. DNA fragments labeled by biotin were isolated using Dynabeads M-280 Streptavidin (Thermo Fisher Scientific, 60210). The library was prepared and sequenced as described above.

### Single-cell RNA-seq

Cells were dissociated into single cells, filtered through a 40-μm cell strainer and resuspended in RPMI1640 supplemented with 2% FBS. Cell concentration and viability were determined by acridine orange/propidium iodide (AO/PI) dual-fluorescence staining using LUNA-FL Automated Cell Counter (Logos Biosystems). Approximately 17,000 cells were loaded into the 10x Genomics Chromium Controller to target a recovery of 10,000 cells. Single-cell Gel Bead-in-Emulsions and sequencing libraries were generated using the Chromium Next GEM Single Cell 3′ Reagent Kits v3.1 according to the manufacturer’s instructions. Sequencing was performed as described above.

### Single-cell ATAC-seq

Cells were dissociated into single cells, filtered through a 40-μm cell strainer, and resuspended in RPMI1640 supplemented with 2% FBS. Cell concentration and viability were determined by AO/PI staining. Nuclei isolation was performed following the protocol from 10x Genomics. Single-cell ATAC-seq libraries were using the 10x Genomics Chromium Single Cell ATAC Kit v2 (PN-1000390) following the manufacturer’s protocol. Approximately 8000 nuclei were loaded into a Chromium Controller. Sequencing was performed as described above.

### RNA-seq data analysis

Quality control was performed using FastQC (v0.12.1). The reads were trimmed using TrimGalore (v0.6.10) to remove detected adapters. Then, the reads were aligned to hg38 reference human genome with STAR (v2.7.11a) (*63*). The expression count matrix of each sample was calculated using FeatureCounts (v2.0.6) (*64*). The count matrix was normalized using DESeq2 (v1.44.0) for differential expression analyses (*65*). GO analysis and GSEA were performed with R package clusterProfiler (v4.12.6) and enrichplot (v1.24.4) (*66*). Heatmaps were generated using pheatmap (v1.0.12) and ComplexHeatmap (v2.20.0). The RNA-seq datasets of hPSC and ECFC used for comparison were from SRA: PRJNA509218 (*25*).

### ChIP-seq and ATAC-seq data analysis

Quality control and adapter trimming were performed as described above. Then, the clean reads were aligned to the hg19 reference human genome using Bowtie2 (v2.5.2) with default parameters (*67*). After generating sorted BAM files using Samtools (v1.19) (*68*), PCR duplicates were removed using Picard (v3.2.0). Peak calling was performed with MACS2 (v2.2.9.1) using default parameters for ATAC data, ETV2, TAL1, p300, CTCF, and H3K4me3 and “—broad” option for H3K4me1, H3K27ac, and H3K27me3 (*69*). All the replicates were merged and the signal tracks were generated by bamCoverage in Deeptools (v3.5.4) with the parameter “--normalizeUsing RPKM” (*70*). The tracks were visualized using the WashU Epigenome Browser. Heatmaps were generated using ±1 kb around the peak center by computeMatrix and plotHeatmap in Deeptools. The findMotifsGenome tools with default parameters (-size 400) from HOMER were used to enrich motifs (*71*). Chromatin state annotation was performed using ChromHMM (v1.25). Intersecting peaks between different groups were identified using intersect or window (-w 200) methods from BEDTools (v2.31.1) (*72*). Peak annotation was performed using the R package ChIPseeker (v1.40.0) (*73*, *74*). The H3K4me1, H3K4me3, H3K27ac, and H3K27me3 ChIP-seq datasets of hPSC were from GSE29611 (*75*). The TAL1 ChIP-seq datasets of ECFC, HSPC, and T-ALL were from GSE53423, GSE26014, and GSE33850 (*54*–*56*).

### Gene regulatory network analysis

ANANSE was used to identify the gene regulatory network (*38*). The ATAC-seq and H3K27ac ChIP-seq datasets and the differentially expressed genes of PSC-ETV2-24h cells and MPC-ETV2-24h cells were used as input for network inference. The influence score and target genes were calculated by ANANSE.

### Hi-C data analysis

Quality control and adapter trimming were performed as described above. The clean reads were aligned to the hg19 reference genome using HiC-Pro (v3.1.0) and the raw interaction matrix was normalized by using the ICE algorithm (*76*, *77*). The hic format file was generated using Juicer (v1.9.9) (*78*). The loops were visualized using the WashU Epigenome Browser.

### IP-MS data analysis

The raw MS data were processed using MaxQuant against the UniProt human proteome database. Considering the subcellular localization of ETV2 and TAL1, only proteins annotated as nuclear-localized proteins in UniProt were retained from all the proteins identified by MS. The proteins specific to the experimental group were then identified based on the intersection. Next, the proteins in each group were ranked according to their abundance and log-transformed for plotting. Proteins with no signal in the control group or with an abundance greater than five times that in the control group were considered significantly enriched in the experimental group.

### scRNA-seq data analysis

Raw sequencing data in FASTQ format were processed using the 10x Genomics Cell Ranger pipeline (v8.0.1). The raw reads were individually aligned to human reference genome GRCh38 (reference 2024-A) and quantified unique molecular identifiers counted by “cellranger count” for each gene in each cell. The count matrix of all samples was imported into the R environment using the function “Read10X” in the Seurat R package (v.5.3.0) (*79*). We removed cells with less than 200 genes, more than 7000 genes, or more than 5% mitochondrial reads. Only high-quality single-cell transcriptomes were used for subsequent analysis. The filtered data were then normalized using SCTransform. Dimensionality reduction was first performed via PCA. To account for potential batch effects and integrate datasets, Harmony was used to integrate data. The integrated data underwent further dimensionality reduction using the top 20 principal components (PCs) for Uniform Manifold Approximation and Projection (UMAP). Gene set signature scores were computed using the AddModuleScore function in Seurat. Each cluster’s differential expressed genes were identified using the FindAllMarkers function with default parameters. RNA velocity analysis was performed with scVelo (*80*). Gene regulatory network was calculated using pySCENIC (*81*).

### scATAC-seq data analysis

Raw sequencing data in FASTQ format were processed using the 10x Genomics Cell Ranger ATAC pipeline (v2.2.0). The raw reads were individually aligned to human reference genome GRCh38 (reference 2024-A). The cellranger-atac count command was used to generate fragment files for each sample. Downstream analysis was performed in the R environment using Signac (v1.16.0). Samples from two time points were integrated into a merged object for comparative analysis. Quality-control metrics were calculated and cells were filtered as follows: number of fragments, 3000 to 50,000; blacklist_fraction <0.05; nucleosome_signal <4; and transcription start site enrichment score > 2. Standard iterative latent semantic indexing dimension reduction and clustering was performed as follows using default parameters: RunTFIDF, FindTopFeatures, RunSVD, RunUMAP, FindNeighbors, and FindClusters. UMAP was used to visualize cell embedding for all cells. Gene scores were calculated using GeneActivity. To identify differentially accessible peaks across time points, scATAC-seq data were used to generate pseudo-bulk replicates based on cluster identities. Peak calling within each cluster was performed using MACS2 with default parameters. The signal tracks were generated by bamCoverage in Deeptools with the parameter “--normalizeUsing RPKM.” The tracks were visualized using the WashU Epigenome Browser.

### Statistical analysis

All quantitative data are presented as means ± SEM unless otherwise specified. Each experiment was performed with at least three independent biological replicates. For comparisons between two groups, an unpaired two-tailed Student’s *t* test was used. For multiple group comparisons, one-way or two-way analysis of variance (ANOVA) followed by Tukey’s post hoc test was used. The normality of data distribution was assessed using the Shapiro-Wilk test. No data were excluded from any analysis. All statistical evaluations were performed using GraphPad Prism 9.0 (GraphPad Software, San Diego, CA, USA) or R (v4.3.0). Statistical significance was defined as *P* < 0.05, with significance levels indicated as *P < 0.05, **P < 0.01, ***P < 0.001, and ****P < 0.0001.

## References

[1] A. Farber, R. T. Eberhardt, The current state of critical limb ischemia: A systematic review. JAMA Surg. 151, 1070–1077 (2016).27551978 10.1001/jamasurg.2016.2018

[2] P. Severino, A. D’Amato, M. Pucci, F. Infusino, F. Adamo, L. I. Birtolo, L. Netti, G. Montefusco, C. Chimenti, C. Lavalle, V. Maestrini, M. Mancone, W. M. Chilian, F. Fedele, Ischemic heart disease pathophysiology paradigms overview: From plaque activation to microvascular dysfunction. Int. J. Mol. Sci. 21, 8118 (2020).33143256 10.3390/ijms21218118PMC7663258

[3] Y. Hong, Y. Zhao, H. Li, Y. Yang, M. Chen, X. Wang, M. Luo, K. Wang, Engineering the maturation of stem cell-derived cardiomyocytes. Front. Bioeng. Biotechnol. 11, 1155052 (2023).37034258 10.3389/fbioe.2023.1155052PMC10073467

[4] Y. Zhao, X. Wang, K. Wang, Transcription factor-mediated programming of stem cell fate. Trends Cell Biol. 33, 621–624 (2023).37236901 10.1016/j.tcb.2023.05.004

[5] M. J. Pfeiffer, R. Quaranta, I. Piccini, J. Fell, J. Rao, A. Röpke, G. Seebohm, B. Greber, Cardiogenic programming of human pluripotent stem cells by dose-controlled activation of EOMES. Nat. Commun. 9, 440 (2018).29382828 10.1038/s41467-017-02812-6PMC5789885

[6] A. H. M. Ng, P. Khoshakhlagh, J. E. R. Arias, G. Pasquini, K. Wang, A. Swiersy, S. L. Shipman, E. Appleton, K. Kiaee, R. E. Kohman, A. Vernet, M. Dysart, K. Leeper, W. Saylor, J. Y. Huang, A. Graveline, J. Taipale, D. E. Hill, M. Vidal, J. M. Melero-Martin, V. Busskamp, G. M. Church, A comprehensive library of human transcription factors for cell fate engineering. Nat. Biotechnol. 39, 510–519 (2021).33257861 10.1038/s41587-020-0742-6PMC7610615

[7] L. Gong, Y. Zhang, Y. Zhu, U. Lee, A. C. Luo, X. Li, X. Wang, D. Chen, W. T. Pu, R.-Z. Lin, M. Ma, M. Cui, K. Chen, K. Wang, J. M. Melero-Martin, Rapid generation of functional vascular organoids via simultaneous transcription factor activation of endothelial and mural lineages. Cell Stem Cell 32, 1200–1217.e6 (2025).40516530 10.1016/j.stem.2025.05.014PMC12980400

[8] S. Albini, P. Coutinho, B. Malecova, L. Giordani, A. Savchenko, S. V. Forcales, P. L. Puri, Epigenetic reprogramming of human embryonic stem cells into skeletal muscle cells and generation of contractile myospheres. Cell Rep. 3, 661–670 (2013).23478022 10.1016/j.celrep.2013.02.012PMC3625045

[9] T. Akiyama, S. Wakabayashi, A. Soma, S. Sato, Y. Nakatake, M. Oda, M. Murakami, M. Sakota, N. Chikazawa-Nohtomi, S. B. H. Ko, M. S. H. Ko, Transient ectopic expression of the histone demethylase JMJD3 accelerates the differentiation of human pluripotent stem cells. Development 143, 3674–3685 (2016).27802135 10.1242/dev.139360PMC5087640

[10] R. L. Davis, H. Weintraub, A. B. Lassar, Expression of a single transfected cDNA converts fibroblasts to myoblasts. Cell 51, 987–1000 (1987).3690668 10.1016/0092-8674(87)90585-x

[11] I. Canals, A. Ginisty, E. Quist, R. Timmerman, J. Fritze, G. Miskinyte, E. Monni, M. G. Hansen, I. Hidalgo, D. Bryder, J. Bengzon, H. Ahlenius, Rapid and efficient induction of functional astrocytes from human pluripotent stem cells. Nat. Methods 15, 693–696 (2018).30127505 10.1038/s41592-018-0103-2

[12] H. Kataoka, M. Hayashi, R. Nakagawa, Y. Tanaka, N. Izumi, S. Nishikawa, M. L. Jakt, H. Tarui, S.-I. Nishikawa, Etv2/ER71 induces vascular mesoderm from Flk1^+^PDGFRα^+^ primitive mesoderm. Blood 118, 6975–6986 (2011).21911838 10.1182/blood-2011-05-352658

[13] A. Ferdous, A. Caprioli, M. Iacovino, C. M. Martin, J. Morris, J. A. Richardson, S. Latif, R. E. Hammer, R. P. Harvey, E. N. Olson, M. Kyba, D. J. Garry, Nkx2–5 transactivates the Ets-related protein 71 gene and specifies an endothelial/endocardial fate in the developing embryo. Proc. Natl. Acad. Sci. U.S.A. 106, 814–819 (2009).19129488 10.1073/pnas.0807583106PMC2630085

[14] N. Koyano-Nakagawa, D. J. Garry, ETV2 as an essential regulator of mesodermal lineage development. Cardiovasc. Res. 113, 1294–1306 (2017).28859300 10.1093/cvr/cvx133PMC5852632

[15] D. Alleyne, M. Kim, J. Wu, Y. Kwon, Y.-R. Kim, A. U. Kabir, M. Ishahak, J. R. Millman, C. Fan, H. J. Lee, K. Krchma, X. Xing, K. Lavine, T. Wang, K. Choi, Distinct contributions of Etv2^+^ and Flk1^+^ progenitors to endothelial, hematopoietic, and cardiac lineages. Cell Rep. 45, 116733 (2026).41420864 10.1016/j.celrep.2025.116733PMC13003410

[16] W. Gong, S. Das, J. E. Sierra-Pagan, E. Skie, N. Dsouza, T. A. Larson, M. G. Garry, E. Luzete-Monteiro, K. S. Zaret, D. J. Garry, ETV2 functions as a pioneer factor to regulate and reprogram the endothelial lineage. Nat. Cell Biol. 24, 672–684 (2022).35550615 10.1038/s41556-022-00901-3PMC11827897

[17] S. Das, X. Ma, K. Hailemariam, J. E. Sierra-Pagan, T. A. Larson, Y. G. Choi, A. Q. Le, R. J. Leonard, A. Cho, H. A. Sadek, J. J. Zhang, M. G. Garry, X. Dong, W. Gong, D. J. Garry, The pioneer factor, ETV2, regulates networks to specify the embryonic endothelial lineage. Cardiovasc. Res. 121, 1883–1897 (2025).40832854 10.1093/cvr/cvaf127PMC13370475

[18] J. D. Steimle, C. Kim, M. Rowton, R. D. Nadadur, Z. Wang, M. Stocker, A. D. Hoffmann, E. Hanson, J. Kweon, T. Sinha, K. Choi, B. L. Black, J. M. Cunningham, I. P. Moskowitz, K. Ikegami, ETV2 primes hematoendothelial gene enhancers prior to hematoendothelial fate commitment. Cell Rep. 42, 112665 (2023).37330911 10.1016/j.celrep.2023.112665PMC10592526

[19] B. Palikuqi, D.-H. T. Nguyen, G. Li, R. Schreiner, A. F. Pellegata, Y. Liu, D. Redmond, F. Geng, Y. Lin, J. M. Gómez-Salinero, M. Yokoyama, P. Zumbo, T. Zhang, B. Kunar, M. Witherspoon, T. Han, A. M. Tedeschi, F. Scottoni, S. M. Lipkin, L. Dow, O. Elemento, J. Z. Xiang, K. Shido, J. R. Spence, Q. J. Zhou, R. E. Schwartz, P. D. Coppi, S. Y. Rabbany, S. Rafii, Adaptable haemodynamic endothelial cells for organogenesis and tumorigenesis. Nature 585, 426–432 (2020).32908310 10.1038/s41586-020-2712-zPMC7480005

[20] S. Lee, C. Park, J. W. Han, J. Y. Kim, K. Cho, E. J. Kim, S. Kim, S.-J. Lee, S. Y. Oh, Y. Tanaka, I.-H. Park, H. J. An, C. M. Shin, S. Sharma, Y. Yoon, Direct reprogramming of human dermal fibroblasts into endothelial cells using ER71/ETV2. Circ. Res. 120, 848–861 (2017).28003219 10.1161/CIRCRESAHA.116.309833PMC5336520

[21] R. Morita, M. Suzuki, H. Kasahara, N. Shimizu, T. Shichita, T. Sekiya, A. Kimura, K. Sasaki, H. Yasukawa, A. Yoshimura, ETS transcription factor ETV2 directly converts human fibroblasts into functional endothelial cells. Proc. Natl. Acad. Sci. U.S.A. 112, 160–165 (2015).25540418 10.1073/pnas.1413234112PMC4291653

[22] A. Grath, G. Dai, SOX17/ETV2 improves the direct reprogramming of adult fibroblasts to endothelial cells. Cell Rep. Methods 4, 100732 (2024).38503291 10.1016/j.crmeth.2024.100732PMC10985233

[23] A. Margariti, B. Winkler, E. Karamariti, A. Zampetaki, T. Tsai, D. Baban, J. Ragoussis, Y. Huang, J.-D. J. Han, L. Zeng, Y. Hu, Q. Xu, Direct reprogramming of fibroblasts into endothelial cells capable of angiogenesis and reendothelialization in tissue-engineered vessels. Proc. Natl. Acad. Sci. U.S.A. 109, 13793–13798 (2012).22869753 10.1073/pnas.1205526109PMC3427074

[24] J.-K. Han, S.-H. Chang, H.-J. Cho, S.-B. Choi, H.-S. Ahn, J. Lee, H. Jeong, S.-W. Youn, H.-J. Lee, Y.-W. Kwon, H.-J. Cho, B.-H. Oh, P. Oettgen, Y.-B. Park, H.-S. Kim, Direct conversion of adult skin fibroblasts to endothelial cells by defined factors. Circulation 130, 1168–1178 (2014).25186941 10.1161/CIRCULATIONAHA.113.007727

[25] K. Wang, R.-Z. Lin, X. Hong, A. H. Ng, C. N. Lee, J. Neumeyer, G. Wang, X. Wang, M. Ma, W. T. Pu, G. M. Church, J. M. Melero-Martin, Robust differentiation of human pluripotent stem cells into endothelial cells via temporal modulation of ETV2 with modified mRNA. Sci. Adv. 6, eaba7606 (2020).32832668 10.1126/sciadv.aba7606PMC7439318

[26] J. Lv, S. Meng, Q. Gu, R. Zheng, X. Gao, J. Kim, M. Chen, B. Xia, Y. Zuo, S. Zhu, D. Zhao, Y. Li, G. Wang, X. Wang, Q. Meng, Q. Cao, J. P. Cooke, L. Fang, K. Chen, L. Zhang, Epigenetic landscape reveals MECOM as an endothelial lineage regulator. Nat. Commun. 14, 2390 (2023).37185814 10.1038/s41467-023-38002-wPMC10130150

[27] L. T. Ang, S. L. Zheng, K. J. Liu, A. Masaltseva, J. Winters, I. von Creytz, S. K. Jha, Q. Yin, C. Qian, X. Xiong, A. Dailamy, E. Xi, J. C. Alcocer, D. W. Sorensen, R. She, K. Smolyar, D. Szumska, S. Nornes, R. M. Martin, B. J. Lesch, N. K. Restrepo, W. Sun, J. S. Weissman, H. Lickert, M. P. Porteus, M. A. Skylar-Scott, C. Mosimann, S. Sumanas, S. D. Val, J. B. Prescott, K. Red-Horse, K. M. Loh, Discovery of a primed endothelial progenitor that requires VEGF/ERK inhibition to complete vein differentiation. bioRxiv 681838 [Preprint] (2025). 10.1101/2025.10.11.681838.

[28] B. Raftrey, I. M. Williams, P. E. R. Coronado, X. Fan, A. H. Chang, M. Zhao, R. Roth, E. Trimm, R. Racelis, G. D’Amato, R. Phansalkar, A. Nguyen, T. Chai, K. M. Gonzalez, Y. Zhang, L. T. Ang, K. M. Loh, D. Bernstein, K. Red-Horse, Dach1 extends artery networks and protects against cardiac injury. Circ. Res. 129, 702–716 (2021).34383559 10.1161/CIRCRESAHA.120.318271PMC8448957

[29] J. Chen, X. Zhang, D. M. DeLaughter, M. A. Trembley, S. Saifee, F. Xiao, J. Chen, P. Zhou, C. E. Seidman, J. G. Seidman, W. T. Pu, Molecular and spatial signatures of mouse embryonic endothelial cells at single-cell resolution. Circ. Res. 134, 529–546 (2024).38348657 10.1161/CIRCRESAHA.123.323956PMC10906678

[30] M. Corada, F. Orsenigo, M. F. Morini, M. E. Pitulescu, G. Bhat, D. Nyqvist, F. Breviario, V. Conti, A. Briot, M. L. Iruela-Arispe, R. H. Adams, E. Dejana, Sox17 is indispensable for acquisition and maintenance of arterial identity. Nat. Commun. 4, 2609 (2013).24153254 10.1038/ncomms3609PMC3826640

[31] J. Ernst, M. Kellis, Chromatin-state discovery and genome annotation with ChromHMM. Nat. Protoc. 12, 2478–2492 (2017).29120462 10.1038/nprot.2017.124PMC5945550

[32] L. S. Blok, T. Kleefstra, H. Venselaar, S. Maas, H. Y. Kroes, A. M. A. Lachmeijer, K. L. I. van Gassen, H. V. Firth, S. Tomkins, S. Bodek, T. D. Study, K. Õunap, M. H. Wojcik, C. Cunniff, K. Bergstrom, Z. Powis, S. Tang, D. N. Shinde, C. Au, A. D. Iglesias, K. Izumi, J. Leonard, A. A. Tayoun, S. W. Baker, M. Tartaglia, M. Niceta, M. L. Dentici, N. Okamoto, N. Miyake, N. Matsumoto, A. Vitobello, L. Faivre, C. Philippe, C. Gilissen, L. Wiel, R. Pfundt, P. Deriziotis, H. G. Brunner, S. E. Fisher, De novo variants disturbing the transactivation capacity of POU3F3 cause a characteristic neurodevelopmental disorder. Am. J. Hum. Genet. 105, 403–412 (2019).31303265 10.1016/j.ajhg.2019.06.007PMC6698880

[33] J. S. Byun, M. Oh, S. Lee, J.-E. Gil, Y. Mo, B. Ku, W.-K. Kim, K.-J. Oh, E.-W. Lee, K.-H. Bae, S. C. Lee, B.-S. Han, The transcription factor PITX1 drives astrocyte differentiation by regulating the SOX9 gene. J. Biol. Chem. 295, 13677–13690 (2020).32759168 10.1074/jbc.RA120.013352PMC7521637

[34] D. Chen, X. Fan, N. Sun, K. Wang, L. Gong, J. M. Melero-Martin, W. T. Pu, Pioneer factor ETV2 safeguards endothelial cell specification by recruiting the repressor REST to restrict alternative lineage commitment. Nat. Cardiovasc. Res. 4, 689–709 (2025).40495012 10.1038/s44161-025-00660-yPMC12836307

[35] A. G. Rosmarin, K. K. Resendes, Z. Yang, J. N. McMillan, S. L. Fleming, GA-binding protein transcription factor: A review of GABP as an integrator of intracellular signaling and protein–protein interactions. Blood Cells Mol. Dis. 32, 143–154 (2004).14757430 10.1016/j.bcmd.2003.09.005

[36] Y. Huang, S. J. Myers, R. Dingledine, Transcriptional repression by REST: Recruitment of Sin3A and histone deacetylase to neuronal genes. Nat. Neurosci. 2, 867–872 (1999).10491605 10.1038/13165

[37] J. M. Alexander, S. K. Hota, D. He, S. Thomas, L. Ho, L. A. Pennacchio, B. G. Bruneau, Brg1 modulates enhancer activation in mesoderm lineage commitment. Development 142, 1418–1430 (2015).25813539 10.1242/dev.109496PMC4392595

[38] Q. Xu, G. Georgiou, S. Frölich, M. van der Sande, G. J. C. Veenstra, H. Zhou, S. J. van Heeringen, ANANSE: An enhancer network-based computational approach for predicting key transcription factors in cell fate determination. Nucleic Acids Res. 49, 7966–7985 (2021).34244796 10.1093/nar/gkab598PMC8373078

[39] I. J. A. Peters, E. de Pater, W. Zhang, The role of GATA2 in adult hematopoiesis and cell fate determination. Front. Cell Dev. Biol. 11, 1250827 (2023).38033856 10.3389/fcell.2023.1250827PMC10682726

[40] C. Frelin, R. Herrington, S. Janmohamed, M. Barbara, G. Tran, C. J. Paige, P. Benveniste, J.-C. Zuñiga-Pflücker, A. Souabni, M. Busslinger, N. N. Iscove, GATA-3 regulates the self-renewal of long-term hematopoietic stem cells. Nat. Immunol. 14, 1037–1044 (2013).23974957 10.1038/ni.2692PMC4972578

[41] C. Porcher, H. Chagraoui, M. S. Kristiansen, SCL/TAL1: A multifaceted regulator from blood development to disease. Blood 129, 2051–2060 (2017).28179281 10.1182/blood-2016-12-754051

[42] X. L. Aranguren, M. Beerens, G. Coppiello, C. Wiese, I. Vandersmissen, A. L. Nigro, C. M. Verfaillie, M. Gessler, A. Luttun, COUP-TFII orchestrates venous and lymphatic endothelial identity by homo- or hetero-dimerisation with PROX1. J. Cell Sci. 126, 1164–1175 (2013).23345397 10.1242/jcs.116293

[43] S. Sissaoui, J. Yu, A. Yan, R. Li, O. Yukselen, A. Kucukural, L. J. Zhu, N. D. Lawson, Genomic characterization of endothelial enhancers reveals a multifunctional role for NR2F2 in regulation of arteriovenous gene expression. Circ. Res. 126, 875–888 (2020).32065070 10.1161/CIRCRESAHA.119.316075PMC7212523

[44] B. A. Benayoun, E. A. Pollina, D. Ucar, S. Mahmoudi, K. Karra, E. D. Wong, K. Devarajan, A. C. Daugherty, A. B. Kundaje, E. Mancini, B. C. Hitz, R. Gupta, T. A. Rando, J. C. Baker, M. P. Snyder, J. M. Cherry, A. Brunet, H3K4me3 breadth is linked to cell identity and transcriptional consistency. Cell 163, 1281–1286 (2015).28930648 10.1016/j.cell.2015.10.051

[45] K. Chen, Z. Chen, D. Wu, L. Zhang, X. Lin, J. Su, B. Rodriguez, Y. Xi, Z. Xia, X. Chen, X. Shi, Q. Wang, W. Li, Broad H3K4me3 is associated with increased transcription elongation and enhancer activity at tumor-suppressor genes. Nat. Genet. 47, 1149–1157 (2015).26301496 10.1038/ng.3385PMC4780747

[46] S. Heinz, C. E. Romanoski, C. Benner, C. K. Glass, The selection and function of cell type-specific enhancers. Nat. Rev. Mol. Cell Biol. 16, 144–154 (2015).25650801 10.1038/nrm3949PMC4517609

[47] I. Elcheva, V. Brok-Volchanskaya, A. Kumar, P. Liu, J.-H. Lee, L. Tong, M. Vodyanik, S. Swanson, R. Stewart, M. Kyba, E. Yakubov, J. Cooke, J. A. Thomson, I. Slukvin, Direct induction of haematoendothelial programs in human pluripotent stem cells by transcriptional regulators. Nat. Commun. 5, 4372 (2014).25019369 10.1038/ncomms5372PMC4107340

[48] C. Porcher, W. Swat, K. Rockwell, Y. Fujiwara, F. W. Alt, S. H. Orkin, The T cell leukemia oncoprotein SCL/tal-1 is essential for development of all hematopoietic lineages. Cell 86, 47–57 (1996).8689686 10.1016/s0092-8674(00)80076-8

[49] R. A. Shivdasani, E. L. Mayer, S. H. Orkin, Absence of blood formation in mice lacking the T-cell leukaemia oncoprotein tal-1/SCL. Nature 373, 432–434 (1995).7830794 10.1038/373432a0

[50] L. Robb, N. J. Elwood, A. G. Elefanty, F. Köntgen, R. Li, L. D. Barnett, C. G. Begley, The scl gene product is required for the generation of all hematopoietic lineages in the adult mouse. EMBO J. 15, 4123–4129 (1996).8861941 PMC452135

[51] H. Zhao, K. Choi, A CRISPR screen identifies genes controlling Etv2 threshold expression in murine hemangiogenic fate commitment. Nat. Commun. 8, 541 (2017).28912455 10.1038/s41467-017-00667-5PMC5599515

[52] J. E. Visvader, Y. Fujiwara, S. H. Orkin, Unsuspected role for the T-cell leukemia protein SCL/tal-1 in vascular development. Genes Dev. 12, 473–479 (1998).9472016 10.1101/gad.12.4.473PMC316527

[53] B. Pijuan-Sala, J. A. Griffiths, C. Guibentif, T. W. Hiscock, W. Jawaid, F. J. Calero-Nieto, C. Mulas, X. Ibarra-Soria, R. C. V. Tyser, D. L. L. Ho, W. Reik, S. Srinivas, B. D. Simons, J. Nichols, J. C. Marioni, B. Göttgens, A single-cell molecular map of mouse gastrulation and early organogenesis. Nature 566, 490–495 (2019).30787436 10.1038/s41586-019-0933-9PMC6522369

[54] N. Novershtern, A. Subramanian, L. N. Lawton, R. H. Mak, W. N. Haining, M. E. McConkey, N. Habib, N. Yosef, C. Y. Chang, T. Shay, G. M. Frampton, A. C. B. Drake, I. Leskov, B. Nilsson, F. Preffer, D. Dombkowski, J. W. Evans, T. Liefeld, J. S. Smutko, J. Chen, N. Friedman, R. A. Young, T. R. Golub, A. Regev, B. L. Ebert, Densely interconnected transcriptional circuits control cell states in human hematopoiesis. Cell 144, 296–309 (2011).21241896 10.1016/j.cell.2011.01.004PMC3049864

[55] T. Sanda, L. N. Lawton, M. I. Barrasa, Z. P. Fan, H. Kohlhammer, A. Gutierrez, W. Ma, J. Tatarek, Y. Ahn, M. A. Kelliher, C. H. M. Jamieson, L. M. Staudt, R. A. Young, A. T. Look, Core transcriptional regulatory circuit controlled by the TAL1 complex in human T cell acute lymphoblastic leukemia. Cancer Cell 22, 209–221 (2012).22897851 10.1016/j.ccr.2012.06.007PMC3422504

[56] C. G. Palii, B. Vulesevic, S. Fraineau, E. Pranckeviciene, A. J. Griffith, A. Chu, H. Faralli, Y. Li, B. McNeill, J. Sun, T. J. Perkins, F. J. Dilworth, C. Perez-Iratxeta, E. J. Suuronen, D. S. Allan, M. Brand, Trichostatin A enhances vascular repair by injected human endothelial progenitors through increasing the expression of TAL1-dependent genes. Cell Stem Cell 14, 644–657 (2014).24792117 10.1016/j.stem.2014.03.003

[57] D. Hnisz, B. J. Abraham, T. I. Lee, A. Lau, V. Saint-André, A. A. Sigova, H. A. Hoke, R. A. Young, Super-enhancers in the control of cell identity and disease. Cell 155, 934–947 (2013).24119843 10.1016/j.cell.2013.09.053PMC3841062

[58] J. Lovén, H. A. Hoke, C. Y. Lin, A. Lau, D. A. Orlando, C. R. Vakoc, J. E. Bradner, T. I. Lee, R. A. Young, Selective inhibition of tumor oncogenes by disruption of super-enhancers. Cell 153, 320–334 (2013).23582323 10.1016/j.cell.2013.03.036PMC3760967

[59] W. A. Whyte, D. A. Orlando, D. Hnisz, B. J. Abraham, C. Y. Lin, M. H. Kagey, P. B. Rahl, T. I. Lee, R. A. Young, Master transcription factors and mediator establish super-enhancers at key cell identity genes. Cell 153, 307–319 (2013).23582322 10.1016/j.cell.2013.03.035PMC3653129

[60] J. Abramson, J. Adler, J. Dunger, R. Evans, T. Green, A. Pritzel, O. Ronneberger, L. Willmore, A. J. Ballard, J. Bambrick, S. W. Bodenstein, D. A. Evans, C.-C. Hung, M. O’Neill, D. Reiman, K. Tunyasuvunakool, Z. Wu, A. Žemgulytė, E. Arvaniti, C. Beattie, O. Bertolli, A. Bridgland, A. Cherepanov, M. Congreve, A. I. Cowen-Rivers, A. Cowie, M. Figurnov, F. B. Fuchs, H. Gladman, R. Jain, Y. A. Khan, C. M. R. Low, K. Perlin, A. Potapenko, P. Savy, S. Singh, A. Stecula, A. Thillaisundaram, C. Tong, S. Yakneen, E. D. Zhong, M. Zielinski, A. Žídek, V. Bapst, P. Kohli, M. Jaderberg, D. Hassabis, J. M. Jumper, Accurate structure prediction of biomolecular interactions with AlphaFold 3. Nature 630, 493–500 (2024).38718835 10.1038/s41586-024-07487-wPMC11168924

[61] N. Zhang, Y. Chen, H. Lu, F. Zhao, R. V. Alvarez, A. Goncearenco, A. R. Panchenko, M. Li, MutaBind2: Predicting the impacts of single and multiple mutations on protein-protein interactions. iScience 23, 100939 (2020).32169820 10.1016/j.isci.2020.100939PMC7068639

[62] K. Li, Y. Liu, H. Cao, Y. Zhang, Z. Gu, X. Liu, A. Yu, P. Kaphle, K. E. Dickerson, M. Ni, J. Xu, Interrogation of enhancer function by enhancer-targeting CRISPR epigenetic editing. Nat. Commun. 11, 485 (2020).31980609 10.1038/s41467-020-14362-5PMC6981169

[63] A. Dobin, C. A. Davis, F. Schlesinger, J. Drenkow, C. Zaleski, S. Jha, P. Batut, M. Chaisson, T. R. Gingeras, STAR: Ultrafast universal RNA-seq aligner. Bioinformatics 29, 15–21 (2013).23104886 10.1093/bioinformatics/bts635PMC3530905

[64] Y. Liao, G. K. Smyth, W. Shi, featureCounts: An efficient general purpose program for assigning sequence reads to genomic features. Bioinformatics 30, 923–930 (2014).24227677 10.1093/bioinformatics/btt656

[65] M. I. Love, W. Huber, S. Anders, Moderated estimation of fold change and dispersion for RNA-seq data with DESeq2. Genome Biol. 15, 550 (2014).25516281 10.1186/s13059-014-0550-8PMC4302049

[66] T. Wu, E. Hu, S. Xu, M. Chen, P. Guo, Z. Dai, T. Feng, L. Zhou, W. Tang, L. Zhan, X. Fu, S. Liu, X. Bo, G. Yu, clusterProfiler 4.0: A universal enrichment tool for interpreting omics data. Innovation 2, 100141 (2021).34557778 10.1016/j.xinn.2021.100141PMC8454663

[67] B. Langmead, S. L. Salzberg, Fast gapped-read alignment with Bowtie 2. Nat. Methods 9, 357–359 (2012).22388286 10.1038/nmeth.1923PMC3322381

[68] P. Danecek, J. K. Bonfield, J. Liddle, J. Marshall, V. Ohan, M. O. Pollard, A. Whitwham, T. Keane, S. A. McCarthy, R. M. Davies, H. Li, Twelve years of SAMtools and BCFtools. GigaScience 10, giab008 (2021).33590861 10.1093/gigascience/giab008PMC7931819

[69] Y. Zhang, T. Liu, C. A. Meyer, J. Eeckhoute, D. S. Johnson, B. E. Bernstein, C. Nusbaum, R. M. Myers, M. Brown, W. Li, X. S. Liu, Model-based analysis of ChIP-Seq (MACS). Genome Biol. 9, R137 (2008).18798982 10.1186/gb-2008-9-9-r137PMC2592715

[70] F. Ramírez, D. P. Ryan, B. Grüning, V. Bhardwaj, F. Kilpert, A. S. Richter, S. Heyne, F. Dündar, T. Manke, deepTools2: A next generation web server for deep-sequencing data analysis. Nucleic Acids Res. 44, W160–W165 (2016).27079975 10.1093/nar/gkw257PMC4987876

[71] S. Heinz, C. Benner, N. Spann, E. Bertolino, Y. C. Lin, P. Laslo, J. X. Cheng, C. Murre, H. Singh, C. K. Glass, Simple combinations of lineage-determining transcription factors prime cis-regulatory elements required for macrophage and B cell identities. Mol. Cell 38, 576–589 (2010).20513432 10.1016/j.molcel.2010.05.004PMC2898526

[72] A. R. Quinlan, I. M. Hall, BEDTools: A flexible suite of utilities for comparing genomic features. Bioinformatics 26, 841–842 (2010).20110278 10.1093/bioinformatics/btq033PMC2832824

[73] G. Yu, L.-G. Wang, Q.-Y. He, ChIPseeker: An R/Bioconductor package for ChIP peak annotation, comparison and visualization. Bioinformatics 31, 2382–2383 (2015).25765347 10.1093/bioinformatics/btv145

[74] Q. Wang, M. Li, T. Wu, L. Zhan, L. Li, M. Chen, W. Xie, Z. Xie, E. Hu, S. Xu, G. Yu, Exploring epigenomic datasets by ChIPseeker. Curr. Protoc. 2, e585 (2022).36286622 10.1002/cpz1.585

[75] I. Dunham, A. Kundaje, S. F. Aldred, P. J. Collins, C. A. Davis, F. Doyle, C. B. Epstein, S. Frietze, J. Harrow, R. Kaul, J. Khatun, B. R. Lajoie, S. G. Landt, B.-K. Lee, F. Pauli, K. R. Rosenbloom, P. Sabo, A. Safi, A. Sanyal, N. Shoresh, J. M. Simon, L. Song, N. D. Trinklein, R. C. Altshuler, E. Birney, J. B. Brown, C. Cheng, S. Djebali, X. Dong, I. Dunham, J. Ernst, T. S. Furey, M. Gerstein, B. Giardine, M. Greven, R. C. Hardison, R. S. Harris, J. Herrero, M. M. Hoffman, S. Iyer, M. Kellis, J. Khatun, P. Kheradpour, A. Kundaje, T. Lassmann, Q. Li, X. Lin, G. K. Marinov, A. Merkel, A. Mortazavi, S. C. J. Parker, T. E. Reddy, J. Rozowsky, F. Schlesinger, R. E. Thurman, J. Wang, L. D. Ward, T. W. Whitfield, S. P. Wilder, W. Wu, H. S. Xi, K. Y. Yip, J. Zhuang, B. E. Bernstein, E. Birney, I. Dunham, E. D. Green, C. Gunter, M. Snyder, M. J. Pazin, R. F. Lowdon, L. A. L. Dillon, L. B. Adams, C. J. Kelly, J. Zhang, J. R. Wexler, E. D. Green, P. J. Good, E. A. Feingold, B. E. Bernstein, E. Birney, G. E. Crawford, J. Dekker, L. Elnitski, P. J. Farnham, M. Gerstein, M. C. Giddings, T. R. Gingeras, E. D. Green, R. Guigó, R. C. Hardison, T. J. Hubbard, M. Kellis, W. J. Kent, J. D. Lieb, E. H. Margulies, R. M. Myers, M. Snyder, J. A. Stamatoyannopoulos, S. A. Tenenbaum, Z. Weng, K. P. White, B. Wold, J. Khatun, Y. Yu, J. Wrobel, B. A. Risk, H. P. Gunawardena, H. C. Kuiper, C. W. Maier, L. Xie, X. Chen, M. C. Giddings, B. E. Bernstein, C. B. Epstein, N. Shoresh, J. Ernst, P. Kheradpour, T. S. Mikkelsen, S. Gillespie, A. Goren, O. Ram, X. Zhang, L. Wang, R. Issner, M. J. Coyne, T. Durham, M. Ku, T. Truong, L. D. Ward, R. C. Altshuler, M. L. Eaton, M. Kellis, S. Djebali, C. A. Davis, A. Merkel, A. Dobin, T. Lassmann, A. Mortazavi, A. Tanzer, J. Lagarde, W. Lin, F. Schlesinger, C. Xue, G. K. Marinov, J. Khatun, B. A. Williams, C. Zaleski, J. Rozowsky, M. Röder, F. Kokocinski, R. F. Abdelhamid, T. Alioto, I. Antoshechkin, M. T. Baer, P. Batut, I. Bell, K. Bell, S. Chakrabortty, X. Chen, J. Chrast, J. Curado, T. Derrien, J. Drenkow, E. Dumais, J. Dumais, R. Duttagupta, M. Fastuca, K. Fejes-Toth, P. Ferreira, S. Foissac, M. J. Fullwood, H. Gao, D. Gonzalez, A. Gordon, H. P. Gunawardena, C. Howald, S. Jha, R. Johnson, P. Kapranov, B. King, C. Kingswood, G. Li, O. J. Luo, E. Park, J. B. Preall, K. Presaud, P. Ribeca, B. A. Risk, D. Robyr, X. Ruan, M. Sammeth, K. S. Sandhu, L. Schaeffer, L.-H. See, A. Shahab, J. Skancke, A. M. Suzuki, H. Takahashi, H. Tilgner, D. Trout, N. Walters, H. Wang, J. Wrobel, Y. Yu, Y. Hayashizaki, J. Harrow, M. Gerstein, T. J. Hubbard, A. Reymond, S. E. Antonarakis, G. J. Hannon, M. C. Giddings, Y. Ruan, B. Wold, P. Carninci, R. Guigó, T. R. Gingeras, K. R. Rosenbloom, C. A. Sloan, K. Learned, V. S. Malladi, M. C. Wong, G. P. Barber, M. S. Cline, T. R. Dreszer, S. G. Heitner, D. Karolchik, W. J. Kent, V. M. Kirkup, L. R. Meyer, J. C. Long, M. Maddren, B. J. Raney, T. S. Furey, L. Song, L. L. Grasfeder, P. G. Giresi, B.-K. Lee, A. Battenhouse, N. C. Sheffield, J. M. Simon, K. A. Showers, A. Safi, D. London, A. A. Bhinge, C. Shestak, M. R. Schaner, S. K. Kim, Z. Z. Zhang, P. A. Mieczkowski, J. O. Mieczkowska, Z. Liu, R. M. McDaniell, Y. Ni, N. U. Rashid, M. J. Kim, S. Adar, Z. Zhang, T. Wang, D. Winter, D. Keefe, E. Birney, V. R. Iyer, J. D. Lieb, G. E. Crawford, G. Li, K. S. Sandhu, M. Zheng, P. Wang, O. J. Luo, A. Shahab, M. J. Fullwood, X. Ruan, Y. Ruan, R. M. Myers, F. Pauli, B. A. Williams, J. Gertz, G. K. Marinov, T. E. Reddy, J. Vielmetter, E. Partridge, D. Trout, K. E. Varley, C. Gasper, A. Bansal, S. Pepke, P. Jain, H. Amrhein, K. M. Bowling, M. Anaya, M. K. Cross, B. King, M. A. Muratet, I. Antoshechkin, K. M. Newberry, K. McCue, A. S. Nesmith, K. I. Fisher-Aylor, B. Pusey, G. DeSalvo, S. L. Parker, S. Balasubramanian, N. S. Davis, S. K. Meadows, T. Eggleston, C. Gunter, J. S. Newberry, S. E. Levy, D. M. Absher, A. Mortazavi, W. H. Wong, B. Wold, M. J. Blow, A. Visel, L. A. Pennachio, L. Elnitski, E. H. Margulies, S. C. J. Parker, H. M. Petrykowska, A. Abyzov, B. Aken, D. Barrell, G. Barson, A. Berry, A. Bignell, V. Boychenko, G. Bussotti, J. Chrast, C. Davidson, T. Derrien, G. Despacio-Reyes, M. Diekhans, I. Ezkurdia, A. Frankish, J. Gilbert, J. M. Gonzalez, E. Griffiths, R. Harte, D. A. Hendrix, C. Howald, T. Hunt, I. Jungreis, M. Kay, E. Khurana, F. Kokocinski, J. Leng, M. F. Lin, J. Loveland, Z. Lu, D. Manthravadi, M. Mariotti, J. Mudge, G. Mukherjee, C. Notredame, B. Pei, J. M. Rodriguez, G. Saunders, A. Sboner, S. Searle, C. Sisu, C. Snow, C. Steward, A. Tanzer, E. Tapanari, M. L. Tress, M. J. van Baren, N. Walters, S. Washietl, L. Wilming, A. Zadissa, Z. Zhang, M. Brent, D. Haussler, M. Kellis, A. Valencia, M. Gerstein, A. Reymond, R. Guigó, J. Harrow, T. J. Hubbard, S. G. Landt, S. Frietze, A. Abyzov, N. Addleman, R. P. Alexander, R. K. Auerbach, S. Balasubramanian, K. Bettinger, N. Bhardwaj, A. P. Boyle, A. R. Cao, P. Cayting, A. Charos, Y. Cheng, C. Cheng, C. Eastman, G. Euskirchen, J. D. Fleming, F. Grubert, L. Habegger, M. Hariharan, A. Harmanci, S. Iyengar, V. X. Jin, K. J. Karczewski, M. Kasowski, P. Lacroute, H. Lam, N. Lamarre-Vincent, J. Leng, J. Lian, M. Lindahl-Allen, R. Min, B. Miotto, H. Monahan, Z. Moqtaderi, X. J. Mu, H. O’Geen, Z. Ouyang, D. Patacsil, B. Pei, D. Raha, L. Ramirez, B. Reed, J. Rozowsky, A. Sboner, M. Shi, C. Sisu, T. Slifer, H. Witt, L. Wu, X. Xu, K.-K. Yan, X. Yang, K. Y. Yip, Z. Zhang, K. Struhl, S. M. Weissman, M. Gerstein, P. J. Farnham, M. Snyder, S. A. Tenenbaum, L. O. Penalva, F. Doyle, S. Karmakar, S. G. Landt, R. R. Bhanvadia, A. Choudhury, M. Domanus, L. Ma, J. Moran, D. Patacsil, T. Slifer, A. Victorsen, X. Yang, M. Snyder, K. P. White, T. Auer, L. Centanin, M. Eichenlaub, F. Gruhl, S. Heermann, B. Hoeckendorf, D. Inoue, T. Kellner, S. Kirchmaier, C. Mueller, R. Reinhardt, L. Schertel, S. Schneider, R. Sinn, B. Wittbrodt, J. Wittbrodt, Z. Weng, T. W. Whitfield, J. Wang, P. J. Collins, S. F. Aldred, N. D. Trinklein, E. C. Partridge, R. M. Myers, J. Dekker, G. Jain, B. R. Lajoie, A. Sanyal, G. Balasundaram, D. L. Bates, R. Byron, T. K. Canfield, M. J. Diegel, D. Dunn, A. K. Ebersol, T. Frum, K. Garg, E. Gist, R. S. Hansen, L. Boatman, E. Haugen, R. Humbert, G. Jain, A. K. Johnson, E. M. Johnson, T. V. Kutyavin, B. R. Lajoie, K. Lee, D. Lotakis, M. T. Maurano, S. J. Neph, F. V. Neri, E. D. Nguyen, H. Qu, A. P. Reynolds, V. Roach, E. Rynes, P. Sabo, M. E. Sanchez, R. S. Sandstrom, A. Sanyal, A. O. Shafer, A. B. Stergachis, S. Thomas, R. E. Thurman, B. Vernot, J. Vierstra, S. Vong, H. Wang, M. A. Weaver, Y. Yan, M. Zhang, J. M. Akey, M. Bender, M. O. Dorschner, M. Groudine, M. J. MacCoss, P. Navas, G. Stamatoyannopoulos, R. Kaul, J. Dekker, J. A. Stamatoyannopoulos, I. Dunham, K. Beal, A. Brazma, P. Flicek, J. Herrero, N. Johnson, D. Keefe, M. Lukk, N. M. Luscombe, D. Sobral, J. M. Vaquerizas, S. P. Wilder, S. Batzoglou, A. Sidow, N. Hussami, S. Kyriazopoulou-Panagiotopoulou, M. W. Libbrecht, M. A. Schaub, A. Kundaje, R. C. Hardison, W. Miller, B. Giardine, R. S. Harris, W. Wu, P. J. Bickel, B. Banfai, N. P. Boley, J. B. Brown, H. Huang, Q. Li, J. J. Li, W. S. Noble, J. A. Bilmes, O. J. Buske, M. M. Hoffman, A. D. Sahu, P. V. Kharchenko, P. J. Park, D. Baker, J. Taylor, Z. Weng, S. Iyer, X. Dong, M. Greven, X. Lin, J. Wang, H. S. Xi, J. Zhuang, M. Gerstein, R. P. Alexander, S. Balasubramanian, C. Cheng, A. Harmanci, L. Lochovsky, R. Min, X. J. Mu, J. Rozowsky, K.-K. Yan, K. Y. Yip, E. Birney, An integrated encyclopedia of DNA elements in the human genome. Nature 489, 57–74 (2012).22955616 10.1038/nature11247PMC3439153

[76] N. Servant, N. Varoquaux, B. R. Lajoie, E. Viara, C.-J. Chen, J.-P. Vert, E. Heard, J. Dekker, E. Barillot, HiC-Pro: An optimized and flexible pipeline for Hi-C data processing. Genome Biol. 16, 259 (2015).26619908 10.1186/s13059-015-0831-xPMC4665391

[77] M. Imakaev, G. Fudenberg, R. P. McCord, N. Naumova, A. Goloborodko, B. R. Lajoie, J. Dekker, L. A. Mirny, Iterative correction of Hi-C data reveals hallmarks of chromosome organization. Nat. Methods 9, 999–1003 (2012).22941365 10.1038/nmeth.2148PMC3816492

[78] N. C. Durand, J. T. Robinson, M. S. Shamim, I. Machol, J. P. Mesirov, E. S. Lander, E. L. Aiden, Juicebox provides a visualization system for Hi-C contact maps with unlimited zoom. Cell Syst. 3, 99–101 (2016).27467250 10.1016/j.cels.2015.07.012PMC5596920

[79] Y. Hao, T. Stuart, M. H. Kowalski, S. Choudhary, P. Hoffman, A. Hartman, A. Srivastava, G. Molla, S. Madad, C. Fernandez-Granda, R. Satija, Dictionary learning for integrative, multimodal and scalable single-cell analysis. Nat. Biotechnol. 42, 293–304 (2024).37231261 10.1038/s41587-023-01767-yPMC10928517

[80] V. Bergen, M. Lange, S. Peidli, F. A. Wolf, F. J. Theis, Generalizing RNA velocity to transient cell states through dynamical modeling. Nat. Biotechnol. 38, 1408–1414 (2020).32747759 10.1038/s41587-020-0591-3

[81] B. V. de Sande, C. Flerin, K. Davie, M. D. Waegeneer, G. Hulselmans, S. Aibar, R. Seurinck, W. Saelens, R. Cannoodt, Q. Rouchon, T. Verbeiren, D. D. Maeyer, J. Reumers, Y. Saeys, S. Aerts, A scalable SCENIC workflow for single-cell gene regulatory network analysis. Nat. Protoc. 15, 2247–2276 (2020).32561888 10.1038/s41596-020-0336-2

