# Supplementary material for: TAL1 is indispensable in ETV2-primed human endothelial cell fate determination
Source: Sci Adv. 2026 Aug 12;12(33):eaef9769. doi: 10.1126/sciadv.aef9769 (PMC13464644; doi:10.1126/sciadv.aef9769)
Supplement: Supplementary file 1 [file sciadv.aef9769_sm.pdf]

Science Advances
AAAS

Supplementary Materials for

TAL1 is indispensable in ETV2-primed human endothelial cell fate determination

Yun Zhao *et al.*

Corresponding author: Yuan Zhou, zhouyuanbioinfo@hsc.pku.edu.cn; Kailong Li, kailongli@pku.edu.cn; Xi Wang, xi_wang@hsc.pku.edu.cn; Kai Wang, kai.wang88@pku.edu.cn

Sci. Adv. **12**, eaef9769 (2026)
DOI: 10.1126/sciadv.aef9769

This PDF file includes:

Figs. S1 to S13
Tables S1 and S2

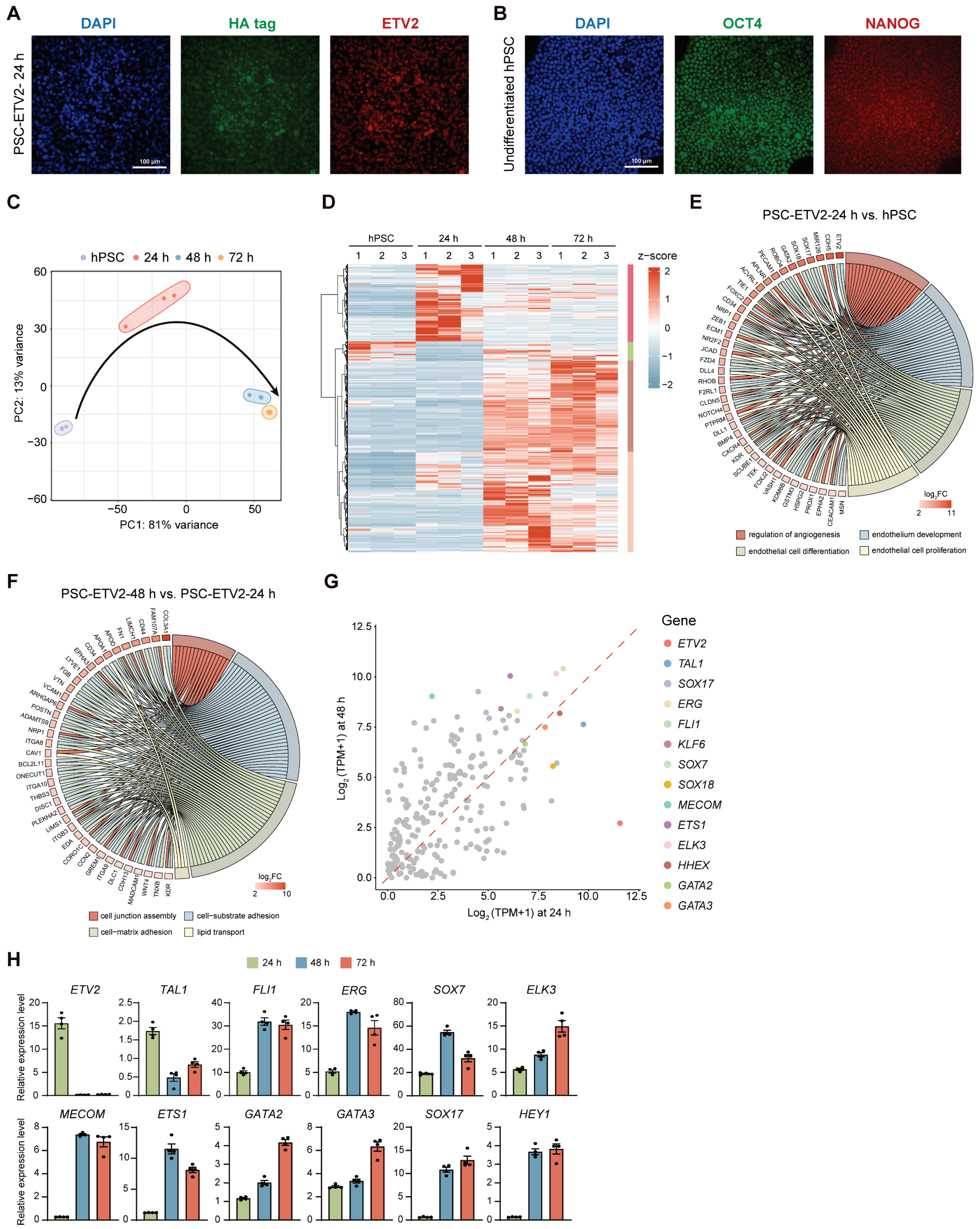

Fig. S1. Cell line characterization and RNA sequencing analysis.

(**A**) Representative immunofluorescence images revealing the co-localization of ETV2 and HA tag in iETV2-3×HA cell line after adding doxycycline. (**B**) Representative immunofluorescence images revealing the expression of OCT4 and NANOG in the established cell line. (**C**) Principal component analysis of the transcriptome of PSC-ETV2-24h, 48h and 72h cells. (**D**) Differentially expressed genes among three timepoints. (**E**) GO enrichment analysis of the genes upregulated in PSC-ETV2-24h cells. (**F**) GO enrichment analysis of the genes upregulated in PSC-ETV2-48h cells. (**G**) Dot plot showing the expression level of transcription

factors at 24 hours and 48 hours. (**H**) RT-qPCR analysis of the selected EC-specific transcription factors (*n*=4). Scale bar: 100 μm in (**A** and **B**).

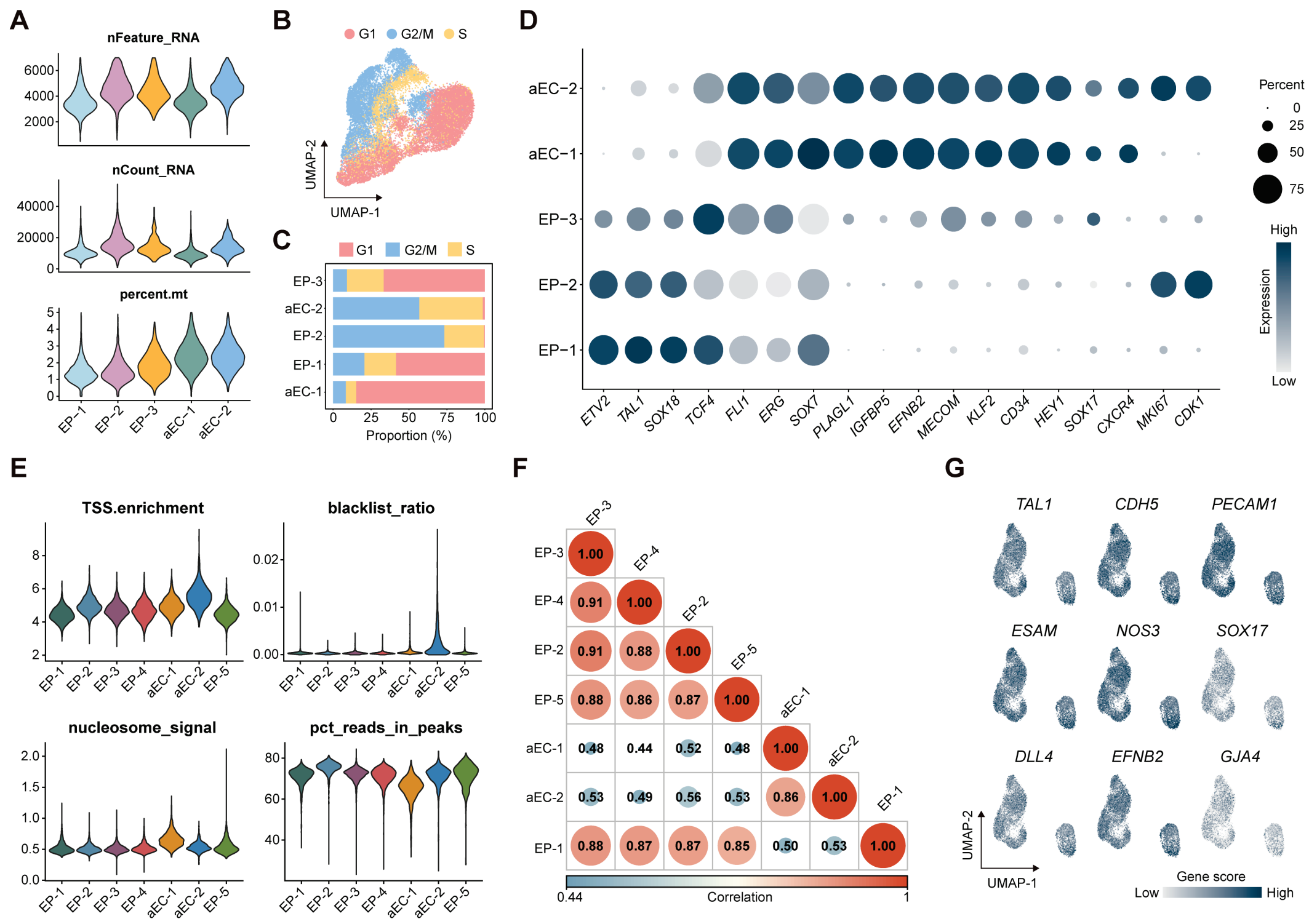

Fig. S2. Single-cell RNA sequencing and single-cell ATAC sequencing analysis of endothelial cell differentiation.

(**A**) Quality control metrics of single-cell RNA sequencing data. Number of genes (nFeature_RNA), number of molecular (nCount_RNA), and percentage of mitochondrial counts (percent.mt) are plotted for each cluster. (**B**) Uniform manifold approximation and projection (UMAP) visualization of cells colored according to cell cycle phase. (**C**) Distribution of cell cycle phases within each cluster. (**D**) Dot plot showing the expression patterns of marker genes across identified clusters. (**E**) Quality control metrics of single-cell ATAC sequencing data, including TSS enrichment scores (TSS.enrichment), fragment ratios in genomic blacklist regions (blacklist_ratio), nucleosome signal (nucleosome_signal) and the percentage of reads mapping to peaks (pct_reads_in_peaks). (**F**) Correlation analysis demonstrating the epigenetic similarity between individual single-cell ATAC sequencing clusters (**G**) UMAP visualization illustrating cells colored by their respective gene activity scores for representative markers.

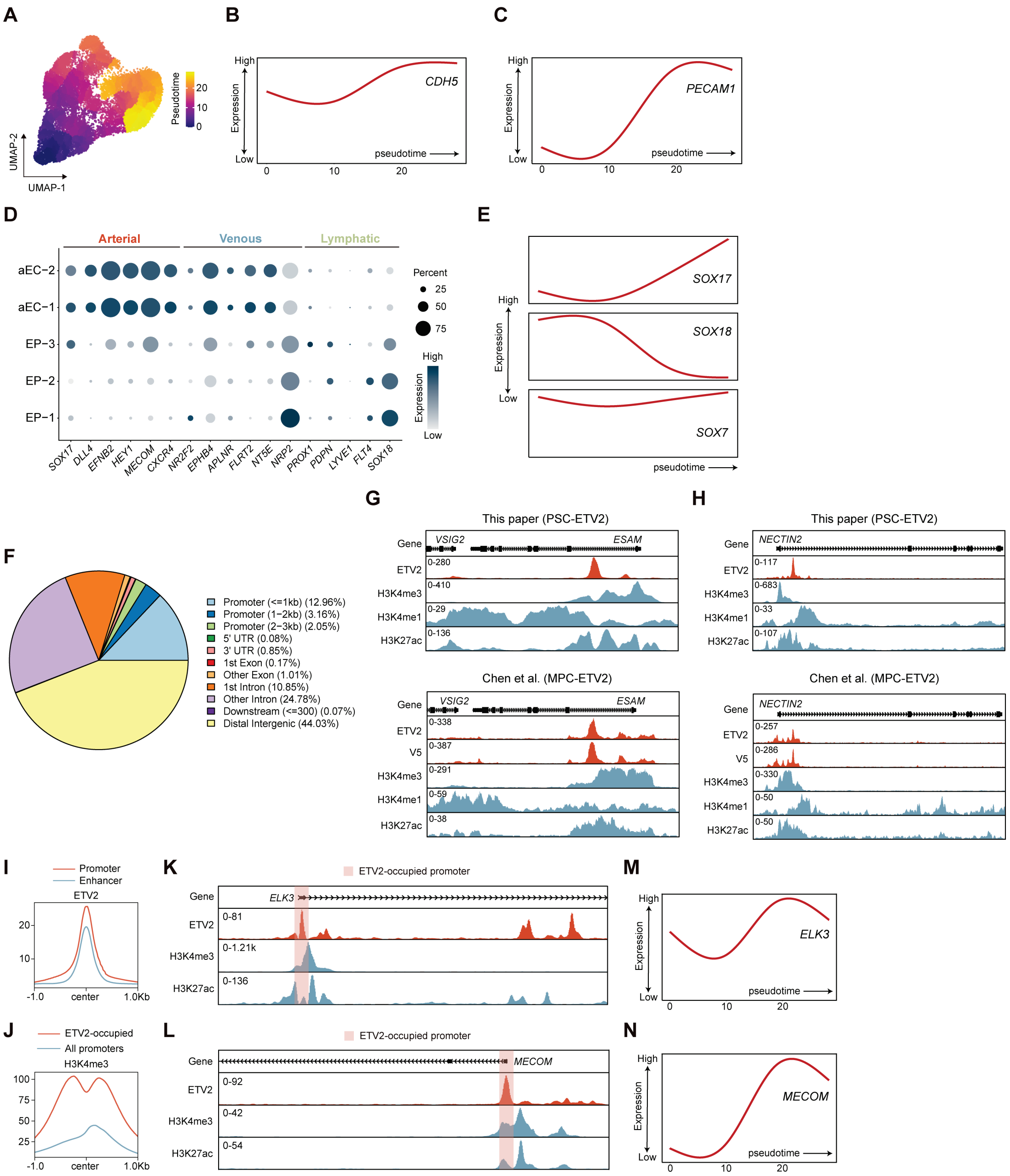

Fig. S3. ETV2 directly binds on promoters.

(**A**) Uniform manifold approximation and projection (UMAP) visualization of cells colored by developmental pseudotime. (**B**) Expression kinetics of the pan-EC marker *CDH5* along pseudotime. (**C**) Expression kinetics of the pan-EC marker *PECAM1* along pseudotime. (**D**) Dot plot showing the expression of arterial, venous, and lymphatic module genes across identified clusters. (**E**) Expression kinetics of SOXF family transcription factor, *SOX7*, *SOX17* and *SOX18* along pseudotime. (**F**) Pie chart showing the binding preference of ETV2. (**G**) Genome browser views showing the conserved binding sites of ETV2 and similar pattern of histone modifications around *ESAM*. (**H**) Genome browser views showing the conserved binding sites of ETV2 and similar pattern of histone modifications around *NECTIN2*. (**I**) Average signal of

ETV2 at ETV2-bound promoters or enhancers. (**J)** Average signal of H3K4me3 at ETV2-occupied promoters or all promoters. (**K**) Genome browser views showing ETV2 binding on the promoters of *ELK3*. (**L**) Genome browser views showing ETV2 binding on the promoters of *MECOM*. (**M**) Expression kinetics of *ELK3* along pseudotime. (**N**) Expression kinetics of *MECOM* along pseudotime.

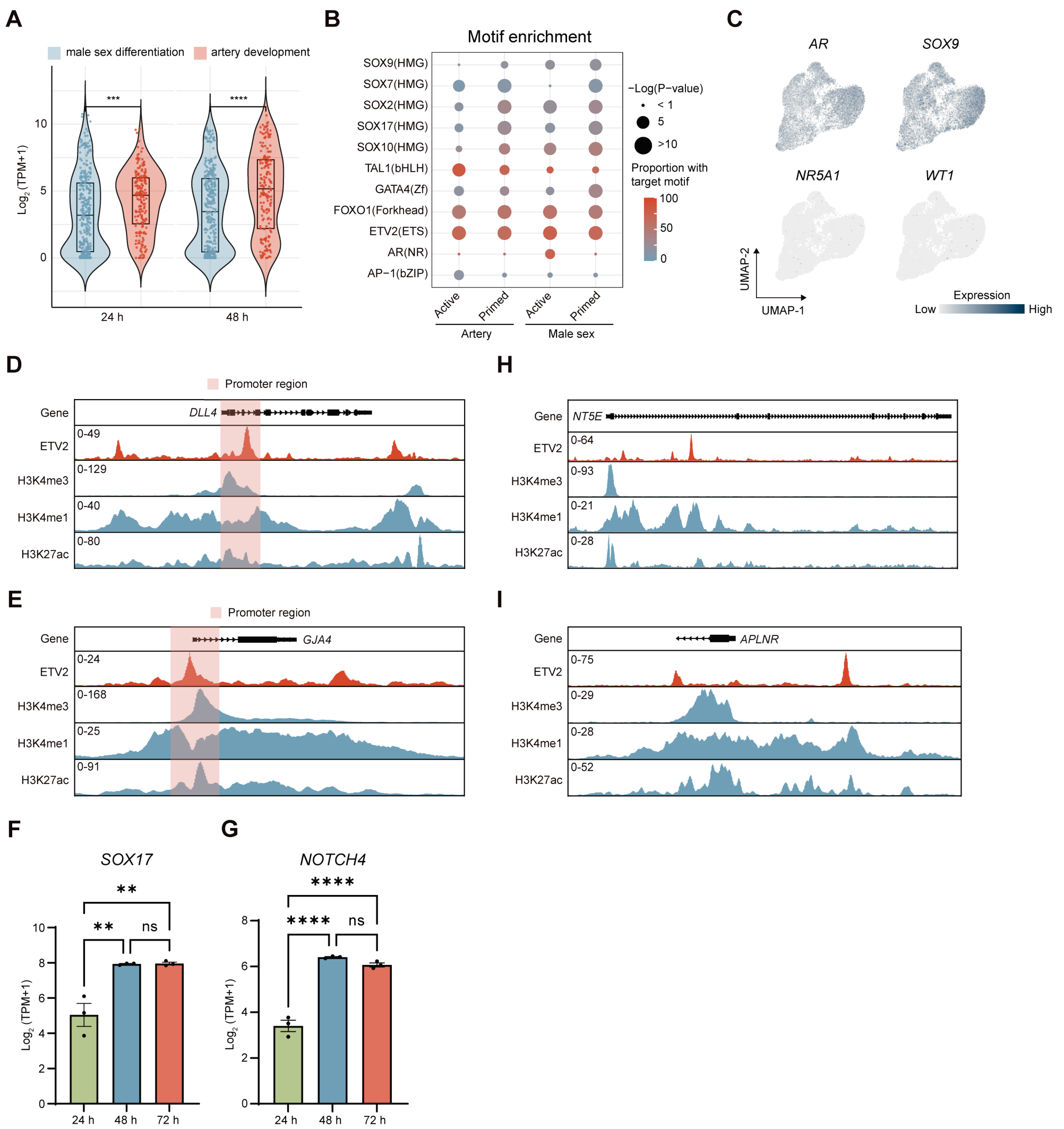

Fig. S4. ETV2 primes arterial endothelial cell fate via enhancer targeting.

(**A**) Comparison on the expression level of male sex differentiation-related or artery development-related genes regulated by ETV2. (**B**) Motif enrichment of the active enhancer regions or poised enhancer regions associated with male sex differentiation or artery development. (**C**) Uniform manifold approximation and projection (UMAP) visualization of the expression of four key transcription factors associated with male sex differentiation, *AR*, *SOX9*, *NR5A1* and *WT1* during endothelial cell differentiation. (**D**) Genome browser views showing the binding of ETV2 at the promoter of *DLL4*. (**E**) Genome browser views showing the binding of ETV2 at the promoter of *GJA4*. (**F**) Transcripts level of *SOX17* in RNA sequencing. (**G**) Transcripts level of *NOTCH4* in RNA sequencing. (**H**) Representative tracks showing minimal enrichment of H3K4me1 and H3K27ac at the venous endothelial cell (vEC)-specific marker gene *NT5E*. (**I**) Representative tracks showing minimal enrichment of H3K4me1 and H3K27ac at the vEC-specific marker gene *APLNR*. All data are presented as mean ± SEM. Statistical significance was assessed by one-way ANOVA with Tukey's post-hoc test (**F** and **G**). The level of significance was labeled as ns, *, **, ***, and ****, denoting non-significant and P-value of <0.05, <0.01, <0.001, <0.0001.

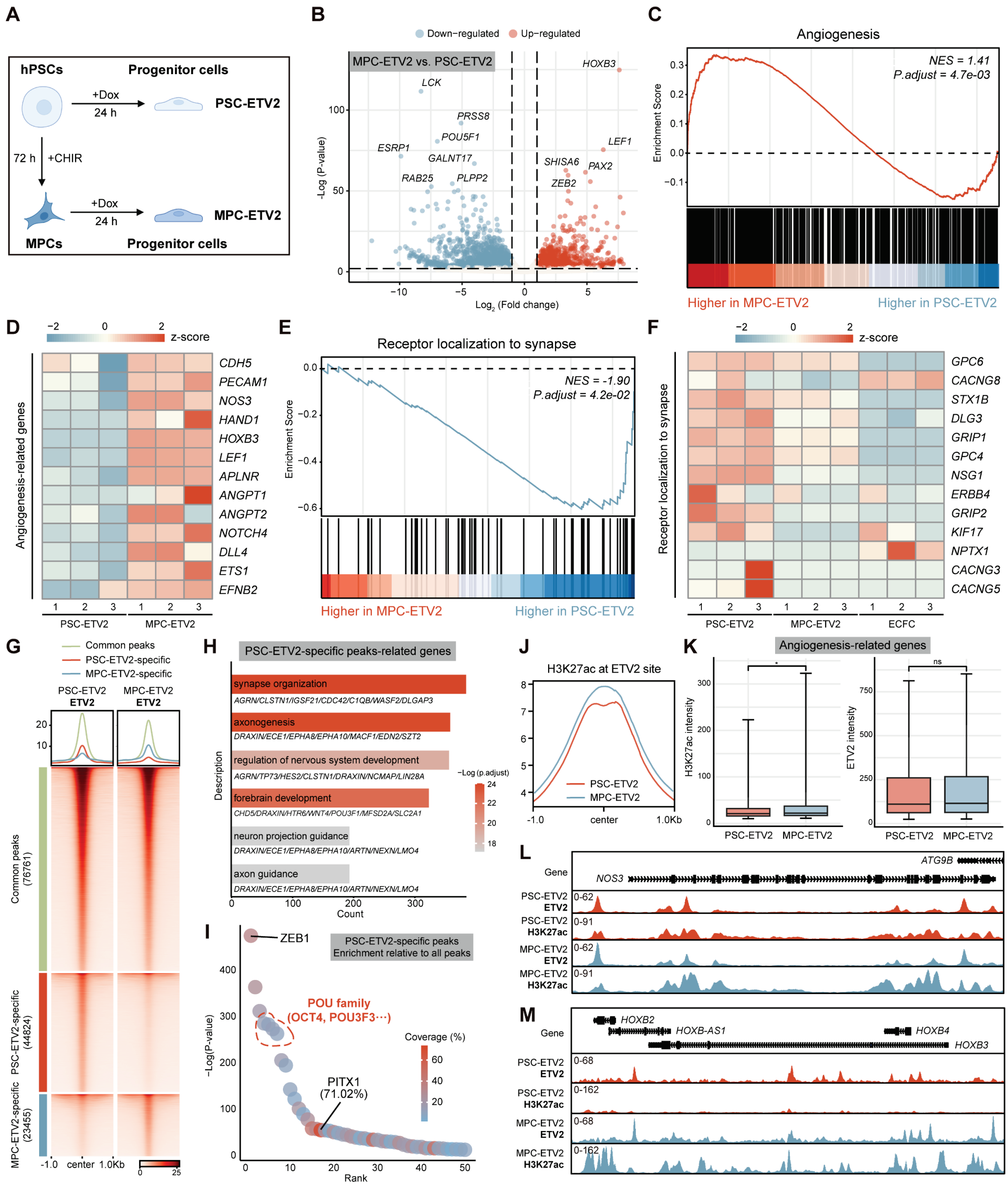

Fig. S5. MPC-ETV2 method generates functional competent endothelial cells via restriction of ETV2 binding sites.

(**A**) Schematic representation indicating the difference between PSC-ETV2 and MPC-ETV2 protocol. (**B**) Volcano plot showing genes differentially expressed between MPC-ETV2-24h cells and PSC-ETV2-24h cells. (**C**) Gene-set enrichment analysis of genes related to angiogenesis comparing gene expression in MPC-ETV2-24h cells versus PSC-ETV2-24h cells. (**D**) Heatmap showing the expression level of angiogenesis-related genes of PSC-ETV2-24h or MPC-ETV2-24h cells. (**E**) Gene-set enrichment analysis of genes related with receptor localization to synapse comparing gene expression in MPC-ETV2-24h cells versus PSC-ETV2-24h cells. (**F**) Heatmap showing the expression level of the related genes of PSC-ETV2-24h cells, MPC-

ETV2-24h cells or ECFCs. (**G**) Heatmap showing the signal of ETV2 in PSC-ETV2-24h or MPC-ETV2-24h cells at shared or specific ETV2 binding sites. (**H**) GO enrichment analysis of genes related to PSC-ETV2-specific peaks. (**I**) Motif enrichment of the PSC-ETV2-specific peaks using all ETV2 peaks in PSC-ETV2-24h cells as background. (**J**) Average signal of H3K27ac at ETV2-bound sites in PSC-ETV2-24h or MPC-ETV2-24h cells. (**K**) The intensity of H3K27ac or ETV2 ChIP-seq signal surrounding angiogenesis-related genes. (**L**) Genome browser views showing ETV2 binding sites and different H3K27ac deposition surrounding endothelial cell function-related genes *NOS3*. (**M**) Genome browser views showing ETV2 binding sites surrounding *HOXB3*. All data are presented as mean ± SEM. Statistical significance was assessed by unpaired two-tailed Student's t-test (**K**). The level of significance was labeled as ns, *, **, ***, and ****, denoting non-significant and P-value of <0.05, <0.01, <0.001, <0.0001.

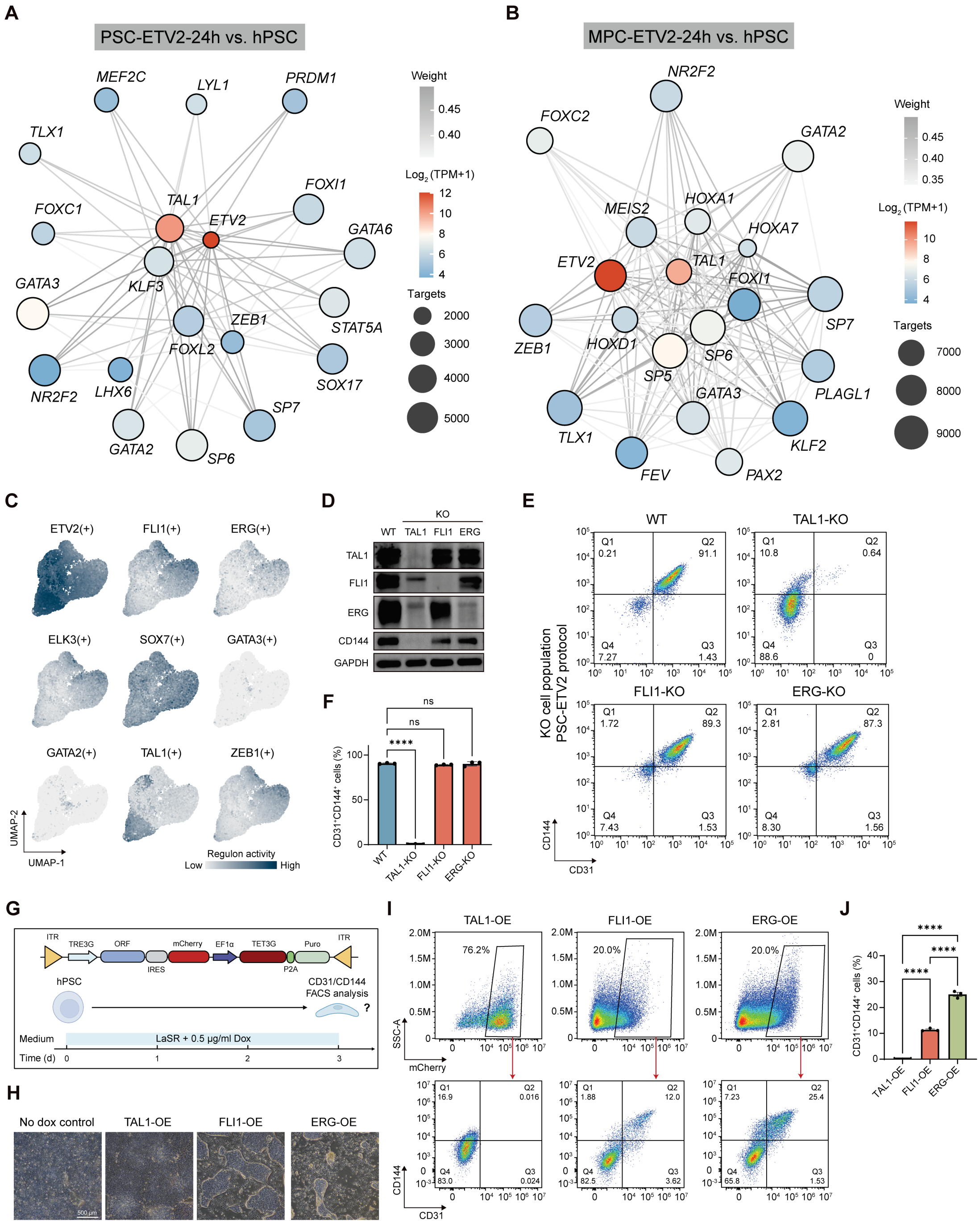

Fig. S6. Gene regulatory network in ETV2-primed cells and effects of TAL1, FLI1 and ERG in endothelial cell differentiation.

(**A**) Visualization of core gene regulatory network (GRN) in PSC-ETV2-24h cells. (**B**) Visualization of core GRN in MPC-ETV2-24h cells. (**C**) Uniform manifold approximation and projection (UMAP) visualization of regulon activities in PSC-ETV2-24h and PSC-ETV2-48h cells. (**D**) Representative Western blot imaging showing the protein level of TAL1, FLI1, ERG and CD144 after knockout. (**E**) Flow cytometric analysis of the generation of endothelial cells after knockout. (**F**) Quantification of the ratio of CD31$^+$CD144$^+$ cells (n = 3). (**G**) Schematic diagram

illustrating the overexpression of TAL1, FLI1 or ERG in hPSCs, with IRES-mCherry serving as a reporter for successful overexpression. (**H**) Representative bright-field images displaying the impact of transcription factor overexpression. Overexpression of FLI1 or ERG induce morphological changes of hPSCs. (**I**) Flow cytometric analysis evaluating the induction of endothelial cell differentiation following overexpression. (**J**) Quantification of the ratio of $CD31^{+}CD144^{+}$ cells (n = 3). Scale bar: 500 μm in (**H**). All data are presented as mean ± SEM. n indicates biological replicates (**F** and **J**). Statistical significance was assessed by one-way ANOVA with Tukey's post-hoc test (**F** and **J**). The level of significance was labeled as ns, *, **, ***, and ****, denoting non-significant and P-value of <0.05, <0.01, <0.001, <0.0001.

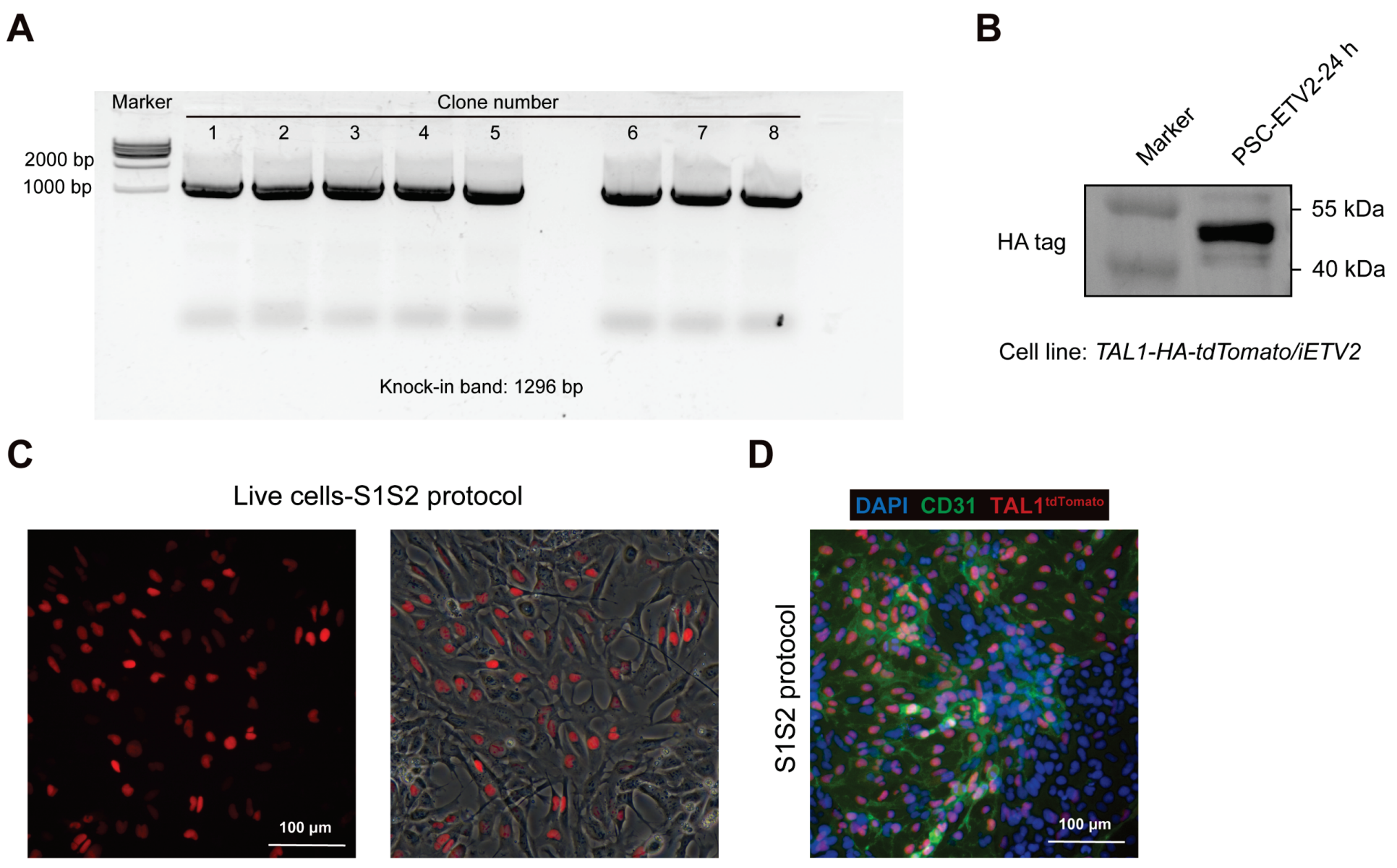

Fig. S7. Validation of the TAL1-HA-tdTomato cell line.

(**A**) Agarose gel electrophoresis showing the correct knock-in bands in different clones. (**B**) Representative Western blot imaging showing the expression of HA-tagged TAL1. (**C**) Representative fluorescent imaging showing the signal of tdTomato in live cells. (**D**) Representative immunofluorescence images revealing the co-localization of tdTomato signaling with CD31 staining. Scale bar: 100 µm in (**C** and **D**).

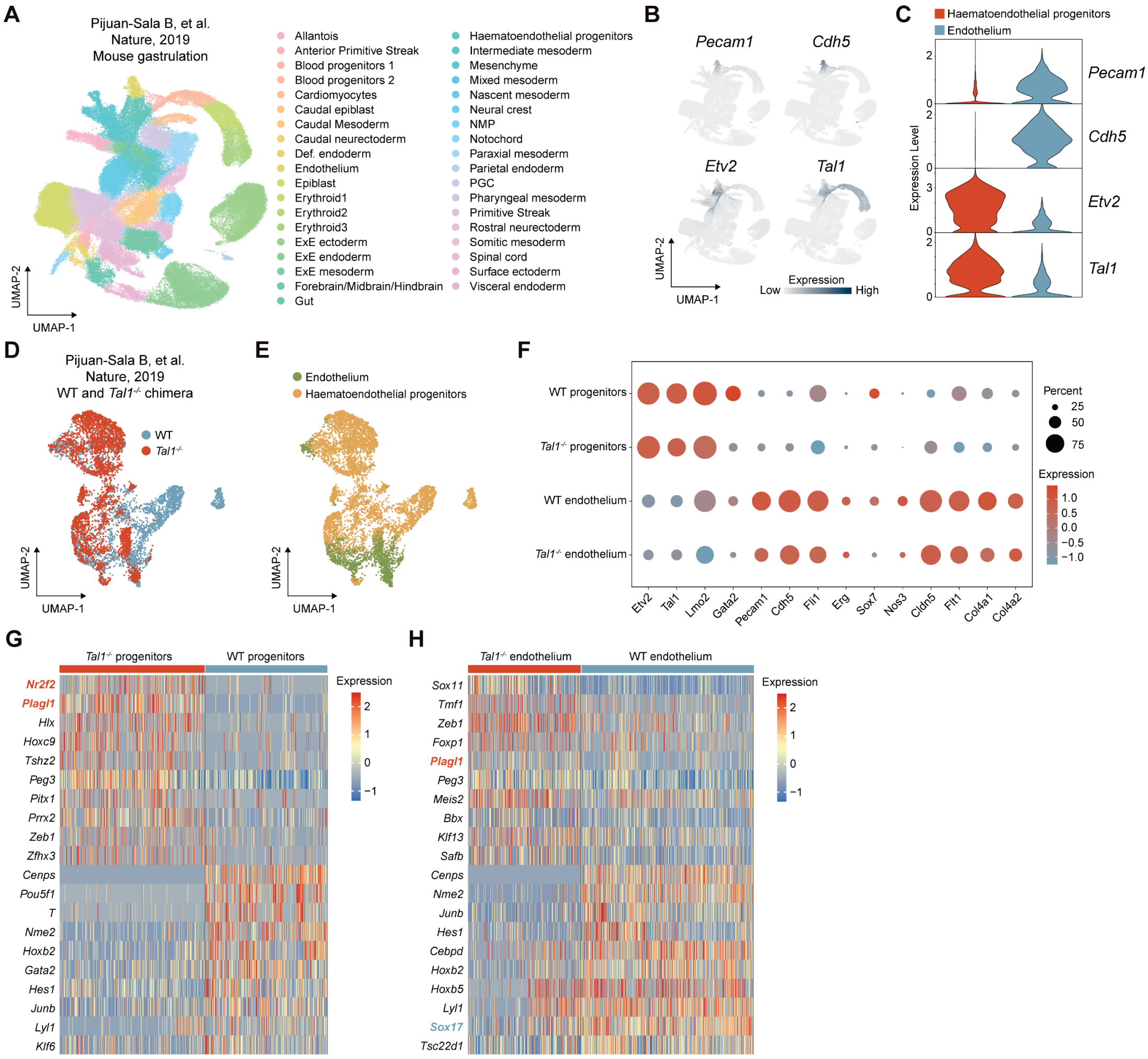

Fig. S8. Single-cell RNA sequencing analysis of the Tal1 knockout mouse chimera.

(**A**) Uniform manifold approximation and projection (UMAP) visualization of mouse gastrulation data, colored by clustering. (**B**) UMAP visualization of the expression of *Pecam1*, *Cdh5*, *Etv2* and *Tal1* in progenitor cells and endothelial cells. (**C**) Violin plot showing the expression of *Pecam1*, *Cdh5*, *Etv2* and *Tal1* in progenitor cells and endothelial cells. (**D**) UMAP visualization of chimera data, colored by samples. (**E**) UMAP visualization of chimera data, colored by cell type. (**F**) Dot plot showing the expression of endothelial marker genes in wildtype and Tal1 knockout chimeras. (**G**) Heatmap showing the differential expressed transcription factors in progenitor cells. (**H**) Heatmap showing the differential expressed transcription factors in endothelial cells.

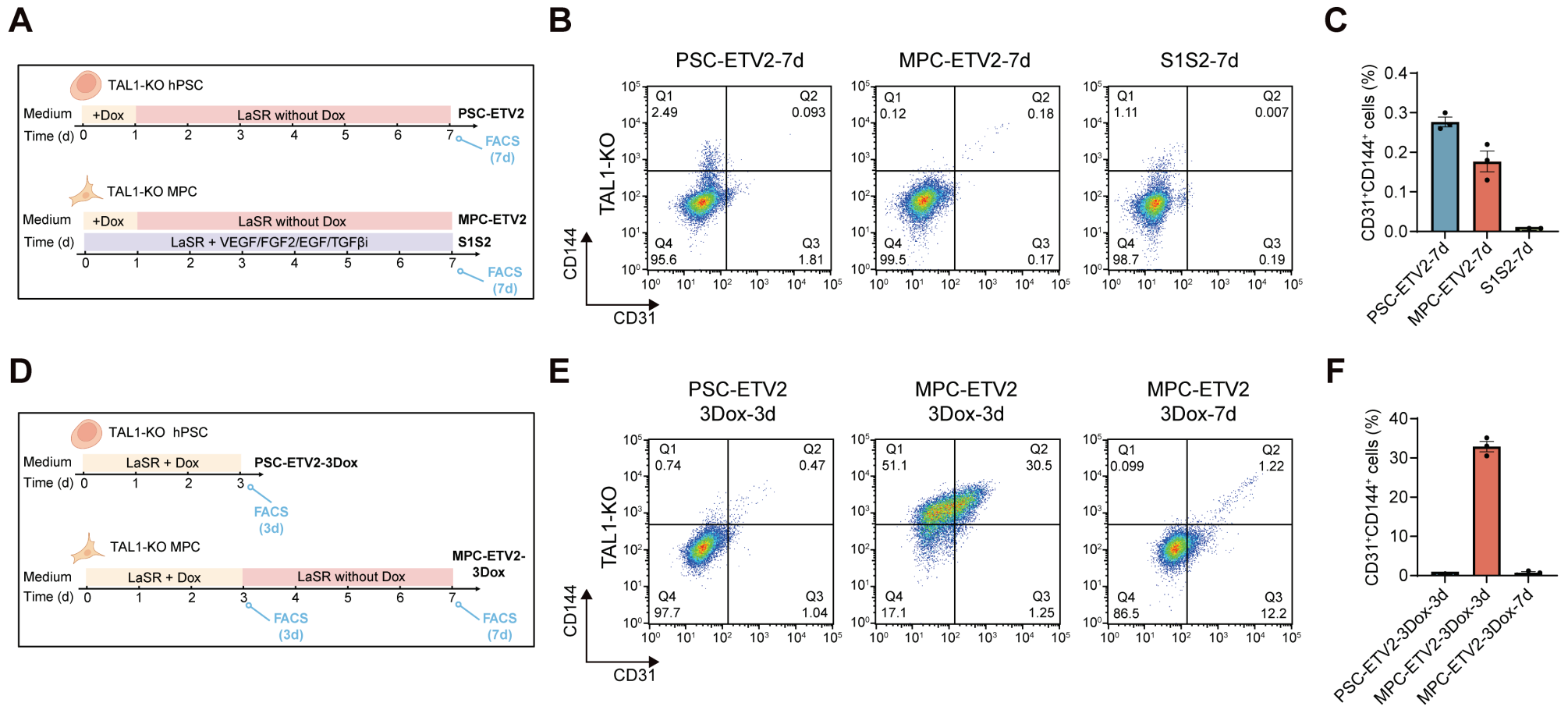

Fig. S9. Prolonged culture or extend the overexpression window cannot rescue ETV2-primed endothelial cell differentiation.

(**A**) Schematic representation of the experimental design for extending the differentiation to seven days. (**B**) Flow cytometric analysis of the generation of endothelial cells on day 7 using PSC-ETV2, MPC-ETV2 and S1S2 protocols. (**C**) Quantification of the ratio of $CD31^{+}CD144^{+}$ cells after extended culture (*n* = 3). (**D**) Schematic representation of extending the doxycycline induction to three days. (**E**) Flow cytometric analysis of the generation of endothelial cells following the extended ETV2 induction window using PSC-ETV2 and MPC-ETV2 protocols. (**F**) Quantification of the ratio of $CD31^{+}CD144^{+}$ cells after extending the ETV2 overexpression window (*n* = 3). All data are presented as mean ± SEM. *n* indicates biological replicates (**C** and **F**).

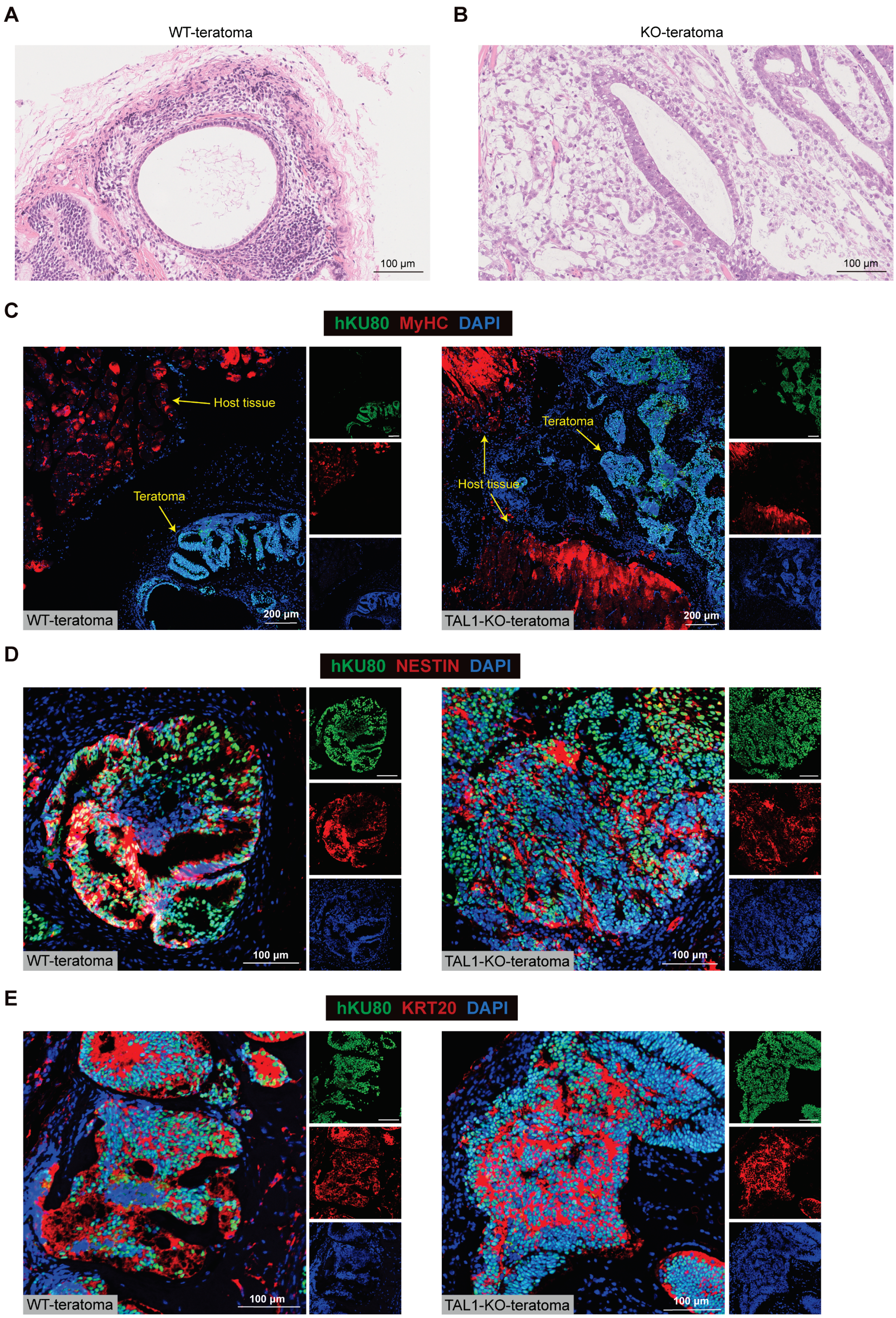

Fig. S10. TAL1 deficiency does not impair the multi-lineage differentiation potential of hPSCs.

(**A**) Hematoxylin and Eosin (H&E) staining of the wildtype (WT) human pluripotent stem cell (hPSC)-derived teratoma. (**B**) H&E staining of the TAL1 knockout (KO)

hPSC-derived teratoma. (**C**) Representative immunofluorescence images of myosin heavy chain (MyHC) and hKU80 in wildtype (WT) and TAL1-KO hPSC-derived teratoma. (**D**) Representative immunofluorescence images of NESTIN and hKU80 in WT and TAL1-KO hPSC-derived teratoma. (**E**) Representative immunofluorescence images of KRT20 and hKU80 in WT and TAL1-KO hPSC-derived teratoma. Scale bar: 100 μm in (**A**, **B**, **D** and **E**) and 200 μm in (**C**).

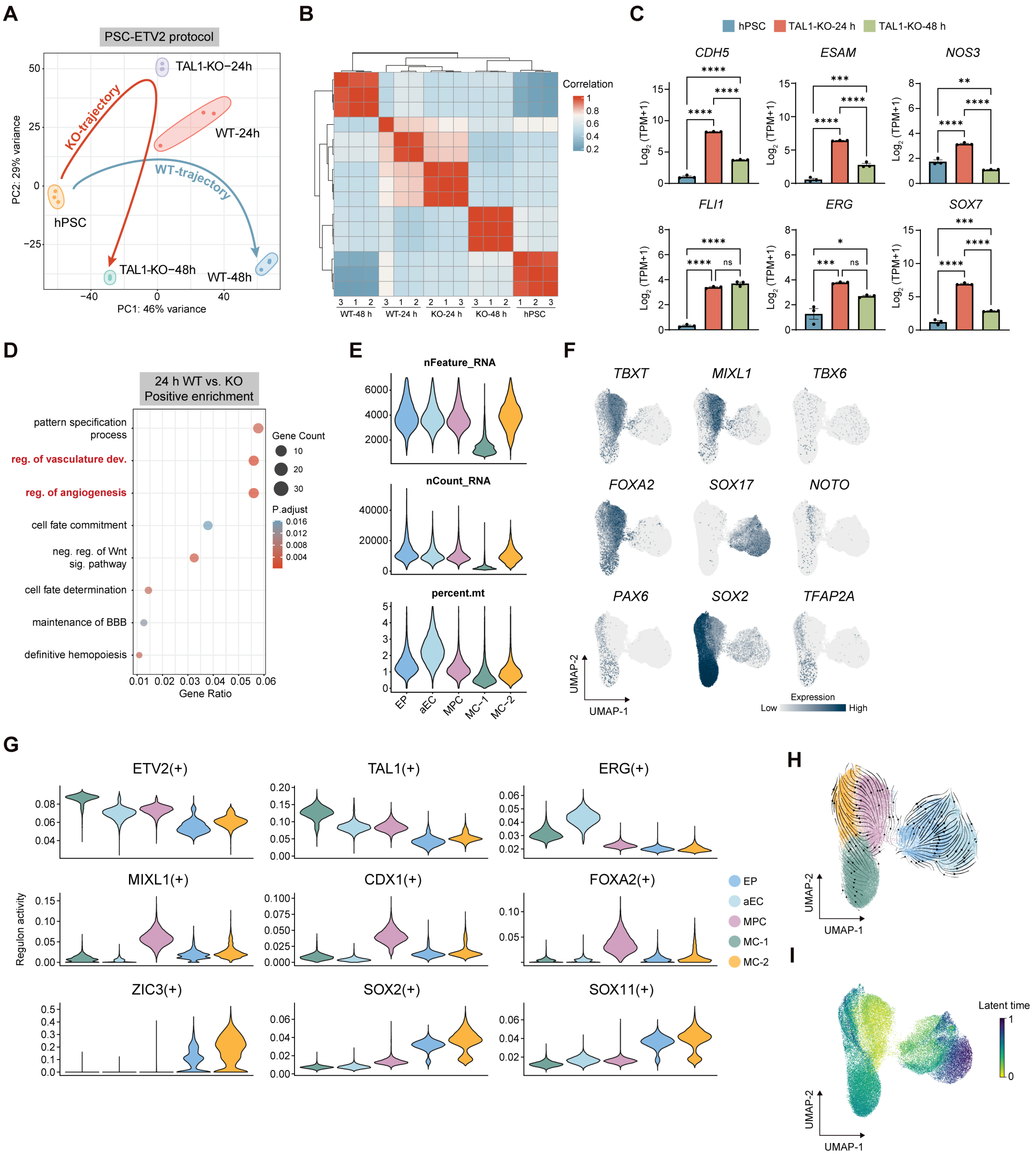

Fig. S11. Transcriptome analysis of TAL1 knockout cells.

(**A**) Principal component analysis of the transcriptome of WT and KO cells. (**B**) Correlation analysis of the WT and KO cells. (**C**) Transcripts level of *CDH5*, *ESAM*, *NOS3*, *FLI1*, *ERG* and *SOX7* in RNA sequencing, showing the initial ETV2 effect in the absence of TAL1. (**D**) Gene ontology enrichment analysis of the upregulated genes in WT-24h cells. (**E**) Quality control metrics of single-cell RNA sequencing data. Number of genes (nFeature_RNA), number of molecular (nCount_RNA), and percentage of mitochondrial counts (percent.mt) is plotted for each cluster. (**F**) Uniform manifold approximation and projection (UMAP) visualization of the expression of several endoderm, mesoderm and ectoderm-relate genes. (**G**) Regulon activity analysis of WT and KO cells. (**H**) RNA velocity analysis of WT and KO cells. (**I**) Latent time analysis of WT and KO cells. All data are presented as mean ± SEM. Statistical significance was assessed by one-way ANOVA with Tukey's post-

hoc test (**C**). The level of significance was labeled as ns, *, **, ***, and ****, denoting non-significant and P-value of <0.05, <0.01, <0.001, <0.0001.

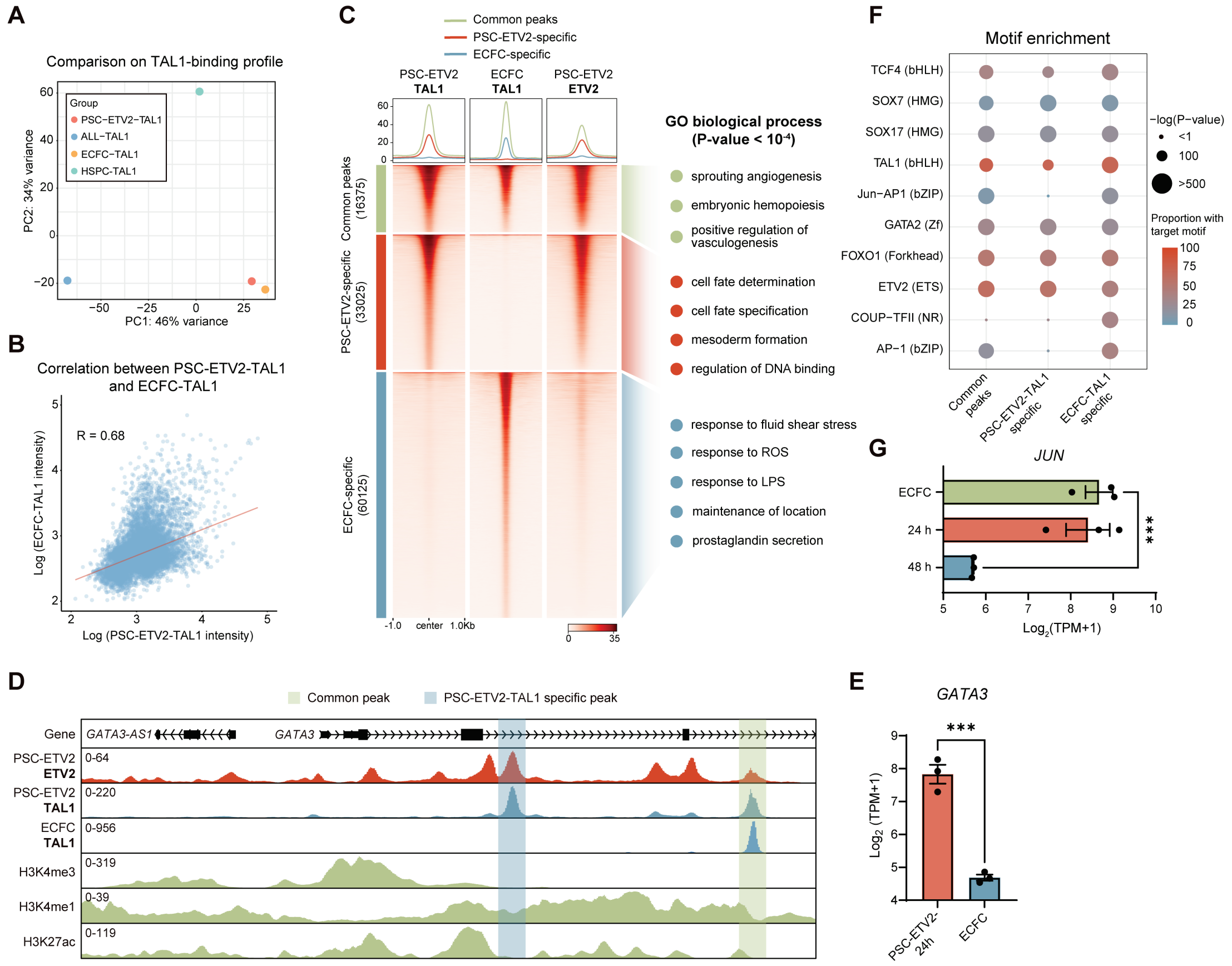

Fig. S12. Comparison on TAL1-binding profile in several cell types.

(**A**) Principal component analysis to compare the TAL1-binding profile in PSC-ETV2-24h cells, acute lymphoblastic leukemia (ALL) cells, endothelial colony forming cells (ECFCs) and hematopoietic stem and progenitor cells (HSPCs). (**B**) The correlation of TAL1-binding profile between PSC-ETV2-24h cells and ECFCs. (**C**) Heatmap showing the signal of ETV2 and TAL1 at common, PSC-ETV2-specific or ECFC-specific TAL1 binding sites and the related GO terms. **(D)** Genome browser view showing the specific TAL1 binding site at the intragenic region of *GATA3* in PSC-ETV2-24h cells. (**E**) Comparison of the expression level of *GATA3* in PSC-ETV2-24h cells and ECFCs. (**F**) Motif enrichment on the related regions. (**G**) The expression level of *JUN* in ECFCs or PSC-ETV2-24h, 48h cells. All data are presented as mean ± SEM. Statistical significance was assessed by unpaired two-tailed Student's t-test (**E**) and one-way ANOVA with Tukey's post-hoc test (**G**). The level of significance was labeled as ns, *, **, ***, and ****, denoting non-significant and P-value of <0.05, <0.01, <0.001, <0.0001.

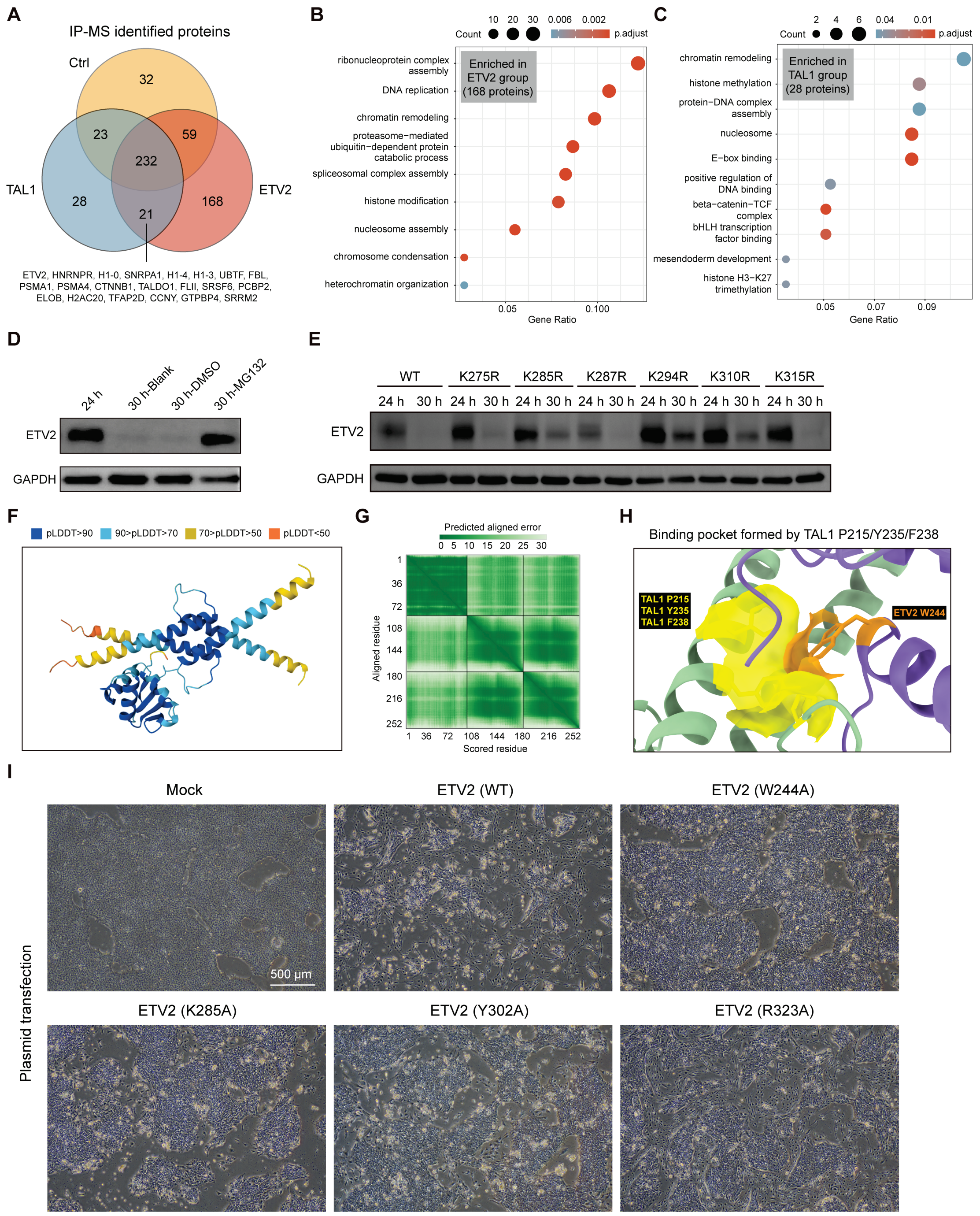

Fig. S13. ETV2 and TAL1 interactome and structural basis of the interaction.

(**A**) Venn diagram showing the intersection of the IP-MS identified protein in three group. The 21 proteins co-recruited by ETV2 and TAL1 are listed below. (**B**) Gene ontology (GO) enrichment analysis on the proteins which are significant in ETV2 group. (**C**) GO enrichment analysis on the proteins which are significant in TAL1 group. (**D**) Representative Western blot imaging showing the delayed degradation of ETV2. (**E**) Representative Western blot imaging showing the degradation of ETV2 of different mutants. (**F**) AlphaFold3-predicted structure of the ETV2-TAL1 complex, colored by pLDDT score to indicate model confidence. (**G**) Predicted Aligned Error

(PAE) plot evaluating the relative positional certainty of the predicted structure. (**H**) Structural representation of the hydrophobic interface stabilizing the ETV2-TAL1 interaction. (**I**) Representative bright field images showing some ETV2 mutants failed to generate endothelial cells. Scale bar: 500 μm in (**I**).

Table S1. Primers for qPCR and genotyping

**Gene**	**Forward primer (5'-3')**	**Reverse primer (5'-3')**
**qPCR primers**		
ETV2	GAAGGAGCCAAATTAGGCTTCT	GAGCTTGTACCTTTCCAGCAT
TAL1	GTGCAGCTGAGTCCTCCCGC	CCGGCTCCCCAAAGAACCCG
FLI1	CAGCCCCACAAGATCAACCC	ACCGGAGACTCCCTGGAT
ERG	CGTGCCAGCAGATCCTACG	GGTGAGCCTCTGGAAGTCG
SOX7	TCGACGCCCTGGATCAACT	CTGGGAGACCGGAACATGC
ELK3	GAGAGTGCAATCACGCTGTG	GTTCGAGGTCCAGCAGATCAA
MECOM	TATCCACGAAGAACGGCAATATC	CATGGAAACTTTTGGTGATCTGC
ETS1	GATAGTTGTGATCGCCTCACC	GTCCTCTGAGTCGAAGCTGTC
GATA2	GCAACCCCTACTATGCCAACC	CAGTGGCGTCTTGGAGAAG
GATA3	GCCCCTCATTAAGCCCAAG	TTGTGGTGGTCTGACAGTTCG
SOX17	GTGGACCGCACGGAATTTG	GGAGATTCACACCGGAGTCA
HEY1	GTTCGGCTCTAGGTTCCATGT	CGTCGGCGCTTCTCAATTATTC
GAPDH	ACAACTTTGGTATCGTGGAAGG	GCCATCACGCCACAGTTTC
**PCR amplification for plasmid construction and genotyping primers**		
TAL1-F/R	ACATACGGTCCCTGTCCTCA	CCCCAGGGCAAGAAGTAAGG
TAL1-HAL-F/R	ACATACGGTCCCTGTCCTCA	CCGAGGGCCGGCTCC
TAL1-HAR-F/R	TGGGTCTGGGCCACCAGG	CCCCAGGGCAAGAAGTAAGG
TAL1-G-F/R	AGCCACTGGAGCTAAACATGG	GTCTCCCGCTTGTTTCAGTAGA

Table S2. sgRNA sequence for knockout and knockin

**Name**	**sgRNA sequence**
TAL1-KO	GAAGATACGCCGCACAACTT
FLI1-KO	gCGCTTGACGTTGACCCTCAC
ERG-KO	gTGCCTACGGAACGCCACACC
TAL1-KI	gTGGAGCCGGCCCTCGGTGAT
